# Silver-Promoted Radical Cascade Aryldifluoromethylation/Cyclization of 2-Allyloxybenzaldehydes for the Synthesis of 3-Aryldifluoromethyl-Containing Chroman-4-one Derivatives

**DOI:** 10.3390/molecules28083578

**Published:** 2023-04-19

**Authors:** Qianqian Sun, Hongxiao Li, Xingyu Chen, Jian Hao, Hongmei Deng, Haizhen Jiang

**Affiliations:** 1 Department of Chemistry, Shanghai University, Shanghai 200444, China; 2 Laboratory for Microstructures, Shanghai University, Shanghai 200444, China; 3 Key Laboratory of Organofluorine Chemistry, Shanghai Institute of Organic Chemistry, Chinese Academy of Sciences, Shanghai 200032, China

**Keywords:** radical cascade, aryldifluoromethylation/cyclization, chroman-4-one derivatives

## Abstract

A convenient silver-promoted radical cascade aryldifluoromethylation/cyclization of 2-allyloxybenzaldehydes has been developed. Experimental studies disclosed that the addition of aryldifluoromethyl radicals in situ produced from easily accessible *gem*-difluoroarylacetic acids to unactivated double bonds in 2-allyloxybenzaldehyde was an effective route to access a series of 3-aryldifluoromethyl-containing chroman-4-one derivatives in moderate to good yields under mild reaction conditions.

## 1. Introduction

**Figure 1:**
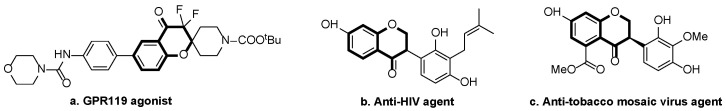
Examples of the bioactive compounds with a 3-substituent chroman-4-one moiety.

The chroman-4-one framework belongs to the privileged class of heterocycles and acts as a major building block in many natural products, pharmaceuticals, and biologically active compounds [1,2]. In particular, the compounds with substituents at the 3-position of the chroman-4-one exhibit significant physiological and biological activities, such as GPR119 agonist (Figure 1, **a**) [3], anti-HIV agent (Figure 1, **b**) [4], and anti-tobacco mosaic virus agent (Figure 1, **c**) [5] etc. Thus, a number of approaches for the synthesis of 3-substituted chroman-4-one derivatives have been developed, such as transition-metal-catalyzed cross-coupling reaction [6], Stetter reaction [7], and cascade radical cyclization [8,9,10,11,12].

The introduction of difluoromethylene group into organic molecules can not only improve chemical, physical properties and the physiological activity of the parent molecule, but also affect their binding affinity, metabolic stability, lipophilicity, and membrane permeability and bioactivity, as well as modulate pharmacokinetic behavior and penetration in biological tissues [13,14,15]. Hence, *gem*-difluoromethylene group (CF_2_) is regarded as a valuable fluorinated moiety that is extensively present in pharmaceuticals, biologically active compounds, and material molecules [16,17]. Therefore, the development of effective and general methodologies for the selective incorporation of a difluoromethylene group has become one of the hotspots in the field of organic chemistry. Traditional methods for the synthesis of the difluoromethylene-containing compounds include the direct deoxyfluorination of the carbonyl group in corresponding aldehydes or ketones by using bis(2-methoxyethyl)aminosulfur trifluoride or DAST ((C_2_H_5_)_2_NSF_3_) [18,19,20,21] and the conversion of building blocks containing difluoromethylene under specific conditions. For example, visible light-promoted the difluoromethylenation of C–H bonds under xanthone catalyzed conditions [22,23,24], and transition-metal-catalyzed (Pd- or Ni-) difluoromethylenation can be achieved applying chlorodifluoromethane [25], and so on. However, some existing methods suffer from either functional group incompatibility or the use of toxic and explosive reagents [26,27,28]. Therefore, it remains highly desired to develop practical and mild synthetic methods by using stability and easily available substrates containing-difluoromethylene building blocks, such as α,α-difluoroarylacetic acids [29,30,31,32].

**Scheme 1:**
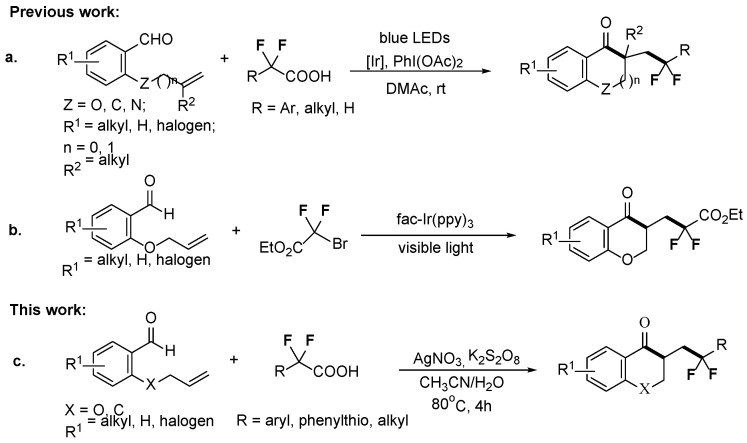
Synthesis of 3-difluoromethylene-containing chroman-4-one derivatives.

Radical cascade fluoroalkylation/cyclization of alkenes has been proved to be one of the effective methods to obtain fluoroalkyl-containing heterocycle products due to the advantages that it avoids the separation and purification of intermediates [33] and realizes the introduction a fluoroalkyl group while constructing the diversity and complexity of heterocyclic skeletons. However, some fluoroalkyl radicals, such as CF_3_ radical, CF_2_H, and C_n_F_2n+1_ radical, are electron-deficient σ-radicals and thus prone to add to electron-rich alkenes. Due to electronically unfavorable conditions, it is difficult to realize the free radical cascade fluoroalkylation/cyclization of unactivated or electron-deficient alkenes. Therefore, there are only few reports on the cascade difluoromethylenation/cyclization regarding the synthesis of 3-difluoromethylene-containing chroman-4-one derivatives through the addition difluoromethyl radicals to unactivated alkenes in 2-allyloxybenzaldehydes. The reported methods include that of Zhu’s group, which developed a visible light photocatalytic aryldifluoromethylation/cyclization of alkenes using α,α-difluoroarylacetic acids (Scheme 1a) [34], and Zhou’s group and Ma’s group, which described radical cascade cyclization of alkene aldehydes using a 1-bromo-2-difluoro-containing building block (Scheme 1b) [35,36] under the visible-light photoredox catalysis conditions. Therefore, it is highly desirable to develop new, convenient methods for the synthesis of 3-difluoromethylene-containing chroman-4-one derivatives, to avoid using a relatively expensive photocatalyst in the mild reaction. Our previous research on the decarboxylation of *gem*-difluoroarylacetic acids under silver-catalyzed condition revealed that aryldifluoromethyl radicals have a slightly electron-rich property relative to the CF_3_ radical and could react with the electron-deficient activated double bond in allyl sulfones [37]. Herein, we further tried to use aryldifluoromethyl radicals from the decarboxylation of *gem*-difluoroarylacetic acids under the promotion of the catalytic amount of silver to explore the cascade aryldifluoromethylation/cyclization reaction with unactivated double bonds in 2-allyloxybenzaldehydes to synthesize 3-aryldifluoromethyl-containing chroman-4-one derivatives (Scheme 1c).

## 2. Results and Discussion

Our previous research found that the system of silver salts and persulfate salts was effective in the decarboxylation of difluoroarylacetic acids in cross-coupling reactions with allyl sulfones [37]. Therefore, 4-methyl difluorophenylacetic acid **2a** and 2-(allyloxy)benzaldehyde **1a** which included unactivated double bonds were chosen as model substrates to participate in the reaction in the presence of AgNO_3_ (20 mol%) and K_2_S_2_O_8_ (2 equiv.) in CH_3_CN/H_2_O (*v*/*v* = 1:1) at 80 °C under a N_2_ atmosphere, replacing visible light redox catalytic conditions [34]. To our delight, 3-(4-CH_3_)phenyldifluoromethyl-containing chroman-4-one product **3aa** was observed in 56% ^19^F NMR yield (Table 1, entry 1). Encouraged by the result, we try to optimize the reaction conditions. When solvent was shifted from CH_3_CN/H_2_O to EtOH/H_2_O or DMSO/H_2_O, the reaction gave the desired product in lower yields (Entries 2 and 3). The product **3aa** was also produced when the solvent was pure H_2_O, but the reaction efficiency was reduced (Entry 4). When the solvent was DMSO, the reaction could also produce the product in 32% yield (Entry 22). However, the desired product was obtained if the reaction was carried out in CH_3_CN (Entry 5). Upon further screening of solvents, it was found that the reaction provided the highest yield (73%) in the solvent CH_3_CN/H_2_O (*v*/*v* = 1:3) when 2 equivalents of K_2_S_2_O_8_ were used as oxidant (Entry 6). Other oxidants, including Na_2_S_2_O_8_ and (NH_4_)_2_S_2_O_8_, were also effective (Entries 7 and 8). In contrast, selectfluor failed to carry out this transformation (Entry 9). Additionally, when changing the amount of oxidant K_2_S_2_O_8_ from 2 into 1.75 or 2.25 equiv., the reaction efficiency was slightly reduced (Entry 6 vs 17 and 18). Moreover, oxidant K_2_S_2_O_8_ was actually required to bring about the cascade aryldifluoromethylation/cyclization (Entry 11). We also further evaluated the effect of the amount of AgNO_3_ on the reaction. AgNO_3_ could promote the reaction, but it was not necessary (Entry 10). A higher catalyst loading of AgNO_3_ did not lead to a higher reaction efficiency, and 10 mol% of AgNO_3_ as promoter resulted in 63% yield of **3aa** (Entries 12 and 13). The other Ag (I) sources, such as AgOAc, Ag_2_O, or Ag_2_CO_3_, could also be used as the promoters, but they were less effective (Entries 19–21). Adjusting the ratio of **1a** and **2a**, it was found that **3aa** was provided in the highest yield when the ratio of **1a** and **2a** was 1:1.5 (Entries 6, 14–16) at 80 °C for 4 h (Supplementary Materials).

**Figure 2:**
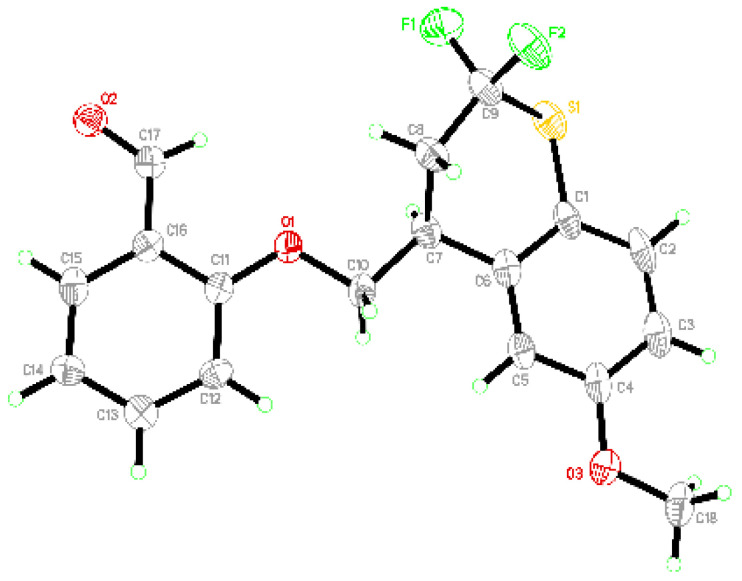
X-ray crystallography of **3aq′** (gray for carbon atoms, green for fluorine atom, yellow for sulfur atom and red for oxygen atom).

Having established the optimal experimental conditions, we explored the scope and limitation of this cascade aryldifluoromethylation/cyclization by the reaction of α,α-difluoroarylacetic acids and 2-allyloxybenzaldehydes. We first explored the scope with respect to the α,α-difluoroarylacetic acids **2**, as shown in Table 2. Difluoroarylacetic acids (**2a**–**2h**) bearing an electron-donating group, such as CH_3_, OCH_3_ etc., at different positions of benzene ring produced the desired product in moderate to excellent yields (**3aa**–**3ah**). When 2,2-difluoro-2-phenylacetic acid **2i** was used, the reaction gave the desired product **3ai** in 50% yield. The sterically hindered 2,2-difluoro-2-mesitylacetic acid **2h** was also a compatible substrate in the cascade aryldifluoromethylation/cyclization leading to **3ah** in 55% yield. Additionally, the α,α-difluoroarylacetic acids with different halogen groups at position-4 of the benzene ring could offer the desired products in 32 to 60% yields (**3ak**–**am** and **3ao**), and the halogen groups were well tolerated. However, no desired product **3ap** was obtained when the *para*-substituent of benzene ring in the α,α-difluoroarylacetic acid is strong electron-withdrawing nitro group. Difluoroarylacetic acids bearing a strong electron-withdrawing group, such as CN or CF_3_ at benzene ring, also made it difficult to provide the desired product. The results showed that the substituents on the benzene ring of α,α-difluoroarylacetic acid have a great influence on the reaction activity. This could be attributed to the fact that the electron-donating group substituted aryldifluoromethyl radicals produced in situ under silver-promoted condition demonstrated greater electron-rich property than that of non-substituted or strong electron-withdrawing group substituted phenyldifluoromethyl radical. Thus, the electron-richer aryldifluoromethyl radicals were capable of facilitating addition to unactivated double bonds in 2-allyloxybenzaldehyde. To further investigate the reaction scope, 2,2-difluoro-2-((4-methoxyphenyl)thio)acetic acid **2q** was also employed as substrate, and an unexpected regio-selective product, **3aq′**, was achieved in 29% yield besides the desired product **3aq** in 30% yield. The structure of **3aq′** was further characterized by X-ray single crystal diffraction analysis (Figure 2) and the NMR and HR-MS etc. Additionally, when aliphatic 2,2-difluorobutanoic acid (**2r**) was employed in this transformation, the reaction also gave the desired product **3ar** in 13% yield.

The generality of this cascade aryldifluoromethylation/cyclization toward different 2-allyloxybenzaldehydes was also explored. As shown in Table 3, a variety of 2-allyloxybenzaldehydes **1** were employed to react with 2,2-difluoro-2-(4-methoxyphenyl)acetic acid (**2b**). This method successfully gave the corresponding products **3** in moderate to good yields. The substrates **1b**–**d** bearing electron-donating groups reacted smoothly, delivering the corresponding chroman-4-one derivatives **3bb**–**3db** in moderate yields (35–50%). Unexpectedly, the reaction of the di-*t*-Bu-substituted 2-allyloxybenzaldehyde (**1e**) provided the corresponding product **3eb** in trace amount in the aqueous CH_3_CN solution (CH_3_CN:H_2_O = 1:3) under standard conditions, while it delivered **3eb** in 45% yield in the DMSO. The substrates monosubstituted by different halogens at different positions of the benzene ring in 2-allyloxybenzaldehydes (**1f**–**1k**) gave the desired product in moderate yields as well. Additionally, halogen functional groups were well tolerated in this protocol. The di-bromo-substituted substrate **1m** was not a good substrate for this transformation. However, the cascade aryldifluoromethylation/cyclization of 2-((2-methylallyl)oxy) benzaldehyde **1m** could afford the product **3mb** in 41% in DMSO. Notably, the reaction of 2-allylbenzaldehyde **1n** with 2,2-difluoro-2-(4-methylphenyl)acetic acid (**2a**) could also deliver the corresponding product **3na** in 37% yield.

**Scheme 2:**
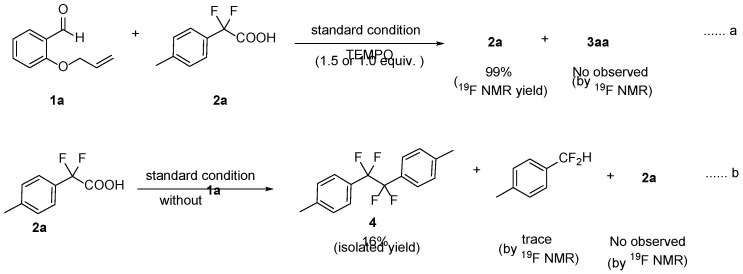
Control experiments.

To gain more insight into the reaction mechanism and identify whether a radical pathway is involved in the silver promoted cascade aryldifluoromethylation/cyclization or not, some control experiments were carried out. When 1.0 or 1.5 equiv. of radical scavenger TEMPO were added into the reaction system of 4-methyl difluorophenylacetic acid (**2a**) and 2-allyloxybenzaldehyde **1a** under the standard conditions, the reaction provided neither the desired product **3aa** nor adduct of TEMPO with phenyldifluoromethyl radical based on ^19^F NMR analysis. Additionally, the conversion of **2a** was completely inhibited under the standard conditions because **2a** almost totally remained in the above reaction system by ^19^F NMR monitoring (Scheme 2a). We deduced from the results that the reaction may undergo a radical process. To further capture the phenyl difluoromethyl radical to verify this speculation, the reaction of only 4-methyl difluorophenylacetic acid (**2a**) used as substrate under the standard conditions in the absence of 2-allyloxybenzaldehyde **1a** was performed (Scheme 2b). The phenyl difluoromethyl radical self-coupling product **4** was obtained in 16% isolated yield. Thus, it was indicated that the phenyl difluoromethyl radical as intermediate could be involved in the reaction process (Scheme 2).

**Scheme 3:**
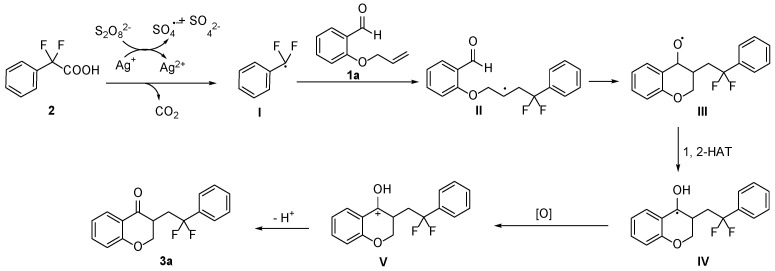
Plausible reaction mechanism.

On the basis of the above-mentioned results and previous reports [35,37,38,39,40,41,42], a possible mechanism for the reaction is depicted in Scheme 3. First, α,α-difluorophenylacetic acid **2** undergoes an oxidative decarboxylation process under the promotion of Ag(I) to give the corresponding phenyldifluoromethyl radical **I** [43]. Then, the addition of **I** to the unactivated C=C double bond of 2-allyloxybenzaldehyde **1a** affords an alkyl radical **II**. The alkyl radical intermediate **II** attacking the C-O double bond led to an alkoxy radical intermediate **IIl**, realizing an intramolecular cyclization. Afterward, a formal 1,2-hydrogen atom transfer produces the α-hydroxy carbon-centered radical **IV**. Subsequently, radical **IV** is oxidized to give the carbocation intermediate **V** [43]. At last, the proton transfer of intermediate **V** leads to the final product **3a**. The product **3aq’** could be formed by the cyclization of the alkyl radical intermediate **II’** arising from a process similar to that of radical intermediate **II** to the benzene ring of **2q** instead of attacking the C-O double bond.

## 3. Conclusions

In conclusion, we established a facile radical cascade aryldifluoromethylation/cyclization method to synthesize a series of 3-aryldifluoromethyl-containing chroman-4-one derivatives in moderate to good yields. These reactions involved the addition of aryldifluoromethyl radicals in situ produced from easily accessible *gem*-difluoroarylacetic acids to unactivated double bonds in 2-allyloxybenzaldehydes under silver-promoted conditions. This benign protocol replaces the use of expensive photocatalysts in visible light redox catalytic conditions and provides a new perspective to construct pharmaceutically valuable, 3-difluoromethylene-containing chroman-4-one derivatives.

## 4. Materials and Methods

### General Procedure for the Synthesis of Compounds **3**

2-Allyloxyarylaldehyde **1** (0.20 mmol), K_2_S_2_O_8_ (2.0 equiv, 0.40 mmol), AgNO_3_ (20 mol%, 0.04 mmol) and α,α-difluoroarylacetic acid **2** (1.5 equiv, 0.30 mmol) were placed in a 10-mL round-bottom flask. Solvent CH_3_CN/H_2_O (*v*/*v* = 1:3, 2.0 mL) were then added under nitrogen atmosphere. The solution was stirred at 80 °C for 4 h. The resulting mixture was cooled down to room temperature and extracted with EA (5 mL × 4). The combined organic phase was dried over anhydrous Na_2_SO_4_. After the removal of solvent under reduced pressure, the crude product was purified by silica gel column chromatography (petroleum ether/ethyl acetate) to afford pure 3-difluoromethylene-containing chroman-4-one **3**.

*3-(2,2-Difluoro-2-(m-tolyl)ethyl)chroman-4-one* (**3ae**), 35.1 mg, 58% yield, light yellow solid; mp 67.3–67.6 ^o^C; ^1^H NMR (500 MHz, CDCl_3_) δ 7.89 (dd, *J* = 7.9, 1.7 Hz, 1H), 7.47 (ddd, *J* = 8.5, 7.2, 1.8 Hz, 1H), 7.36–7.30 (m, 3H), 7.25 (s, 1H), 7.01 (t, *J* = 8.0 Hz, 1H), 6.97 (d, *J* = 9.0 Hz, 1H), 4.76 (dd, *J* = 11.4, 5.1 Hz, 1H), 4.26 (t, *J* = 11.7 Hz, 1H), 3.24–2.96 (m, 2H), 2.40 (s, 3H), 2.19–1.93 (m, 1H); ^19^F NMR (470 MHz, CDCl_3_) δ −89.87 (ddd, *J* = 245.2, 24.6, 8.1 Hz), −97.24 (ddd, *J* = 245.1, 22.3, 16.2 Hz); ^13^C NMR (125 MHz, CDCl_3_) δ 192.1, 161.6, 138.5, 136.5 (t, ^2^*J*_C-F_ = 26.0 Hz), 136.0, 130.9, 128.6, 127.5, 125.5 (t, ^3^*J*_C-F_ = 6.1 Hz), 122.8 (t, ^1^*J*_C-F_ = 243.4 Hz), 122.0 (t, ^3^*J*_C-F_ = 6.2 Hz), 121.5, 120.4, 117.8, 70.5, 41.3, 34.3 (t, ^2^*J*_C-F_ = 28.1 Hz), 21.5; IR (KBr): 3065, 2924, 1687, 1606, 1460, 1369, 1149, 1077, 764, 702, 665; HRMS (ESI) calcd. for C_18_H_16_F_2_O_2_ [M + H]^+^ 303.1197, found: 303.1193.

*3-(2-(3,4-Dimethylphenyl)-2,2-difluoroethyl)chroman-4-one* (**3ag**), 58.2 mg, 92% yield, light yellow solid; mp 84.5–84.9 °C; ^1^H NMR (500 MHz, CDCl_3_) δ 7.89 (dd, *J* = 7.9, 1.7 Hz, 1H), 7.47 (ddd, *J* = 8.8, 7.2, 1.8 Hz, 1H), 7.31 (s, 1H), 7.27 (d, *J* = 8.0 Hz, 1H), 7.19 (d, *J* = 7.8 Hz, 1H), 7.01 (ddd, *J* = 8.0, 7.3, 1.0 Hz, 1H), 6.97 (d, *J* = 8.4 Hz, 1H), 4.75 (dd, *J* = 11.5, 5.1 Hz, 1H), 4.25 (t, *J* = 11.7 Hz, 1H), 3.27–2.89 (m, 2H), 2.31 (s, 3H), 2.29 (s, 3H), 2.14–1.99 (m, 1H); ^19^F NMR (470 MHz, CDCl_3_) δ −88.88 (ddd, *J* = 245.1, 23.9, 8.1 Hz), −96.66 (dt, *J* = 245.1, 19.0 Hz); ^13^C NMR (125 MHz, CDCl_3_) δ 192.2, 161.7, 138.8, 137.1, 136.0, 134.0 (t, ^2^*J*_C-F_ = 26.2 Hz), 129.8, 127.5, 126.0 (t, ^3^*J*_C-F_ = 6.0 Hz), 122.9 (t, ^1^*J*_C-F_ = 242.8 Hz), 122.3 (t, ^3^*J*_C-F_ = 6.2 Hz), 121.5, 120.4, 117.8, 70.6, 41.3, 35.3 (t, ^2^*J*_C-F_ = 28.6 Hz), 19.9, 19.6; IR (KBr): 3051, 2925, 1688, 1607, 1467, 1393, 1138, 1028, 822, 767, 681; HRMS (ESI) calcd. for C_19_H_18_F_2_O_2_ [M + H]^+^ 317.1353, found: 317.1359.

*3-(2,2-Difluoro-2-(m-tolyl)ethyl)chroman-4-one* (**3ae**), 36.3 mg, 55% yield, light yellow oil; ^1^H NMR (500 MHz, CDCl_3_) δ 7.91 (dd, *J* = 7.9, 1.7 Hz, 1H), 7.49 (ddd, *J* = 8.5, 7.2, 1.8 Hz, 1H), 7.03 (ddd, *J* = 8.1, 7.3, 1.0 Hz, 1H), 7.00 (d, *J* = 8.4 Hz, 1H), 6.87 (s, 2H), 4.86 (dd, *J* = 11.3, 5.2 Hz, 1H), 4.34 (t, *J* = 11.6 Hz, 1H), 3.34 (dddd, *J* = 11.8, 8.5, 5.2, 2.7 Hz, 1H), 3.04 (dddd, *J* = 33.1, 15.9, 7.2, 2.7 Hz, 1H), 2.43 (t, *J* = 4.4 Hz, 6H), 2.27 (s, 3H), 2.16–2.01 (m, 1H); ^19^F NMR (470 MHz, CDCl_3_) δ −85.20– −86.00 (m), −88.96 (dd, *J* = 250.6, 28.9 Hz); ^13^C NMR (125 MHz, CDCl_3_) δ 192.5, 161.7, 139.0, 136.0, 136.0 (t, ^3^*J*_C-F_ = 3.6 Hz), 131.2, 130.9 (t, ^2^*J*_C-F_ = 23.6 Hz), 127.5, 125.4 (t, ^1^*J*_C-F_ = 244.3 Hz), 121.5, 120.5, 117.9, 70.8 (d, ^3^*J*_C-F_ = 4.7 Hz), 41.0, 33.7 (t, ^2^*J*_C-F_ = 26.7 Hz), 22.2 (t, ^3^*J*_C-F_ = 6.5 Hz), 20.7; IR (KBr): 3027, 2926, 1693, 1604, 1467, 1375, 1160, 1037, 860, 761; HRMS (ESI) calcd. for C_20_H_20_F_2_O_2_ [M + H]^+^ 331.1510, found: 331.1511.

*3-(2-(Benzo[d][1,3]dioxol-5-yl)-2,2-difluoroethyl)chroman-4-one* (**3aj**), 27.6 mg, 41% yield, light yellow solid; mp 46.3–46.5 °C; ^1^H NMR (500 MHz, CDCl_3_) δ 7.87 (dd, *J* = 7.9, 1.7 Hz, 1H), 7.47 (ddd, *J* = 8.6, 7.2, 1.7 Hz, 1H), 7.06–6.92 (m, 4H), 6.83 (d, *J* = 8.1 Hz, 1H), 5.99 (s, 2H), 4.72 (dd, *J* = 11.4, 5.0 Hz, 1H), 4.24 (t, *J* = 11.7 Hz, 1H), 3.17–2.93 (m, 2H), 2.11–1.98 (m, 1H); ^19^F NMR (470 MH**_Z_**, CDCl_3_) δ −87.57 (ddd, *J* = 244.5, 23.5, 8.1 Hz), −95.50 (ddd, *J* = 244.3, 21.7, 16.7 Hz); ^13^C NMR (125 MHz, CDCl_3_) δ 192.1, 161.6, 149.0, 148.0, 136.1, 130.4 (t, ^2^*J*_C-F_ = 26.6 Hz), 127.5, 122.6 (t, ^1^*J*_C-F_ = 243.1 Hz), 121.5, 120.4, 119.0 (t, ^3^*J*_C-F_ = 6.8 Hz), 117.8, 108.2, 105.7 (t, ^3^*J*_C-F_ = 6.2 Hz), 101.6, 70.5, 41.3, 34.3 (t, ^2^*J*_C-F_ = 28.5 Hz); IR (KBr): 3045, 2920, 1691, 1604, 1498, 1447, 1101, 1038, 815, 758, 664; HRMS (ESI) calcd. for C_18_H_14_F_2_O_4_ [M + H]^+^ 333.0938, found: 333.0934.

*3-(2,2-Difluoro-2-(4-fluorophenyl)ethyl)chroman-4-one* (**3ak**), 24.5 mg, 40% yield, white solid; mp 93.6–93.9 °C; ^1^H NMR (500 MHz, CDCl_3_) δ 7.88 (dd, *J* = 7.9, 1.7 Hz, 1H), 7.53 (dd, *J* = 8.8, 5.2 Hz, 2H), 7.48 (ddd, *J* = 8.6, 7.2, 1.8 Hz, 1H), 7.12 (t, *J* = 8.6 Hz, 2H), 7.06–6.99 (m, 1H), 6.97 (d, *J* = 8.4 Hz, 1H), 4.75 (dd, *J* = 11.3, 5.2 Hz, 1H), 4.26 (t, *J* = 11.6 Hz, 1H), 3.22–2.93 (m, 1H), 2.24–1.89 (m, 1H); ^19^F NMR (470 MHz, CDCl_3_) δ −88.92 (ddd, *J* = 247.0, 24.8, 7.1 Hz), −96.62 (ddd, *J* = 246.6, 22.5, 15.7 Hz), −110.5; ^13^C NMR (125 MHz, CDCl_3_) δ 192.0, 163.6 (d, ^1^*J*_C-F_ = 250.0 Hz), 161.6, 136.1, 132.6 (td, ^2^*J*_C-F_ = 26.8 Hz, ^4^*J*_C-F_ = 3.2 Hz), 127.5, 127.2 (dt, ^3^*J*_C-F_ = 6.3 Hz, ^3^*J*_C-F_ = 6.2 Hz), 122.9 (t, ^1^*J*_C-F_ = 243.3 Hz), 121.6, 120.3, 117.8, 115.8 (d, ^2^*J*_C-F_ = 22.0 Hz), 70.5, 41.2, 34.3 (t, ^2^*J*_C-F_ = 28.0 Hz); IR (KBr): 3069, 2922, 1696, 1609, 1515, 1472, 1237, 1164, 1099, 832, 754; HRMS (ESI) calcd. for C_17_H_13_F_3_O_2_ [M + H]^+^ 307.0946, found: 307.0945.

*3-(2-(3-Bromophenyl)-2,2-difluoroethyl)chroman-4-one* (**3an**), 19.1 mg, 26% yield, white solid; mp 60.1–60.4 °C; ^1^H NMR (500 MHz, CDCl_3_) δ 7.88 (dd, *J* = 8.0, 1.6 Hz, 1H), 7.69 (s, 1H), 7.59 (d, *J* = 8.0 Hz, 1H), 7.51–7.45 (m, 2H), 7.33 (t, *J* = 7.9 Hz, 1H), 7.03 (ddd, *J* = 8.0, 7.2, 1.0 Hz, 1H), 6.98 (d, *J* = 8.4 Hz, 1H), 4.77 (dd, *J* = 11.3, 5.2 Hz, 1H), 4.27 (t, *J* = 11.7 Hz, 1H), 3.21–2.94 (m, 2H), 2.17–1.97 (m, 1H); ^19^F NMR (470 MHz, CDCl_3_) δ −90.78 (ddd, *J* = 246.6, 26.2, 7.6 Hz), −97.89 (ddd, *J* = 246.6, 23.7, 14.5 Hz); ^13^C NMR (125 MHz, CDCl_3_) δ 191.9, 161.7, 138.7 (t, ^2^*J*_C-F_ = 26.9 Hz), 136.2, 133.3, 130.3, 128.2 (t, ^3^*J*_C-F_ = 6.4 Hz), 127.6, 123.6 (t, ^3^*J*_C-F_ = 6.0 Hz), 122.8, 121.8 (t, ^1^*J*_C-F_ = 243.9 Hz), 121.6, 120.3, 117.8, 70.5, 41.1, 34.3 (t, ^2^*J*_C-F_ = 27.5 Hz); IR (KBr): 3035, 2923, 1693, 1603, 1470, 1120, 1064, 1001, 794, 753, 689, 591; HRMS (ESI) calcd. for C_17_H_13_BrF_2_O_2_ [M + H]^+^ 367.0145, found: 367.0141.

*3-(2,2-Difluoro-2-(4-iodophenyl)ethyl)chroman-4-one* (**3ao**), 49.7 mg, 60% yield, light yellow solid; mp 116.0–116.5 °C; ^1^H NMR (500 MHz, CDCl_3_) δ 7.87 (dd, *J* = 8.2, 1.7 Hz, 1H), 7.78 (d, *J* = 8.5 Hz, 2H), 7.47 (ddd, *J* = 8.4, 7.2, 1.8 Hz, 1H), 7.27 (d, *J* = 8.3 Hz, 2H), 7.01 (ddd, *J* = 8.1, 7.2, 1.0 Hz, 1H), 6.96 (d, *J* = 8.4 Hz, 1H), 4.74 (dd, *J* = 11.4, 5.1 Hz, 1H), 4.25 (t, *J* = 11.6 Hz, 1H), 3.16–2.96 (m, 2H), 2.10–1.99 (m, 1H); ^19^F NMR (470 MHz, CDCl_3_) δ −90.34 (ddd, *J* = 246.9, 24.9, 7.7 Hz), −97.88 (ddd, *J* = 246.8, 22.8, 15.4 Hz); ^13^C NMR (125 MHz, CDCl_3_) δ 191.9, 161.6, 137.9, 136.2, 136.2 (t, ^2^*J*_C-F_ = 26.6 Hz), 127.6, 126.7 (t, ^3^*J*_C-F_ = 5.8 Hz), 122.4 (t, ^1^*J*_C-F_ = 243.6 Hz), 121.6, 120.3, 117.8, 96.6, 70.4, 41.1, 34.1 (t, ^2^*J*_C-F_ = 27.8 Hz); IR (KBr): 3009, 2905, 1689, 1595, 1466, 1112, 1021, 826, 763, 555; HRMS (ESI) calcd. for C_17_H_13_F_2_IO_2_ [M + H]^+^ 415.0007, found: 415.0011.

*3-(2,2-Difluoro-2-((4-methoxyphenyl)thio)ethyl)chroman-4-one* (**3aq**), 21.0 mg, 30% yield, white solid; mp 67.5–68.0 °C; ^1^H NMR (500MHz, CDCl_3_) δ 7.89 (dd, *J* = 7.9, 1.7 Hz, 1H), 7.53 (d, *J* = 8.8 Hz, 2H), 7.49 (ddd, *J* = 8.8, 7.2, 1.8 Hz, 1H), 7.03 (ddd, *J* = 8.0, 7.3, 1.0 Hz, 1H), 6.98 (d, *J* = 9.1 Hz, 1H), 6.91 (d, *J* = 8.9 Hz, 2H), 4.75 (dd, *J* = 11.4, 5.3 Hz, 1H), 4.22 (t, *J* = 11.8 Hz, 1H), 3.83 (s, 3H), 3.33–3.19 (m, 1H), 3.11–3.00 (m, 1H), 2.15–1.95 (m, 1H); ^19^F NMR (470 MHz, CDCl_3_) δ −69.65 (ddd, *J* = 207.3, 19.6, 8.6 Hz), −74.59 (dt, *J* = 207.2, 17.4 Hz); ^13^C NMR (125 MHz, CDCl_3_) δ 191.6, 161.6, 161.3, 138.2, 136.1, 129.4 (t, ^1^*J*_C-F_ = 278.2 Hz), 127.6, 121.6, 120.3, 117.8, 116.8, 114.8, 70.3, 55.4, 41.7, 33.8 (t, ^2^*J*_C-F_ = 24.6 Hz); IR (KBr): 3052, 2929, 1688, 1596, 1469, 1394, 1153, 1026, 960, 823, 761; HRMS (ESI) calcd. for C_18_H_16_F_2_O_3_S [M + H]^+^ 351.0866, found: 351.0865.

*2-((2,2-Difluoro-6-methoxythiochroman-4-yl)methoxy)benzaldehyde* (**3aq’**), 20.3 mg, 29% yield, white solid; mp 64.0–64.5 °C; ^1^H NMR (500MHz, CDCl_3_) δ 10.48 (s, 1H), 7.84 (dd, *J* = 7.7, 1.8 Hz, 1H), 7.54 (ddd, *J* = 9.1, 7.4, 1.8 Hz, 1H), 7.12 (d, *J* = 8.6 Hz, 1H), 7.06 (t, *J* = 7.5 Hz, 1H), 6.99 (d, *J* = 8.4 Hz, 1H), 6.91 (d, *J* = 2.7 Hz, 1H), 6.83 (dd, *J* = 8.6, 2.7 Hz, 1H), 4.46–4.31 (m, 2H), 3.78 (s, 3H), 3.61–3.56 (m, 1H), 2.81–2.72 (m, 1H), 2.66–2.58 (m, 1H); ^19^F NMR (470 MHz, CDCl_3_) δ −60.89 (ddd, *J* = 219.7, 22.1, 13.6 Hz), −64.51 (dt, *J* = 219.8, 12.3 Hz); ^13^C NMR (125 MHz, CDCl_3_) δ 189.2, 160.6, 158.4, 136.0, 134.5, 130.5 (t, ^1^*J*_C-F_ = 268.7 Hz) 128.8, 128.3 (t, ^3^*J*_C-F_ = 2.9 Hz), 125.0, 121.3, 120.8, 115.1, 113.7, 112.5, 68.2 (d, ^3^*J*_C-F_ = 3.4 Hz), 55.5, 39.0 (d, ^3^*J* = 5.6 Hz), 37.6 (t, ^2^*J*_C-F_ = 23.8 Hz); IR (KBr): 3067, 2926, 1739, 1678, 1599, 1568, 1496, 1074, 1022, 990, 890, 806, 760; HRMS (ESI) calcd. for C_18_H_16_F_2_O_3_S [M + H]^+^ 351.0866, found: 351.0863.

*3-(2,2-Difluorobutyl)chroman-4-one* (**3ar**), 6.3 mg, 13% yield, light yellow oil; ^1^H NMR (500 MHz, CDCl_3_) δ 7.90 (dd, *J* = 7.9, 1.8 Hz, 1H), 7.53–7.44 (m, 1H), 7.02 (ddd, *J* = 8.1, 7.2, 1.1 Hz, 1H), 6.98 (d, *J* = 8.4 Hz, 1H), 4.76 (dd, *J* = 11.4, 5.2 Hz, 1H), 4.24 (t, *J* = 11.7 Hz, 1H), 3.17 (dddd, *J* = 12.1, 8.9, 5.3, 3.0 Hz, 1H), 2.74 (dddd, *J* = 29.4, 15.4, 10.9, 3.0 Hz, 1H), 2.00–1.87 (m, 2H), 1.80 (dddd, *J* = 24.8, 15.8, 9.3, 6.9 Hz, 1H), 1.06 (t, *J* = 7.5 Hz, 3H); ^19^F NMR (470 MHz, CDCl_3_) δ −96.70–−98.13 (m), −100.51–−101.83 (m); ^13^C NMR (125 MHz, CDCl_3_) δ 192.6, 161.7, 136.0, 127.5, 125.0 (t, ^1^*J*_C-F_ = 242.0 Hz), 121.5, 120.4, 117.8, 70.7, 40.9, 31.3 (t, ^2^*J*_C-F_ = 25.4 Hz), 30.5 (t, ^2^*J*_C-F_ = 26.0 Hz), 6.6 (t, ^3^*J*_C-F_ = 5.7 Hz); IR (KBr): 3041, 2928, 1694, 1609, 1468, 1370, 1137, 1040, 764, 673; HRMS (ESI) calcd. for C_13_H_14_F_2_O_2_ [M + H]^+^ 241.1040, found: 241.1037.

*3-(2,2-Difluoro-2-(4-methoxyphenyl)ethyl)-7-methoxychroman-4-one* (**3bb**), 36.2 mg, 52% yield, white solid; mp 105.9–106.1 °C; ^1^H NMR (500 MHz, CDCl_3_) δ 7.81 (d, *J* = 8.8 Hz, 1H), 7.46 (d, *J* = 8.8 Hz, 2H), 6.93 (d, *J* = 8.7 Hz, 2H), 6.57 (dd, *J* = 8.6, 2.6 Hz, 1H), 6.39 (d, *J* = 2.3 Hz, 1H), 4.70 (dd, *J* = 11.2, 5.2 Hz, 1H), 4.22 (t, *J* = 11.5 Hz, 1H), 3.82 (s, 6H), 3.32–2.85 (m, 2H), 2.31–1.82 (m, 1H); ^19^F NMR (470 MHz, CDCl_3_) δ −87.41 (ddd, *J* = 245.4, 23.5, 8.1 Hz), −95.84 (dt, *J* = 248.5, 20.6 Hz); ^13^C NMR (125 MHz, CDCl_3_) δ 190.8, 166.0, 163.6, 160.8, 129.2, 128.8 (t, ^2^*J*_C-F_ = 26.8 Hz), 126.4 (t, ^3^*J*_C-F_ = 6.1 Hz), 123.0 (t, ^1^*J*_C-F_ = 242.2 Hz), 114.2, 113.9, 110.1, 100.6, 70.9, 55.6, 55.4, 40.9, 34.3 (t, ^2^*J*_C-F_ = 28.5 Hz); IR (KBr): 3021, 2957, 1690, 1614, 1511, 1460, 1374, 1614, 1167, 1026, 879, 830; HRMS (ESI) calcd. For C_19_H_18_F_2_O_4_ [M + H]^+^ 349.1251, found: 349.1248.

*3-(2,2-Difluoro-2-(4-methoxyphenyl)ethyl)-6-methylchroman-4-one* (**3cb**), 23.3 mg, 35% yield, white solid; mp 69.6–70.1 °C; ^1^H NMR (500 MHz, CDCl_3_) δ 7.66 (d, *J* = 1.9 Hz, 1H), 7.46 (d, *J* = 8.8 Hz, 2H), 7.28 (dd, *J* = 8.9, 2.3 Hz, 1H), 6.94 (d, *J* = 8.8 Hz, 2H), 6.86 (d, *J* = 8.5 Hz, 1H), 4.69 (dd, *J* = 11.2, 5.1 Hz, 1H), 4.21 (t, *J* = 11.5 Hz, 1H), 3.83 (s, 3H), 3.21–2.94 (m, 2H), 2.30 (s, 3H), 2.12–2.00 (m, 1H); ^19^F NMR (470 MHz, CDCl_3_) δ −87.69 (ddd, *J* = 245.5, 23.3, 8.2 Hz), −95.74 (ddd, *J* = 245.5, 21.5, 16.7 Hz); ^13^C NMR (125 MHz, CDCl_3_) δ 192.4, 160.8, 159.7, 137.1, 130.9, 128.8 (t, ^2^*J*_C-F_ = 26.8 Hz), 127.0, 126.4 (t, ^3^*J*_C-F_ = 6.1 Hz), 122.9 (t, ^1^*J*_C-F_ = 242.2 Hz), 120.0, 117.6, 113.9, 70.5, 55.4, 41.4, 34.3 (t, ^2^*J*_C-F_ = 28.8 Hz), 20.4; IR (KBr): 3027, 2916, 1697, 1617, 1501, 1424, 1302, 1169, 1028, 823, 735; HRMS (ESI) calcd. for C_19_H_18_F_2_O_3_ [M + H]^+^ 333.1302, found: 333.1306.

*3-(2,2-Difluoro-2-(4-methoxyphenyl)ethyl)-7-methylchroman-4-one* (**3db**), 33.2 mg, 50% yield, white solid; mp 64.4–64.7 °C; ^1^H NMR (500 MHz, CDCl_3_) δ 7.76 (d, *J* = 8.0 Hz, 1H), 7.46 (d, *J* = 8.8 Hz, 2H), 6.93 (d, *J* = 8.8 Hz, 2H), 6.82 (ddd, *J* = 8.0, 1.5, 0.5 Hz, 1H), 6.76 (s, 1H), 4.70 (dd, *J* = 11.4, 5.2 Hz, 1H), 4.21 (t, *J* = 11.6 Hz, 1H), 3.82 (s, 3H), 3.20–2.88 (m, 2H), 2.35 (s, 3H), 2.05 (dddd, *J* = 22.4, 15.9, 9.7, 8.7 Hz, 1H); ^19^F NMR (470 MHz, CDCl_3_) δ −87.58 (ddd, J = 245.5, 23.2, 8.1 Hz), −95.75 (ddd, *J* = 245.4, 21.5, 16.8 Hz); ^13^C NMR (125 MHz, CDCl_3_) δ 191.9, 161.7, 160.8, 147.6, 128.8 (t, ^2^*J*_C-F_ = 26.9 Hz), 127.4, 126.4 (t, ^3^*J*_C-F_ = 6.1 Hz), 122.9 (t, ^1^*J*_C-F_ = 242.0 Hz), 122.9, 118.1, 117.8, 113.9, 70.5, 55.4, 41.2, 34.2 (t, ^2^*J*_C-F_ = 28.6 Hz), 21.9; IR (KBr): 3019, 2934, 1684, 1612, 1513, 1319, 1167, 974, 827; HRMS (ESI) calcd. for C_19_H_18_F_2_O_3_ [M + H]^+^ 333.1302, found: 333.1300.

*6,8-Di-tert-butyl-3-(2,2-difluoro-2-(4-methoxyphenyl)ethyl)chroman-4-one* (**3eb**), 38.8 mg, 45% yield, light yellow oil; ^1^H NMR (500 MHz, CDCl_3_) δ 7.79 (d, *J* = 2.5 Hz, 1H), 7.54 (d, *J* = 2.6 Hz, 1H), 7.48 (d, *J* = 8.8 Hz, 2H), 6.95 (d, *J* = 8.8 Hz, 2H), 4.79 (s, 1H), 4.22 (t, *J* = 11.5 Hz, 1H), 3.83 (s, 3H), 3.18–2.97 (m, 2H), 2.18–2.00 (m, 1H), 1.40 (s, 9H), 1.30 (s, 9H); ^19^F NMR (470 MHz, CDCl_3_) δ −87.06 (ddd, *J* = 245.5, 23.6, 7.9 Hz), −95.98 (ddd, *J* = 245.5, 21.8, 16.8 Hz); ^13^C NMR (125 MHz, CDCl_3_) δ 193.2, 160.8, 158.8, 143.4, 138.3, 130.8, 128.9 (t, ^2^*J*_C-F_ = 26.8 Hz), 126.5 (t, ^3^*J*_C-F_ = 6.1 Hz), 123.0 (t, ^1^*J*_C-F_ = 242.4 Hz), 121.5, 120.4, 113.9, 70.0, 55.4, 41.3, 35.1, 34.5, 34.4 (t, ^2^*J*_C-F_ = 28.4 Hz), 31.3, 29.7; IR (KBr): 3006, 2920, 1682, 1613, 1471, 1364, 1171, 1023, 846, 758, 678; HRMS (ESI) calcd. for C_26_H_32_F_2_O_3_ [M + H]^+^ 431.2398, found: 431.2395.

*3-(2,2-Difluoro-2-(4-methoxyphenyl)ethyl)-6-fluorochroman-4-one* (**3fb**), 34.3 mg, 51% yield, white solid; mp 112.4–112.8 °C; ^1^H NMR (500 MHz, CDCl_3_) δ 7.52 (dd, *J* = 8.3, 3.2 Hz, 1H), 7.46 (d, *J* = 8.8 Hz, 2H), 7.20 (ddd, *J* = 9.1, 7.7, 3.2 Hz, 1H), 6.95 (dd, *J* = 8.6, 5.0 Hz, 3H), 4.73 (dd, *J* = 11.7, 5.2 Hz, 1H), 4.23 (t, *J* = 11.8 Hz, 1H), 3.83 (s, 3H), 3.15–3.00 (m, 2H), 2.13–2.00 (m, 1H); ^19^F NMR (470 MHz, CDCl_3_) δ −87.90 (ddd, *J* = 245.6, 23.8, 8.2 Hz), −95.81 (ddd, *J* = 245.5, 21.9, 16.3 Hz), -121.27 (td, *J* = 8.1, 4.3 Hz); ^13^C NMR (125 MHz, CDCl_3_) δ 191.5, 160.9, 158.2, 157.9 (d, ^4^*J*_C-F_ = 1.7 Hz), 156.3, 128.6 (t, ^2^*J*_C-F_ = 26.8 Hz), 126.4 (dd, ^3^*J*_C-F_ = 6.4Hz, *J* = 5.6Hz), 123.6 (d, ^2^*J*_C-F_ = 24.6 Hz), 122.7 (t, ^1^*J*_C-F_ = 242.1 Hz), 120.7 (d, ^3^*J*_C-F_ = 6.6 Hz), 119.5 (d, ^3^*J*_C-F_ = 7.3 Hz), 112.4 (d, ^2^*J*_C-F_ = 23.4 Hz), 70.7, 55.6, 41.2, 34.2 (dd, ^2^*J*_C-F_ = 29.2, 28.0 Hz); IR (KBr): 3061, 2939, 1686, 1619, 1487, 1441, 1380, 1122, 1020, 897, 822, 744; HRMS (ESI) calcd. for C_18_H_15_F_3_O_3_ [M + H]^+^ 337.1052, found: 337.1049.

*6-Chloro-3-[2,2-difluoro-2-(4-methoxy-phenyl)-ethyl]-chroman-4-one* (**3gb**), 39.5 mg, 56% yield, white solid; mp 116.1–116.5 °C; ^1^H NMR (500 MHz, CDCl_3_) δ 7.83 (d, *J* = 2.7 Hz, 1H), 7.45 (d, *J* = 8.8 Hz, 2H), 7.41 (dd, *J* = 8.8, 2.7 Hz, 1H), 6.93 (dd, *J* = 8.8, 5.7 Hz, 3H), 4.74 (dd, *J* = 11.6, 5.2 Hz, 1H), 4.23 (t, *J* = 11.7 Hz, 1H), 3.83 (s, 3H), 3.15–3.00 (m, 2H), 2.12–1.98 (m, 1H); ^19^F NMR (470 MHz, CDCl_3_) δ −87.93 (ddd, *J* = 245.8, 24.0, 8.1 Hz), −95.83 (ddd, *J* = 245.5, 22.0, 16.1 Hz); ^13^C NMR (125 MHz, CDCl_3_) δ 191.1, 160.9, 160.1, 135.9, 128.7 (t, ^2^*J*_C-F_= 26.9 Hz), 127.0, 126.8, 126.4 (t, ^3^*J*_C-F_ = 6.1 Hz), 122.7 (t, ^1^*J*_C-F_ = 242.3 Hz), 121.1, 119.6, 114.0, 70.6, 55.4, 41.2, 34.2 (t, ^2^*J*_C-F_ = 28.9 Hz); IR (KBr): 3087, 2921, 1698, 1610, 1571, 1515, 1472, 1381, 1104, 1022, 889, 826, 776, 426; HRMS (ESI) calcd. for C_18_H_15_ClF_2_O_3_ [M + H]^+^ 353.0756, found: 353.0753.

*6-Bromo-3-(2,2-difluoro-2-(4-methoxyphenyl)ethyl)chroman-4-one* (**3hb**), 43.7 mg, 55% yield, white solid; mp 125.4–125.7 °C; ^1^H NMR (500 MHz, CDCl_3_) δ 7.97 (d, *J* = 2.5 Hz, 1H), 7.54 (dd, *J* = 8.8, 2.6 Hz, 1H), 7.45 (d, *J* = 8.8 Hz, 2H), 6.94 (d, *J* = 8.8 Hz, 2H), 6.87 (d, *J* = 8.8 Hz, 1H), 4.75 (dd, *J* = 11.5, 5.1 Hz, 1H), 4.23 (t, *J* = 11.7 Hz, 1H), 3.83 (s, 3H), 3.18–2.96 (m, 2H), 2.19–1.91 (m, 1H); ^19^F NMR (470 MHz, CDCl_3_) δ −87.93 (ddd, *J* = 245.7, 24.0, 8.0 Hz), −95.83 (ddd, *J* = 245.5, 22.0, 16.0 Hz); ^13^C NMR (125 MHz, CDCl_3_) δ 191.0, 160.9, 160.5, 138.7, 129.9, 128.6 (t, ^2^*J*_C-F_= 26.9 Hz), 126.4 (t, ^3^*J*_C-F_ = 5.9 Hz), 122.7 (t, ^1^*J*_C-F_ = 242.2 Hz), 121.6, 119.9, 114.1, 114.0, 70.6, 55.4, 41.1, 34.2 (t, ^2^*J*_C-F_ = 28.7 Hz); IR (KBr): 3083, 2908, 1697, 1608, 1515, 1470, 1103, 1023, 889, 824, 777, 675; HRMS (ESI) calcd. for C_18_H_15_BrF_2_O_3_ [M + H]^+^ 397.0251, found: 397.0253.

*7-Chloro-3-(2,2-difluoro-2-(4-methoxyphenyl)ethyl)chroman-4-one* (**3ib**), 45.2 mg, 64% yield, white solid; mp 116.3–116.6 °C; ^1^H NMR (500 MHz, CDCl_3_) δ 7.80 (d, *J* = 8.9 Hz, 1H), 7.45 (d, *J* = 8.7 Hz, 2H), 7.00–6.97 (m, 2H), 6.94 (d, *J* = 8.7 Hz, 2H), 4.75 (dd, *J* = 11.7, 5.2 Hz, 1H), 4.24 (t, *J* = 11.8 Hz, 1H), 3.82 (s, 3H), 3.17–2.97 (m, 2H), 2.15–1.95 (m, 1H); ^19^F NMR (470 MHz, CDCl_3_) δ −87.90 (ddd, *J* = 245.6, 23.9, 8.2 Hz), −95.75 (ddd, *J* = 245.6, 21.9, 16.2 Hz); ^13^C NMR (125MHz, CDCl_3_) δ 191.2, 162.0, 160.9, 141.9, 128.8, 128.6 (t, ^2^*J*_C-F_ = 26.8 Hz), 126.4 (t, ^3^*J*_C-F_ = 6.1 Hz), 122.8 (t, ^1^*J*_C-F_ = 242.0 Hz), 122.3, 118.9, 118.0, 114.0, 70.8, 55.4, 41.2, 34.2 (t, ^2^*J*_C-F_ = 28.6 Hz); IR (KBr): 3012, 2949, 1674, 1605, 1516, 1468, 1379, 1069, 1025, 934, 834, 806, 705, 579, 448; HRMS (ESI) calcd. for C_18_H_15_ClF_2_O_3_ [M + H]^+^ 353.0756, found: 353.0755.

*8-Chloro-3-(2,2-difluoro-2-(4-methoxyphenyl)ethyl)chroman-4-one* (**3jb**), 24.7 mg, 35% yield, light yellow solid; mp 76.1–76.8 °C; ^1^H NMR (500 MHz, CDCl_3_) δ 7.80 (dd, *J* = 8.0, 1.6 Hz, 1H), 7.56 (dd, *J* = 8.0, 1.6 Hz, 1H), 7.46 (d, *J* = 9.2 Hz, 2H), 7.00–6.91 (m, 3H), 4.89 (dd, *J* = 11.2, 5.2 Hz, 1H), 4.31 (t, *J* = 11.6 Hz, 1H), 3.83 (s, 3H), 3.23–2.95 (m, 2H), 2.15–2.01 (m, 1H); ^19^F NMR (470 MHz, CDCl_3_) δ −87.87 (ddd, *J* = 245.9, 23.0, 8.2 Hz), −95.55 (ddd, *J* = 246.0, 21.4, 16.8 Hz); ^13^C NMR (125 MHz, CDCl_3_) δ 191.3, 160.9, 157.1, 136.1, 128.5 (t, ^2^*J*_C-F_ = 26.8 Hz), 126.4 (t, ^3^*J*_C-F_ = 6.1 Hz), 126.1, 122.7 (t, ^1^*J*_C-F_ = 242.4 Hz), 122.6, 121.6, 121.6, 114.0, 71.1, 55.4, 41.2, 34.1 (t, ^2^*J*_C-F_ = 28.9 Hz); IR (KBr): 3074, 2924, 1682, 1608, 1450, 1515, 1374, 1115, 1023, 835, 767, 730, 595, 432; HRMS (ESI) calcd. for C_18_H_15_ClF_2_O_3_ [M + H]^+^ 353.0756, found: 353.0759.

*3-(2,2-Cifluoro-2-(4-methoxyphenyl)ethyl)-6-iodochroman-4-one* (**3kb**), 35.5 mg, 40% yield, white solid; mp 130.1–131.1 °C; ^1^H NMR (500 MHz, CDCl_3_) δ 8.15 (d, *J* = 2.2 Hz, 1H), 7.70 (dd, *J* = 8.7, 2.3 Hz, 1H), 7.44 (d, *J* = 8.7 Hz, 2H), 6.93 (d, *J* = 8.7 Hz, 2H), 6.74 (d, *J* = 8.7 Hz, 1H), 4.74 (dd, *J* = 11.5, 5.1 Hz, 1H), 4.22 (t, *J =* 11.7 Hz, 1H), 3.82 (s, 3H), 3.14–2.99 (m, 2H), 2.10–1.99 (m, 2H); ^19^F NMR (470 MHz, CDCl_3_) δ −87.86 (ddd, *J* = 245.8, 24.0, 7.8 Hz), −95.71 (ddd, *J* = 245.5, 21.8, 16.2 Hz); ^13^C NMR (125 MHz, CDCl_3_) δ 190.8, 161.2, 160.9, 144.3, 136.1, 128.6 (t, ^2^*J*_C-F_ = 26.7 Hz), 126.4 (t, ^3^*J*_C-F_ = 6.1 Hz), 122.7 (t, ^1^*J*_C-F_ = 242.3 Hz), 122.2, 120.2, 114.0, 83.8, 70.5, 55.4, 41.0, 34.2 (t, ^2^*J*_C-F_ = 28.7 Hz); IR (KBr): 3078, 2902, 1692, 1592, 1514, 1101, 1027, 893, 823, 781, 571; HRMS (ESI) calcd. for C_18_H_15_F_2_IO_3_ [M + H]^+^ 445.0122, found: 445.0124.

*6,8-Dibromo-3-(2,2-difluoro-2-(4-methoxyphenyl)ethyl)chroman-4-one* (**3lb**), 17.1 mg, 18% yield, white solid; mp 125.2–125.4 °C; ^1^H NMR (500 MHz, CDCl_3_) δ 7.94 (d, *J* = 2.4 Hz, 1H), 7.83 (d, *J* = 2.4 Hz, 1H), 7.44 (d, *J* = 8.8 Hz, 2H), 6.94 (d, *J* = 8.7 Hz, 2H), 4.90 (dd, *J* = 11.5, 5.4 Hz, 1H), 4.30 (t, *J* = 12.0 Hz, 1H), 3.83 (s, 3H), 3.21–2.97 (m, 2H), 2.20–1.96 (m, 1H); ^19^F NMR (470 MHz, CDCl_3_) δ −87.96 (ddd, *J* = 25.8, 23.4, 7.7 Hz), −95.58 (ddd, *J* = 245.8, 21.4, 16.2 Hz); ^13^C NMR (125 MHz, CDCl_3_) δ 190.2, 160.9, 157.1, 141.1, 129.4, 128.4 (t, ^2^*J*_C-F_ = 26.6 Hz), 126.4 (t, ^3^*J* _C-F_ = 6.0 Hz), 122.6 (t, ^1^*J*_C-F_ = 241.7 Hz), 122.2, 114.0, 112.6, 71.2, 55.4, 40.8, 34.1 (t, ^2^*J*_C-F_ = 28.9 Hz); IR (KBr): 3063, 2939, 1685, 1614, 1515, 1438, 1114, 1022, 894, 837, 806, 767, 576; HRMS (ESI) calca. For C_18_H_14_Br_2_F_2_O_3_ [M + H]^+^ 474.9356, found: 474.9353.

*3-(2,2-Difluoro-2-(4-methoxyphenyl)ethyl)-3-methylchroman-4-one* (**3mb**), 27.3 mg, 41% yield, light yellow oil; ^1^H NMR (500 MHz, CDCl_3_) δ 7.89 (dd, *J* = 7.9, 1.8 Hz, 1H), 7.48 (ddd, *J* = 8.4, 7.2, 1.8 Hz, 1H), 7.41 (d, *J* = 8.9 Hz, 2H), 7.03 (ddd, *J* = 8.0, 7.2, 1.0 Hz, 1H), 6.98 (dd, *J* = 8.4, 0.7 Hz, 1H), 6.90 (d, *J* = 8.8 Hz, 2H), 4.58 (d, *J* = 11.6 Hz, 1H), 4.35 (d, *J* = 11.7 Hz, 1H), 3.82 (s, 3H), 2.74 (ddd, *J* = 29.9, 15.7, 7.9 Hz, 1H), 2.42 (ddd, *J* = 26.9, 15.7, 11.5 Hz, 1H), 1.32 (s, 3H); ^19^F NMR (470 MHz, CDCl_3_) δ −89.01 (ddd, *J* = 243.5, 29.9, 11.5 Hz), −90.24 (ddd, *J* = 243.3, 26.7, 7.9 Hz); ^13^C NMR (125 MHz, CDCl_3_) δ 195.2, 161.0, 160.6, 135.7, 130.0 (t, ^2^*J*_C-F_ = 26.7 Hz), 128.1, 126.3 (t, ^3^*J*_C-F_ = 6.3 Hz), 122.9 (t, ^1^*J*_C-F_ =244.2 Hz), 121.6, 119.3, 117.7, 113.7, 74.5, 55.3, 44.1, 41.7 (t, ^2^*J*_C-F_ = 27.6 Hz), 19.8; IR (KBr): 3072, 2932, 1690, 1611, 1515, 1467, 1363, 1111, 1031, 829, 764; HRMS (ESI) calcd. for C_19_H_18_F_2_O_3_ [M + H]^+^ 333.1302, found: 333.1306.

*2-(2,2-Difluoro-2-(p-tolyl)ethyl)-2,3-dihydro-1H-inden-1-one* (**3na**), 21.1mg, 37% yield, white solid; mp 122.8–123.3 °C; ^1^H NMR (500 MHz, CDCl_3_) δ 7.75 (d, J = 7.7 Hz, 1H), 7.43 (d, J = 8.1 Hz, 2H), 7.38 (d, *J* = 7.1 Hz, 1H), 7.24 (d, *J* = 7.9 Hz, 2H), 3.44 (dd, *J* = 17.5, 8.1 Hz, 1H), 3.08–2.92 (m, 2H), 2.90–2.81 (m, 1H), 2.39 (s, 3H), 2.14 (dddd, *J* = 20.7, 14.9, 11.5, 8.0 Hz, 1H); ^19^F NMR (470 MHz, CDCl_3_) δ −90.63 (ddd, *J* = 244.7, 21.8, 7.9 Hz), −96.99 (ddd, *J* = 244.6, 20.3, 16.9 Hz); ^13^C NMR (125 MHz, CDCl_3_) δ 206.6, 153.7, 140.0, 136.0, 135.0, 134.1 (t, ^2^*J*_C-F_ = 26.4 Hz), 129.2, 127.5, 126.5, 124.8 (t, ^3^*J*_C-F_ = 6.2 Hz), 124.0, 123.2 (t,^1^*J*_C-F_ = 242.7 Hz), 42.9, 40.2 (t, ^2^*J*_C-F_ = 27.8 Hz), 33.9, 21.3; IR (KBr): 3054, 2924, 1711, 1597, 1460, 1080, 1021, 849, 811, 760; HRMS (ESI) calcd. for C_18_H_16_F_2_O [M + H]^+^ 287.1247, found: 287.1243.

## Figures and Tables

**Table 1:** Optimization of Reaction Conditions ^a^.

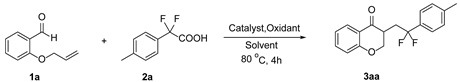				
Entry	Catalyst (mol%)	Oxidant (equiv.)	Solvent	Yield (%) ^b^
1	AgNO_3_ (20)	K_2_S_2_O_8_ (2.0)	CH_3_CN/H_2_O (1:1)	56
2	AgNO_3_ (20)	K_2_S_2_O_8_ (2.0)	DMSO/H_2_O (1:1)	45
3	AgNO_3_ (20)	K_2_S_2_O_8_ (2.0)	EtOH/H_2_O (1:1)	5
4	AgNO_3_ (20)	K_2_S_2_O_8_ (2.0)	H_2_O	10
5	AgNO_3_ (20)	K_2_S_2_O_8_ (2.0)	CH_3_CN	N.R.
**6**	**AgNO_3_ (20)**	**K_2_S_2_O_8_ (2.0)**	**CH_3_CN/H_2_O (1:3)**	**73**
7	AgNO_3_ (20)	Na_2_S_2_O_8_ (2.0)	CH_3_CN/H_2_O (1:3)	66
8	AgNO_3_ (20)	(NH_4_)_2_S_2_O_8_ (2.0)	CH_3_CN/H_2_O (1:3)	63
9	AgNO_3_ (20)	Selectfluor (2.0)	CH_3_CN/H_2_O (1:3)	0
10	\	K_2_S_2_O_8_ (2.0)	CH_3_CN/H_2_O (1:3)	49
11	AgNO_3_ (20)	\	CH_3_CN/H_2_O (1:3)	0
12	AgNO_3_ (30)	K_2_S_2_O_8_ (2.0)	CH_3_CN/H_2_O (1:3)	67
13	AgNO_3_ (10)	K_2_S_2_O_8_ (2.0)	CH_3_CN/H_2_O (1:3)	63
14 ^c^	AgNO_3_ (20)	K_2_S_2_O_8_ (2.0)	CH_3_CN/H_2_O (1:3)	50
15 ^d^	AgNO_3_ (20)	K_2_S_2_O_8_ (2.0)	CH_3_CN/H_2_O (1:3)	53
16 ^e^	AgNO_3_ (20)	K_2_S_2_O_8_ (2.0)	CH_3_CN/H_2_O (1:3)	68
17	AgNO_3_ (20)	K_2_S_2_O_8_ (1.75)	CH_3_CN/H_2_O (1:3)	70
18	AgNO_3_ (20)	K_2_S_2_O_8_ (2.25)	CH_3_CN/H_2_O (1:3)	64
19	AgOAc (20)	K_2_S_2_O_8_ (2.0)	CH_3_CN/H_2_O (1:3)	65
20	Ag_2_O (20)	K_2_S_2_O_8_ (2.0)	CH_3_CN/H_2_O (1:3)	68
21	Ag_2_CO_3_ (20)	K_2_S_2_O_8_ (2.0)	CH_3_CN/H_2_O (1:3)	65
22 ^f^	\	(NH_4_)_2_S_2_O_8_ (2.0)	DMSO	32

^a^ Reaction conditions: **1a** (0.2 mmol, 1.0 equiv.), **2a** (0.3 mmol, 1.5 equiv), catalyst (20 mol%) and oxidant (0.4 mmol) in 2.0 mL solvent at 80 °C for 4 h under N_2_ atmosphere. ^b^ Yields determined by ^19^F NMR analysis with PhCF_3_ as the internal standard. ^c^ **1a**:**2a** = 1:1. ^d^ **1a**:**2a** = 1.5:1. ^e^ **1a**:**2a** = 1:2. ^f^ **1a** (0.2 mmol), **2** (0.3 mmol), (NH_4_)_2_S_2_O_8_ (0.4mmol) and DMSO (2.0 mL) at 40 °C for 6 h under N_2_ atmosphere.

**Table 2:** Scope of α,α-difluoroarylacetic acids ^a,b^.

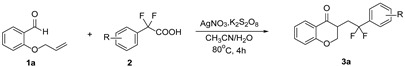
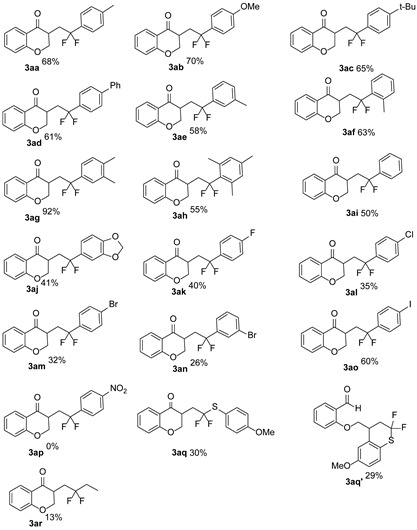

^a^ Reaction conditions: **1a** (0.2 mmol), **2a** (0.3 mmol), AgNO_3_ (20 mol%) and K_2_S_2_O_8_ (0.4 mmol) in 2.0 mL CH_3_CN/H_2_O (*v*/*v* = 1:3) solution at 80 °C for 4 h under N_2_ atmosphere. ^b^ Isolated yields.

**Table 3:** Scope of 2-allyloxybenzaldehydes ^a,b^.

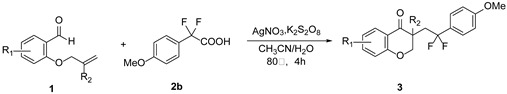
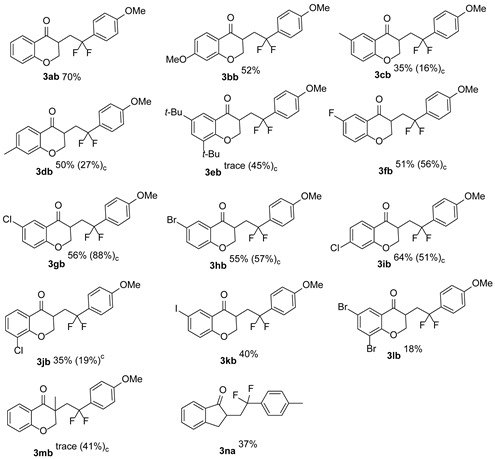

^a^ Reaction conditions: **1a** (0.2 mmol), **2** (0.3 mmol), AgNO_3_ (20 mol%) and K_2_S_2_O_8_ (0.4 mmol) in 2.0 mL CH_3_CN:H_2_O = 1:3 solution at 80 °C for 4 h under N_2_ atmosphere. ^b^ Isolated yields. ^c^ **1a** (0.2 mmol), **2** (0.3 mmol), (NH_4_)_2_S_2_O_8_ (0.4mmol) in 2.0 mL DMSO at 40 °C for 6 h under N_2_ atmosphere.

## Data Availability

The data presented in this study are contained in the article tables and Supplementary Materials.

## Supplementary Materials

The following supporting information can be downloaded at: https://www.mdpi.com/article/10.3390/molecules28083578/s1, [34,42,44,45,46], Table S1: Screening the reaction time; Table S2: Screening the reaction temp; Figure S1: Crystal structure of 3aq′; Table S3: Sample and crystal data for 3aq′; Table S4: Bond lengths (Å) for 3aq′; Table S5: Bond angles (°) for 3aq′; Figure S2: Copies of ^1^H NMR, ^19^F NMR and ^13^C NMR spectra of all compounds.

## Sample Availability

Samples of the compounds 3 are available from the authors.
