# Supplementary material for: Silver-Promoted Radical Cascade Aryldifluoromethylation/Cyclization of 2-Allyloxybenzaldehydes for the Synthesis of 3-Aryldifluoromethyl-Containing Chroman-4-one Derivatives
Source: Molecules. 2023 Apr 19;28(8):3578. doi: 10.3390/molecules28083578 (PMC10142801; doi:10.3390/molecules28083578)
Supplement: Supplementary file 1 [file molecules-28-03578-s001.zip › molecules-2331898-supplementary.pdf]

Suppporting information

Silver-Promoted Radical Cascade Aryldifluoromethylation/Cyclization of 2-Allyloxybenzaldehydes for the Synthesis of 3-Aryldifluoromethyl-Containing Chroman-4-One Derivatives

Qianqian Sun,[a] Hongxiao Li,[a] Xingyu Chen,[a] Jian Hao,*[a] Hongmei Deng,[c] Haizhen Jiang*[a,b]

[a] Department of Chemistry, College of Sciences, Shanghai University, Shanghai, 200444, PR China

[b] Key Laboratory of Organofluorine Chemistry, Shanghai Institute of Organic Chemistry, Chinese Academy of Sciences, Shanghai, 200032, PR China

[c] Laboratory for Microstructures, Shanghai University, Shanghai, 200444, PR China

Table of Contents

1. General information..1

2. Preparation of substrates..1

3. Optimization of the reaction conditions...3

4. General procedure for the synthesis of compounds **3**...3

5. Data for compounds **3**...5-15

6. Single crystal X-ray analysis of **3aq′**...16-18

7. Reference 19

8. Copies of ^{1}H NMR, ^{19}F NMR and ^{13}C NMR spectra...20-64

1. General information

^{1}H NMR spectra were recorded on a 500 MHz.^{19}F NMR were recorded on a 470 MHz spectrometer. ^{13}C NMR spectra were recorded on a 125 MHz spectrometer. ^{1}H NMR and ^{13}C NMR chemical shifts were determined relative to internal standard TMS at δ 0.0 and ^{19}F NMR chemical shifts were determined relative to $CFCl_3$ as inter standard. Chemical shifts (δ) are reported in ppm, and coupling constants (J) are in Hertz (Hz). The following abbreviations were used to explain the multiplicities: s = singlet, d = doublet, t = triplet, q = quartet, m = multiplet, br = broad. NMR yield was determined by ^{19}F NMR using trifluorotolueneas an internal standard before working up the reaction. Infrared spectra (IR) were recorded on AVATAR 370 FT-IR spectrometer, absorbance frequencies are given at maximum of intensity in cm^{-1}. High-resolution mass spectra (HRMS) were measured with JEOL JMX-SX 102A spectrometer (FAB) and electrospray (ESI). Silica gel (200−400mesh) was used for flash column chromatography.

2. Preparation of substrates

2.1 General experimental procedures for the synthesis of 2-allyloxyarylaldehydes 1a - 1n[1]

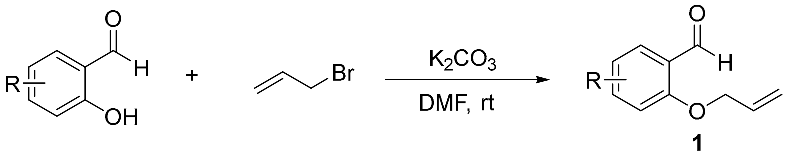

2-Allyloxyarylaldehyde **1** was prepared according the reported procedures. In a 50 mL round-bottomed flask with a stir bar, salicylaldehyde derivative (5 mmol, 1.0 equiv.) was dissolved in 15 mL DMF and kept stirring at room temperature. To this stirring solution was added potassium carbonate (6 mmol, 1.2 equiv.) followed by the dropwise addition of allyl bromide (6 mmol, 1.2 equiv.). The reaction mixture was then stirred for 15 h at room temperature. Water (50 mL) was then added and the mixture was extracted with dichloromethane (3 × 25 mL). The combined organic extracts were washed with brine (50 mL), dried over $MgSO_4$ and concentrated under reduced pressure. The crude product was then purified by flash column chromatography to afford the 2-allyloxybenzaldehyde derivatives. (*Org. Chem. Front.* **2018**, *5*, 2925.)

2.2 General experimental procedures for the synthesis of 2-allylbenzaldehyde 1o[2]

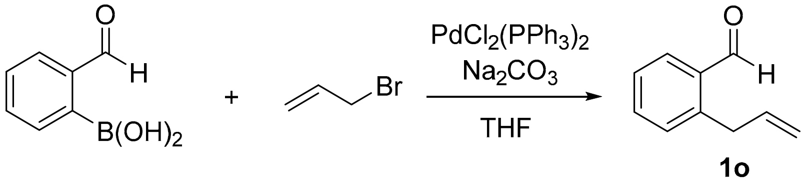

A 100 mL round-bottom flask was equipped with a rubber septum and magnetic stir bar and was charged with a solution of 2-formylphenylboronic acid (950 mg, 5 mmol) and allyl bromide (0.5 mL, 6 mmol) in THF (25 mL). Then $PdCl_2(PPh_3)_2$ (88 mg, 0.125 mmol) and aq. Na_2CO_3 (1M, 10 mmol) solution was added. The reaction mixture was heated at reflux for 3-4 h. The reaction mixture was quenched with H_2O and extracted with CH_2Cl_2 three times. The combined organic layers were washed with H_2O, dried over $MgSO_4$, and concentrated in vacuo. The residues were purified by column chromatography on silica gel (petroleum ether/EtOAc = 30:1) to afford the desired 2-allylbenzaldehyde as a pale yellow oil. (*J. Org. Chem.*, **2011**, 76, 7204.)

2.3 General procedure for the synthesis of α, α-difluoroarylacetic acids 2a - 2q [3]

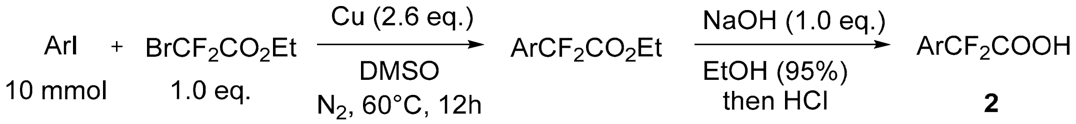

Activation of Cu powder: Copper powder (1.65 g, 26 mmol, 2.6 equiv.) was ground in a mortar with dilute hydrochloric acid (1 N, 10 mL) for 10 minutes. After filtration, the copper powder was washed with water (10 mL), methanol (10 mL) and acetone (10 mL), respectively, to give the pre-activated copper powder.

A 50 mL round bottom flask equipped with a magnetic stir bar was charged with appropriate aryl iodide (10 mmol, 1.0 equiv.), ethyl bromodifluoroacetate (1.28 mL, 10 mmol, 1.0 equiv.), pre-activated copper powder (1.65 g, 26 mmol, 2.6 equiv.), and DMSO (15 mL). The flask was evacuated and backfilled with N_2 (3 times) with the help of a three-neck joint. The reaction mixture was stirred at 60 ºC under N_2 atmosphere for 12 hours. After cooling down to room temperature, ethyl acetate (30 mL) and water (30 mL) was added. After filtration, the organic phase was separated and the aqueous phase was extracted with ethyl acetate (2 × 15 mL). The combined organic layer was washed with saturated brine (15 mL), dried over anhydrous Na_2SO_4 and concentrated in vacuo. The residue was purified by flash column chromatography on silica gel to give the corresponding ethyl α, α-difluoroaryl acetate.

The ethyl α, α-difluoroaryl acetate were reacted with NaOH (1.0 equiv.) in ethanol (95%) for 12 h at room temperature, and the sodium α, α-difluoroaryl acetate was obtained by removing the ethanol. Ether was then added to remove the residual ethyl α, α-difluoroaryl acetate. After filtration, the potassium α, α-difluoroaryl acetate was dissolved in water (20 mL) and concentrated hydrochloric acid was addeduntil pH = 1. The aqueous phase was extracted with ethyl acetate (2 × 20 mL), washed with brine (10 mL), dried over anhydrous sodium sulfate and concentrated in vacuo to give the corresponding α, α-difluoroarylacetic acids. (*Org. Lett.* **2013**, *15*, 2648.)

3. Optimization of the reaction conditions

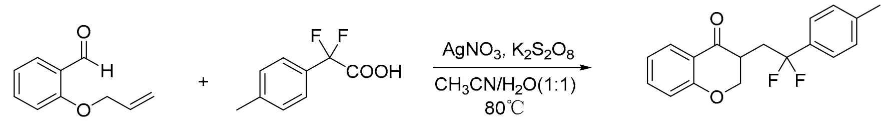

Table S1 Screening the reaction time[a]

Entry	Time (h)	Yield (%)[b]
1	1.5	43
2	2	47
3	3	42
4	4	56
5	5	46

Reaction conditions: a)**1a** (0.2 mmol), **2a** (0.3 mmol) and $K_2S_2O_8$ (0.4mmol) in CH_3CN/H_2O (2.0 mL) under N_2 atmosphere. b) Yields determined by ^{19}F NMR analysis with $PhCF_3$ as the internal standard.

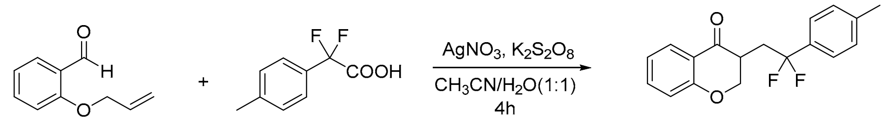

Table S2 Screening the reaction temp.[a]

Entry	Temp. (°C)	Yield (%)[b]
1	rt	NR
2	40	6
3	60	51
4	100	49
5	120	32

Reaction conditions: a)**1a** (0.2 mmol), **2a** (0.3 mmol) and $K_2S_2O_8$ (0.4mmol) in CH_3CN/H_2O (2.0 mL) under N_2 atmosphere. b) Yields determined by ^{19}F NMR analysis with $PhCF_3$ as the internal standard.

4. General procedure for the synthesis of compounds 3

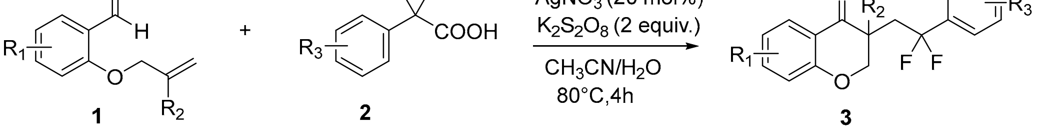

2-Allyloxyarylaldehyde **1** (0.20 mmol), $K_2S_2O_8$ (2.0 equiv, 0.40 mmol), $AgNO_3$ (20 mol%, 0.04 mmol) and α, α-difluoroarylacetic acid **2** (1.5 equiv, 0.30 mmol) were placed in a 10 mL round-bottom flask. Solvent CH_3CN/H_2O (v/v=1:3, 2.0ml) were then added under nitrogen atmosphere. The solution was stirred at 80 °C for 4 h. The resulting mixture was cooled down to room temperature and extracted with EA (5 mL × 4). The combined organic phase was dried over anhydrous Na_2SO_4. After the removal of solvent under reduced pressure, the crude product was purified by silica gel column chromatography (petroleum ether/ethyl acetate) to afford pure compound **3**.

5. Data for compounds 3

3-(2,2-difluoro-2-(*p*-tolyl)ethyl)chroman-4-one (3aa)[3]

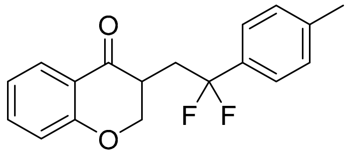

41.1 mg, 68% yield, white solid; mp 65.9 - 66.2 ºC; ^{1}H NMR (500 MHz, $CDCl_3$) δ 7.88 (dd, *J* =7.9, 1.8, 1H), 7.47 (ddd, *J* = 8.4, 7.2, 1.8 Hz, 1H), 7.42 (d, *J* = 8.1 Hz, 2H), 7.24 (d, *J* = 7.9 Hz, 2H), 7.01 (ddd, *J* = 7.9, 7.2, 1.1 Hz, 1H), 6.96 (d, *J* = 8.4 Hz, 1H), 4.74 (dd, *J* = 11.5, 5.2 Hz, 1H), 4.25 (t, *J* = 11.7 Hz, 1H), 3.19 - 2.98 (m, 2H), 2.38 (s, 3H), 2.15 - 1.95 (m, 1H); ^{19}F NMR (470 MHz, $CDCl_3$) δ -89.05 (ddd, *J* = 245.7, 23.9, 8.3 Hz), -96.85 (ddd, *J* = 245.7, 21.8 , 16.8 Hz); IR (KBr): 3039, 2926, 1684, 1609, 1468, 1383, 1311, 1211, 1168, 1034, 814

3-(2,2-difluoro-2-(4-methoxyphenyl)ethyl)chroman-4-one (3ab)[3]

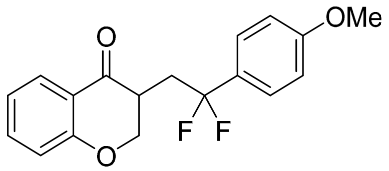

44.6 mg, 70% yield, white solid; mp 92.6 - 92.9 ºC; ^{1}H NMR (500 MHz, $CDCl_3$) δ 7.88 (dd, *J* = 7.9, 1.6 Hz, 1H), 7.50 - 7.44 (m, 3H), 7.01 (ddd, *J* = 8.0, 7.2, 1.0 Hz, 1H), 6.98 - 6.92 (m, 3H), 4.73 (dd, *J* = 11.4, 5.1 Hz, 1H), 4.25 (t, *J* = 11.7 Hz, 1H), 3.83 (s, 3H), 3.18 - 3.00 (m, 2H), 2.13 - 2.00 (m, 1H); ^{19}F NMR (470 MHz, $CDCl_3$) δ -87.69 (ddd, *J* = 245.6, 23.2, 8.0 Hz), -95.82 (ddd, *J* = 245.5, 21.5, 16.9 Hz); IR (KBr): 3054, 2916, 1696, 1609, 1515, 1471, 1384, 1170, 824, 754

3-(2-(4-(tert-butyl)phenyl)-2,2-difluoroethyl)chroman-4-one (3ac) [3]

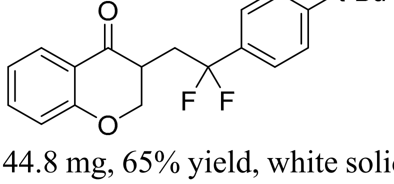

44.8 mg, 65% yield, white solid; mp 84.5 - 85.1 ºC; ^{1}H NMR (500 MHz, $CDCl_3$) δ 7.88 (dd, *J* = 7.9, 1.7 Hz, 1H), 7.46 (s, 5H), 7.01 (ddd, *J* = 8.0, 7.2, 1.0 Hz, 1H), 6.97 (dd, *J* = 8.4, 0.6 Hz, 1H), 4.77 (dd, *J* = 11.6, 5.2 Hz, 1H), 4.27 (t, *J* = 11.7 Hz, 1H), 3.20 - 3.03 (m, 2H), 2.28 - 1.92 (m, 1H), 1.33 (s, 9H); ^{19}F NMR (470 MHz, $CDCl_3$) δ -88.70 (ddd, *J* = 246.2, 23.8, 8.0 Hz), -96.97 (ddd, *J* = 246.2, 22.1, 16.9 Hz); IR (KBr): 3066, 2962, 1689, 1611, 1468, 1408, 1214, 1168, 1102, 834, 753

3-(2-([1,1'-biphenyl]-4-yl)-2,2-difluoroethyl)chroman-4-one (3ad) [3]

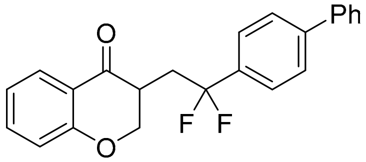

44.5 mg, 61% yield, white solid; mp 156.3-156.6 ºC; ^{1}H NMR (500 MHz, $CDCl_3$) δ 7.91 (d, J = 7.5 Hz, 1H), 7.67 (d, J = 8.0 Hz, 2H), 7.65 - 7.57 (m, 4H), 7.48 (q, J = 7.7 Hz, 3H), 7.40 (t, J = 7.2 Hz, 1H), 7.03 (t, J = 7.4 Hz, 1H), 6.99 (d, J = 8.3 Hz, 1H), 4.80 (dd, J = 11.3, 5.0 Hz, 1H), 4.29 (t, J = 11.6 Hz, 1H), 3.35 - 3.04 (m, 2H), 2.20 - 2.09 (m, 1H); ^{19}F NMR (470 MHz, $CDCl_3$) δ -89.39 (ddd, J = 246.3, 24.5, 8.1 Hz), -97.10 (ddd, J = 246.3, 22.4, 16.4 Hz); IR (KBr): 3060, 2922, 1693, 1608, 1570, 1472, 1168, 1046, 832, 756, 685

3-(2,2-difluoro-2-(m-tolyl)ethyl)chroman-4-one (3ae)

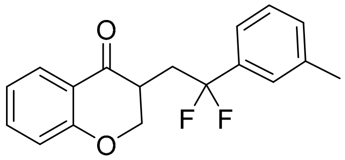

35.1 mg, 58% yield, light yellow solid; mp 67.3 - 67.6 ºC; ^{1}H NMR (500 MHz, $CDCl_3$) δ 7.89 (dd, J = 7.9, 1.7 Hz, 1H), 7.47 (ddd, J = 8.5, 7.2, 1.8 Hz, 1H), 7.36 - 7.30 (m, 3H), 7.25 (s, 1H), 7.01 (t, J = 8.0 Hz, 1H), 6.97 (d, J = 9.0 Hz, 1H), 4.76 (dd, J = 11.4, 5.1 Hz, 1H), 4.26 (t, J = 11.7 Hz, 1H), 3.24 - 2.96 (m, 2H), 2.40 (s, 3H), 2.19 - 1.93 (m, 1H); ^{19}F NMR (470 MHz, $CDCl_3$) δ -89.87 (ddd, J = 245.2, 24.6, 8.1 Hz), -97.24 (ddd, J = 245.1, 22.3, 16.2 Hz); ^{13}C NMR (125 MHz, $CDCl_3$) δ 192.1, 161.6, 138.5, 136.5 (t, $^2J_{C-F}$ = 26.0 Hz), 136.0, 130.9, 128.6, 127.5, 125.5 (t, $^3J_{C-F}$ = 6.1 Hz), 122.8 (t, $^1J_{C-F}$ = 243.4 Hz), 122.0 (t, $^3J_{C-F}$ = 6.2 Hz), 121.5, 120.4, 117.8, 70.5, 41.3, 34.3 (t, $^2J_{C-F}$ = 28.1 Hz), 21.5; IR (KBr): 3065, 2924, 1687, 1606, 1460, 1369, 1149, 1077, 764, 702, 665; HRMS (ESI) calcd. for $C_{18}H_{16}F_2O_2$ $[M+H]^+$ 303.1197, found: 303.1193

3-(2,2-difluoro-2-(*o*-tolyl)ethyl)chroman-4-one (3af) [3]

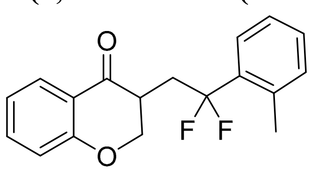

38.1 mg, 63% yield, light yellow oil; ^{1}H NMR (500 MHz, $CDCl_3$) δ 7.89 (dd, J = 7.7, 1.9 Hz, 1H), 7.51 (d, J = 7.9 Hz, 1H), 7.48 (ddd, J = 8.9, 7.2, 1.8 Hz, 1H), 7.33 (t, J = 7.4 Hz, 1H), 7.25 (t, J = 8.0 Hz, 2H), 7.02 (ddd, J = 8.1, 7.2, 1.0 Hz, 1H), 6.98 (d, J = 8.7 Hz, 1H), 4.78 (dd, J = 11.6, 5.2 Hz, 1H), 4.27 (t, J = 11.7 Hz, 1H), 3.26 - 3.06 (m, 2H), 2.52 (s, 3H), 2.20 -1.98 (m, 1H); ^{19}F NMR δ -89.84 (ddd, J = 248.7, 26.3, 7.5 Hz), -94.21 (ddd, J = 248.5, 23.5, 14.8 Hz); IR (KBr): 3070, 2926, 1692, 1608, 1374, 1159, 1078, 763

3-(2-(3,4-dimethylphenyl)-2,2-difluoroethyl)chroman-4-one (3ag)

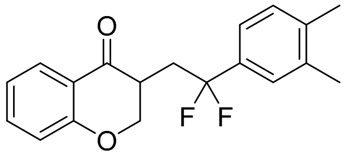

58.2 mg, 92% yield, light yellow solid; mp 84.5 - 84.9 °C; ^{1}H NMR (500 MHz, $CDCl_3$) δ 7.89 (dd, *J* = 7.9, 1.7 Hz, 1H), 7.47 (ddd, *J* = 8.8, 7.2, 1.8 Hz, 1H), 7.31 (s, 1H), 7.27 (d, *J* = 8.0 Hz, 1H), 7.19 (d, *J* = 7.8 Hz, 1H), 7.01 (ddd, *J* = 8.0, 7.3, 1.0 Hz, 1H), 6.97 (d, *J* = 8.4 Hz, 1H), 4.75 (dd, *J* = 11.5, 5.1 Hz, 1H), 4.25 (t, *J* = 11.7 Hz, 1H), 3.27 - 2.89 (m, 2H), 2.31 (s, 3H), 2.29 (s, 3H), 2.14 - 1.99 (m, 1H); ^{19}F NMR (470 MHz, $CDCl_3$) δ -88.88 (ddd, *J* = 245.1, 23.9, 8.1 Hz), -96.66 (dt, *J* = 245.1, 19.0 Hz); ^{13}C NMR (125 MHz, $CDCl_3$) δ 192.2, 161.7, 138.8, 137.1, 136.0, 134.0 (t, $^2J_{C\text{-}F}$ = 26.2 Hz), 129.8, 127.5, 126.0 (t, $^3J_{C\text{-}F}$ = 6.0 Hz), 122.9 (t, $^1J_{C\text{-}F}$ = 242.8 Hz), 122.3 (t, $^3J_{C\text{-}F}$ = 6.2 Hz), 121.5, 120.4, 117.8, 70.6, 41.3, 35.3 (t, $^2J_{C\text{-}F}$ = 28.6 Hz), 19.9, 19.6; IR (KBr): 3051, 2925, 1688, 1607, 1467, 1393, 1138, 1028, 822, 767, 681; HRMS (ESI) calcd. for $C_{19}H_{18}F_2O_2$ $[M+H]^+$ 317.1353, found: 317.1359

3-(2,2-difluoro-2-mesitylethyl)chroman-4-one (3ah)

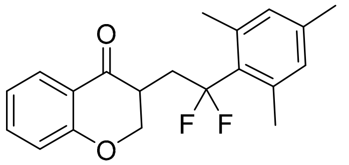

36.3 mg, 55% yield, light yellow oil; ^{1}H NMR (500 MHz, $CDCl_3$) δ 7.91 (dd, *J* = 7.9, 1.7 Hz, 1H), 7.49 (ddd, *J* = 8.5, 7.2, 1.8 Hz, 1H), 7.03 (ddd, *J* = 8.1, 7.3, 1.0 Hz, 1H), 7.00 (d, *J* = 8.4 Hz, 1H), 6.87 (s, 2H), 4.86 (dd, *J* = 11.3, 5.2 Hz, 1H), 4.34 (t, *J* = 11.6 Hz, 1H), 3.34 (dddd, *J* = 11.8, 8.5, 5.2, 2.7 Hz, 1H), 3.04 (dddd, *J* = 33.1, 15.9, 7.2, 2.7 Hz, 1H), 2.43 (t, *J* = 4.4 Hz, 6H), 2.27 (s, 3H), 2.16 - 2.01 (m, 1H); ^{19}F NMR (470 MHz, $CDCl_3$) δ -85.20 – -86.00 (m), -88.96 (dd, *J* = 250.6, 28.9 Hz); ^{13}C NMR (125 MHz, $CDCl_3$) δ 192.5, 161.7, 139.0, 136.0, 136.0 (t, $^3J_{C\text{-}F}$ = 3.6 Hz), 131.2, 130.9 (t, $^2J_{C\text{-}F}$ = 23.6 Hz), 127.5, 125.4 (t, $^1J_{C\text{-}F}$ = 244.3 Hz), 121.5, 120.5, 117.9, 70.8 (d, $^3J_{C\text{-}F}$ = 4.7 Hz), 41.0, 33.7 (t, $^2J_{C\text{-}F}$ = 26.7 Hz), 22.2 (t, $^3J_{C\text{-}F}$ = 6.5 Hz), 20.7; IR (KBr): 3027, 2926, 1693, 1604, 1467, 1375, 1160, 1037, 860, 761; HRMS (ESI) calcd. for $C_{20}H_{20}F_2O_2$ $[M+H]^+$ 331.1510, found: 331.1511

3-(2,2-difluoro-2-phenylethyl)chroman-4-one (3ai) [3]

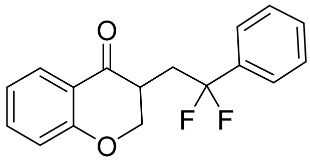

28.8 mg, 50% yield, white solid. mp 68.0 - 68.5 °C; ^{1}H NMR (500 MHz, $CDCl_3$) δ 7.88 (dd, *J* = 7.9, 1.7 Hz, 1H), 7.58 - 7.52 (m, 2H), 7.51 - 7.46 (m, 1H), 7.46 - 7.42 (m, 3H), 7.05 - 6.99 (m, 2H), 6.97 (d, *J* = 8.4 Hz, 1H), 4.75 (dd, *J* = 11.4, 5.0 Hz, 1H), 4.26 (t, *J* = 11.6 Hz, 1H), 3.19 - 3.03 (m, 1H), 2.14 - 2.03 (m, 1H); ^{19}F NMR (470 MHz, $CDCl_3$) δ -90.06 (ddd, *J* = 246.0, 24.6, 8.2 Hz), -97.48 (ddd, *J* = 245.7, 22.4, 15.8 Hz); IR (KBr): 3065, 2917, 1695, 1605, 1470, 1129, 1043, 756, 692

3-(2-(benzo[d][1,3]dioxol-5-yl)-2,2-difluoroethyl)chroman-4-one (3aj)

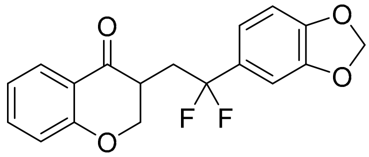

27.6 mg, 41% yield, light yellow solid; mp 46.3 - 46.5 ºC; ^{1}H NMR (500 MHz, $CDCl_3$) δ 7.87 (dd, *J* = 7.9, 1.7 Hz, 1H), 7.47 (ddd, *J* = 8.6, 7.2, 1.7 Hz, 1H), 7.06 - 6.92 (m, 4H), 6.83 (d, *J* = 8.1 Hz, 1H), 5.99 (s, 2H), 4.72 (dd, *J* = 11.4, 5.0 Hz, 1H), 4.24 (t, *J* = 11.7 Hz, 1H), 3.17 - 2.93 (m, 2H), 2.11 - 1.98 (m, 1H); ^{19}F NMR (470 MHz, $CDCl_3$) δ -87.57 (ddd, *J* = 244.5, 23.5, 8.1 Hz), -95.50 (ddd, *J* = 244.3, 21.7, 16.7 Hz); ^{13}C NMR (125 MHz, $CDCl_3$) δ 192.1, 161.6, 149.0, 148.0, 136.1, 130.4 (t, $^{2}J_{C\text{-}F}$ = 26.6 Hz), 127.5, 122.6 (t, $^{1}J_{C\text{-}F}$ = 243.1 Hz), 121.5, 120.4, 119.0 (t, $^{3}J_{C\text{-}F}$ = 6.8 Hz), 117.8, 108.2, 105.7 (t, $^{3}J_{C\text{-}F}$ = 6.2 Hz), 101.6, 70.5, 41.3, 34.3 (t, $^{2}J_{C\text{-}F}$ = 28.5 Hz); IR (KBr): 3045, 2920, 1691, 1604, 1498, 1447, 1101, 1038, 815, 758, 664; HRMS (ESI) calcd. for $C_{18}H_{14}F_2O_4$ $[M+H]^+$ 333.0938, found: 333.0934

3-(2,2-difluoro-2-(4-fluorophenyl)ethyl)chroman-4-one (3ak)

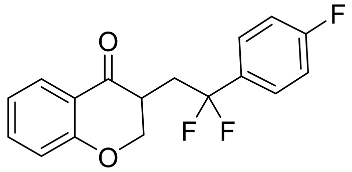

24.5 mg, 40% yield, white solid; mp 93.6 - 93.9 ºC; ^{1}H NMR (500 MHz, $CDCl_3$) δ 7.88 (dd, *J* = 7.9, 1.7 Hz, 1H), 7.53 (dd, *J* = 8.8, 5.2 Hz, 2H), 7.48 (ddd, *J* = 8.6, 7.2, 1.8 Hz, 1H), 7.12 (t, *J* = 8.6 Hz, 2H), 7.06 - 6.99 (m, 1H), 6.97 (d, *J* = 8.4 Hz, 1H), 4.75 (dd, *J* = 11.3, 5.2 Hz, 1H), 4.26 (t, *J* = 11.6 Hz, 1H), 3.22 - 2.93 (m, 1H), 2.24 - 1.89 (m, 1H); ^{19}F NMR (470 MHz, $CDCl_3$) δ -88.92 (ddd, *J* = 247.0, 24.8, 7.1 Hz), -96.62 (ddd, *J* = 246.6, 22.5, 15.7 Hz), -110.5; ^{13}C NMR (125MHz, $CDCl_3$) δ 192.0, 163.6 (d, $^{1}J_{C\text{-}F}$ = 250.0 Hz), 161.6, 136.1, 132.6 (td, $^{2}J_{C\text{-}F}$ = 26.8 Hz, $^{4}J_{C\text{-}F}$ = 3.2 Hz), 127.5, 127.2 (dt, $^{3}J_{C\text{-}F}$ = 6. 3 Hz, $^{3}J_{C\text{-}F}$ = 6. 2 Hz), 122.9 (t, $^{1}J_{C\text{-}F}$ = 243.3 Hz), 121.6, 120.3, 117.8, 115.8 (d, $^{2}J_{C\text{-}F}$ = 22.0 Hz), 70.5, 41.2, 34.3 (t, $^{2}J_{C\text{-}F}$ = 28.0 Hz); IR (KBr): 3069, 2922, 1696, 1609, 1515, 1472, 1237, 1164, 1099, 832, 754; HRMS (ESI) calcd. for $C_{17}H_{13}F_3O_2$ $[M+H]^+$ 307.0946, found: 307.0945

3-(2-(4-chlorophenyl)-2,2-difluoroethyl)chroman-4-one (3al) [3]

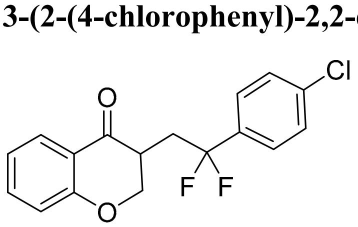

22.6 mg, 35% yield, light yellow solid; mp 66.5 - 66.9 ºC; ^{1}H NMR (500 MHz, $CDCl_3$) δ 7.88 (dd, *J* = 7.9, 1.6 Hz, 1H), 7.52 - 7.45 (m, 3H), 7.42 (d, *J* = 8.7 Hz, 2H), 7.02 (ddd, *J* = 8.0, 7.2, 1.0 Hz, 1H), 6.97 (dd, *J* = 8.4, 0.7 Hz, 1H), 4.75 (dd, *J* = 11.3, 5.1 Hz, 1H),

4.26 (t, J = 11.5 Hz, 1H), 3.22 - 2.96 (m, 2H), 2.18 - 1.95 (m, 1H); ^{19}F NMR (470 MHz, $CDCl_3$) δ -89.90 (ddd, J = 246.9, 24.9, 7.8 Hz), -97.47 (ddd, J = 246.8, 22.8, 15.4 Hz); IR (KBr): 3066, 2962, 1689, 1611, 1570, 1470, 1094, 1001, 827, 756, 654

3-(2-(4-bromophenyl)-2,2-difluoroethyl)chroman-4-one (3am) [3]

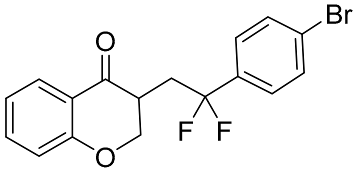

23.5 mg, 32% yield, light yellow solid; mp 85.3 - 85.8 °C; ^{1}H NMR (500 MHz, $CDCl_3$) δ 7.88 (dd, J = 7.9, 1.5 Hz, 1H), 7.58 (d, J = 8.6 Hz, 2H), 7.48 (ddd, J = 8.4, 7.2, 1.8 Hz, 1H), 7.41 (d, J = 8.6 Hz, 2H), 7.02 (ddd, J = 8.0, 7.2, 1.0 Hz, 1H), 6.99 - 6.94 (m, 1H), 4.75 (dd, J = 11.4, 5.1 Hz, 1H), 4.26 (t, J = 11.6 Hz, 1H), 3.20 - 2.93 (m, 2H), 2.16 - 1.94 (m, 1H); ^{19}F NMR (470 MHz, $CDCl_3$) δ -90.11 (ddd, J = 246.9, 25.0, 7.7 Hz), -97.66 (ddd, J = 246.8, 22.9, 15.4 Hz); IR (KBr): 3072, 2929, 1693, 1605, 1472, 1064, 1001, 823, 757, 654, 564; HRMS (ESI) calcd. for $C_{17}H_{13}BrF_2O_2$ $[M+H]^+$ 367.0145, found: 367.0146

3-(2-(3-bromophenyl)-2,2-difluoroethyl)chroman-4-one (3an)

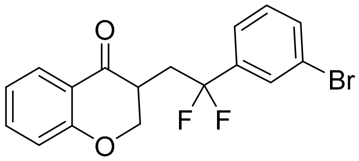

19.1 mg, 26% yield, white solid; mp 60.1 - 60.4 °C; ^{1}H NMR (500 MHz, $CDCl_3$) δ 7.88 (dd, J = 8.0, 1.6 Hz, 1H), 7.69 (s, 1H), 7.59 (d, J = 8.0 Hz, 1H), 7.51 - 7.45 (m, 2H), 7.33 (t, J = 7.9 Hz, 1H), 7.03 (ddd, J = 8.0, 7.2, 1.0 Hz, 1H), 6.98 (d, J = 8.4 Hz, 1H), 4.77 (dd, J = 11.3, 5.2 Hz, 1H), 4.27 (t, J = 11.7 Hz, 1H), 3.21 - 2.94 (m, 2H), 2.17 - 1.97 (m, 1H); ^{19}F NMR (470 MHz, $CDCl_3$) δ -90.78 (ddd, J = 246.6, 26.2, 7.6 Hz), -97.89 (ddd, J = 246.6, 23.7, 14.5 Hz); ^{13}C NMR (125 MHz, $CDCl_3$) δ 191.9, 161.7, 138.7 (t, $^{2}J_{C\text{-}F}$ = 26.9 Hz), 136.2, 133.3, 130.3, 128.2 (t, $^{3}J_{C\text{-}F}$ = 6.4 Hz), 127.6, 123.6 (t, $^{3}J_{C\text{-}F}$ = 6.0 Hz), 122.8, 121.8 (t, $^{1}J_{C\text{-}F}$ = 243.9 Hz), 121.6, 120.3, 117.8, 70.5, 41.1, 34.3 (t, $^{2}J_{C\text{-}F}$ = 27.5 Hz); IR (KBr): 3035, 2923, 1693, 1603, 1470, 1120, 1064, 1001, 794, 753, 689, 591; HRMS (ESI) calcd. for $C_{17}H_{13}BrF_2O_2$ $[M+H]^+$ 367.0145, found: 367.0141

3-(2,2-difluoro-2-(4-iodophenyl)ethyl)chroman-4-one (3ao)

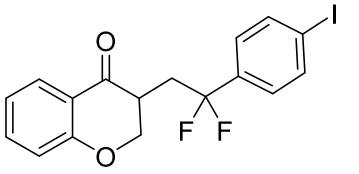

49.7 mg, 60% yield, light yellow solid; mp 116.0 -116.5 °C; ^{1}H NMR (500 MHz,$CDCl_3$) δ 7.87 (dd, J = 8.2, 1.7 Hz, 1H), 7.78 (d, J = 8.5 Hz, 2H), 7.47 (ddd, J = 8.4, 7.2, 1.8 Hz, 1H), 7.27 (d, J = 8.3 Hz, 2H), 7.01 (ddd, J = 8.1, 7.2, 1.0 Hz, 1H), 6.96 (d, J = 8.4 Hz, 1H), 4.74 (dd, J = 11.4, 5.1 Hz, 1H), 4.25 (t, J = 11.6 Hz, 1H), 3.16 - 2.96 (m, 2H), 2.10 - 1.99 (m, 1H); ^{19}F NMR (470 MHz, CDCl3) δ -90.34 (ddd, J = 246.9, 24.9, 7.7 Hz), -97.88 (ddd, J = 246.8, 22.8, 15.4 Hz); ^{13}C NMR (125 MHz, $CDCl_3$) δ 191.9, 161.6, 137.9, 136.2, 136.2 (t, $^{2}J_{C\text{-}F}$ = 26.6 Hz), 127.6, 126.7 (t, $^{3}J_{C\text{-}F}$ = 5.8 Hz), 122.4 (t, $^{1}J_{C\text{-}F}$ = 243.6 Hz), 121.6, 120.3, 117.8, 96.6, 70.4, 41.1, 34.1 (t, $^{2}J_{C\text{-}F}$ = 27.8 Hz); IR (KBr): 3009, 2905, 1689, 1595, 1466, 1112, 1021, 826, 763, 555; HRMS (ESI) calcd. for $C_{17}H_{13}F_2IO_2$ $[M+H]^+$ 415.0007, found: 415.0011

3-(2,2-difluoro-2-((4-methoxyphenyl)thio)ethyl)chroman-4-one (3aq)

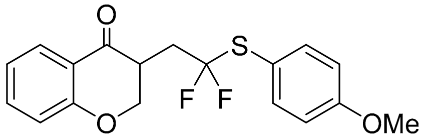

21.0 mg, 30% yield, white solid; mp 67.5 - 68.0 °C; ^{1}H NMR (500MHz, $CDCl_3$) δ 7.89 (dd, J = 7.9, 1.7 Hz, 1H), 7.53 (d, J = 8.8 Hz, 2H), 7.49 (ddd, J = 8.8, 7.2, 1.8 Hz, 1H), 7.03 (ddd, J = 8.0, 7.3, 1.0 Hz, 1H), 6.98 (d, J = 9.1 Hz, 1H), 6.91 (d, J = 8.9 Hz, 2H), 4.75 (dd, J = 11.4, 5.3 Hz, 1H), 4.22 (t, J = 11.8 Hz, 1H), 3.83 (s, 3H), 3.33 - 3.19 (m, 1H), 3.11 - 3.00 (m, 1H), 2.15 - 1.95 (m, 1H); ^{19}F NMR (470 MHz, $CDCl_3$) δ -69.65 (ddd, J = 207.3, 19.6, 8.6 Hz), -74.59 (dt, J = 207.2, 17.4 Hz); ^{13}C NMR (125 MHz, $CDCl_3$) δ 191.6, 161.6, 161.3, 138.2, 136.1, 129.4 (t, $^{1}J_{C\text{-}F}$ = 278.2 Hz), 127.6, 121.6, 120.3, 117.8, 116.8, 114.8, 70.3, 55.4, 41.7, 33.8 (t, $^{2}J_{C\text{-}F}$ = 24.6 Hz); IR (KBr): 3052, 2929, 1688, 1596, 1469, 1394, 1153, 1026, 960, 823, 761; HRMS (ESI) calcd. for $C_{18}H_{16}F_2O_3S$ $[M+H]^+$ 351.0866, found: 351.0865

2-((2,2-difluoro-6-methoxythiochroman-4-yl)methoxy)benzaldehyde (3aq')

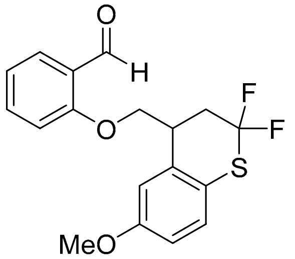

20.3 mg, 29% yield, white solid; mp 64.0 - 64.5 °C; ^{1}H NMR (500MHz, $CDCl_3$) δ 10.48 (s, 1H), 7.84 (dd, J = 7.7, 1.8 Hz, 1H), 7.54 (ddd, J = 9.1, 7.4, 1.8 Hz, 1H), 7.12 (d, J = 8.6 Hz, 1H), 7.06 (t, J = 7.5 Hz, 1H), 6.99 (d, J = 8.4 Hz, 1H), 6.91 (d, J = 2.7 Hz, 1H), 6.83 (dd, J = 8.6, 2.7 Hz, 1H), 4.46 - 4.31 (m, 2H), 3.78 (s, 3H), 3.61 - 3.56 (m, 1H), 2.81 - 2.72 (m, 1H), 2.66 - 2.58 (m, 1H); ^{19}F NMR (470 MHz, $CDCl_3$) δ -60.89 (ddd, J = 219.7, 22.1, 13.6 Hz), -64.51 (dt, J = 219.8, 12.3 Hz); ^{13}C NMR (125 MHz, $CDCl_3$) δ 189.2, 160.6, 158.4, 136.0, 134.5, 130.5 (t, $^{1}J_{C\text{-}F}$ = 268.7 Hz) 128.8, 128.3 (t, $^{3}J_{C\text{-}F}$ = 2.9 Hz), 125.0, 121.3, 120.8, 115.1, 113.7, 112.5, 68.2 (d, $^{3}J_{C\text{-}F}$ = 3.4 Hz), 55.5, 39.0 (d, ^{3}J = 5.6 Hz), 37.6 (t, $^{2}J_{C\text{-}F}$ = 23.8 Hz); IR (KBr): 3067, 2926, , 1739, 1678, 1599, 1568,

1496, 1074, 1022, 990, 890, 806, 760; HRMS (ESI) calcd. for $C_{18}H_{16}F_2O_3S$ $[M+H]^+$ 351.0866, found: 351.0863

3-(2,2-difluorobutyl)chroman-4-one (3ar)

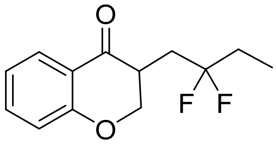

6.3 mg, 13% yield, light yellow oil; ^{1}H NMR (500 MHz, $CDCl_3$) δ 7.90 (dd, J = 7.9, 1.8 Hz, 1H), 7.53 - 7.44 (m, 1H), 7.02 (ddd, J = 8.1, 7.2, 1.1 Hz, 1H), 6.98 (d, J = 8.4 Hz, 1H), 4.76 (dd, J = 11.4, 5.2 Hz, 1H), 4.24 (t, J = 11.7 Hz, 1H), 3.17 (dddd, J = 12.1, 8.9, 5.3, 3.0 Hz, 1H), 2.74 (dddd, J = 29.4, 15.4, 10.9, 3.0 Hz, 1H), 2.00 - 1.87 (m, 2H), 1.80 (dddd, J = 24.8, 15.8, 9.3, 6.9 Hz, 1H), 1.06 (t, J = 7.5 Hz, 3H); ^{19}F NMR (470 MHz, $CDCl_3$) δ -96.70 - -98.13 (m), -100.51- -101.83 (m); ^{13}C NMR (125 MHz, $CDCl_3$) δ 192.6, 161.7, 136.0, 127.5, 125.0 (t, $^1J_{C\text{-}F}$ = 242.0 Hz), 121.5, 120.4, 117.8, 70.7, 40.9, 31.3 (t, $^2J_{C\text{-}F}$ = 25.4 Hz), 30.5 (t, $^2J_{C\text{-}F}$ = 26.0 Hz), 6.6 (t, $^3J_{C\text{-}F}$ = 5.7 Hz); IR (KBr): 3041, 2928, 1694, 1609, 1468, 1370, 1137, 1040, 764, 673; HRMS (ESI) calcd. for $C_{13}H_{14}F_2O_2$ $[M+H]^+$ 241.1040, found: 241.1037

3-(2,2-difluoro-2-(4-methoxyphenyl)ethyl)-7-methoxychroman-4-one (3bb)

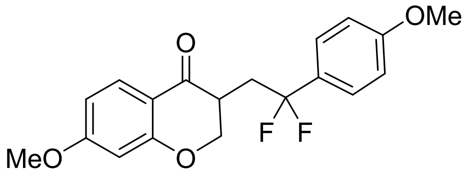

36.2 mg, 52% yield, white solid; mp 105.9 - 106.1 ºC; ^{1}H NMR (500 MHz, $CDCl_3$) δ 7.81 (d, J = 8.8 Hz, 1H), 7.46 (d, J = 8.8 Hz, 2H), 6.93 (d, J = 8.7 Hz, 2H), 6.57 (dd, J = 8.6, 2.6 Hz, 1H), 6.39 (d, J = 2.3 Hz, 1H), 4.70 (dd, J = 11.2, 5.2 Hz, 1H), 4.22 (t, J = 11.5 Hz, 1H), 3.82 (s, 6H), 3.32 - 2.85 (m, 2H), 2.31 - 1.82 (m, 1H); ^{19}F NMR (470 MHz, $CDCl_3$) δ -87.41 (ddd, J = 245.4, 23.5, 8.1 Hz), -95.84 (dt, J = 248.5, 20.6 Hz); ^{13}C NMR (125 MHz, $CDCl_3$) δ 190.8, 166.0, 163.6, 160.8, 129.2, 128.8 (t, $^2J_{C\text{-}F}$ = 26.8 Hz), 126.4 (t, $^3J_{C\text{-}F}$ = 6.1 Hz), 123.0 (t, $^1J_{C\text{-}F}$ = 242.2 Hz), 114.2, 113.9, 110.1, 100.6, 70.9, 55.6, 55.4, 40.9, 34.3 (t, $^2J_{C\text{-}F}$ = 28.5 Hz); IR (KBr): 3021, 2957, 1690, 1614, 1511, 1460, 1374, 1614, 1167, 1026, 879, 830; HRMS (ESI) calcd. For $C_{19}H_{18}F_2O_4$ $[M+H]^+$ 349.1251, found: 349.1248

3-(2,2-difluoro-2-(4-methoxyphenyl)ethyl)-6-methylchroman-4-one (3cb)

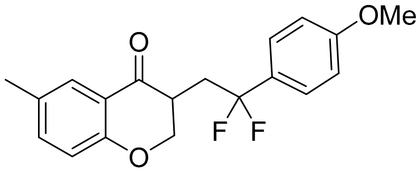

23.3 mg, 35% yield, white solid; mp 69.6 -70.1 ºC; ^{1}H NMR (500 MHz, $CDCl_3$) δ 7.66 (d, J = 1.9 Hz, 1H), 7.46 (d, J = 8.8 Hz, 2H), 7.28 (dd, J = 8.9, 2.3 Hz, 1H), 6.94 (d, J

= 8.8 Hz, 2H), 6.86 (d, J = 8.5 Hz, 1H), 4.69 (dd, J = 11.2, 5.1 Hz, 1H), 4.21 (t, J = 11.5 Hz, 1H), 3.83 (s, 3H), 3.21 - 2.94 (m, 2H), 2.30 (s, 3H), 2.12 - 2.00 (m, 1H); ^{19}F NMR (470 MHz, $CDCl_3$) δ -87.69 (ddd, J = 245.5, 23.3, 8.2 Hz), -95.74 (ddd, J = 245.5, 21.5, 16.7 Hz); ^{13}C NMR (125 MHz, $CDCl_3$) δ 192.4, 160.8, 159.7, 137.1, 130.9, 128.8 (t, $^2J_{C\text{-}F}$ = 26.8 Hz), 127.0, 126.4 (t, $^3J_{C\text{-}F}$ = 6.1 Hz), 122.9 (t, $^1J_{C\text{-}F}$ = 242.2 Hz), 120.0, 117.6, 113.9, 70.5, 55.4, 41.4, 34.3 (t, $^2J_{C\text{-}F}$ = 28.8 Hz), 20.4; IR (KBr): 3027, 2916, 1697, 1617, 1501, 1424, 1302, 1169, 1028, 823, 735; HRMS (ESI) calcd. for $C_{19}H_{18}F_2O_3$ $[M+H]^+$ 333.1302, found: 333.1306

3-(2,2-difluoro-2-(4-methoxyphenyl)ethyl)-7-methylchroman-4-one (3db)

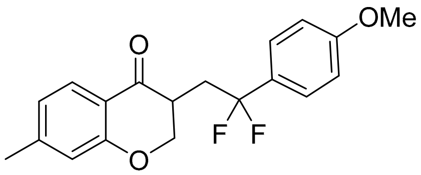

33.2 mg, 50% yield, white solid; mp 64.4 - 64.7 ºC; ^{1}H NMR (500 MHz, $CDCl_3$) δ 7.76 (d, J = 8.0 Hz, 1H), 7.46 (d, J = 8.8 Hz, 2H), 6.93 (d, J = 8.8 Hz, 2H), 6.82 (ddd, J = 8.0, 1.5, 0.5 Hz, 1H), 6.76 (s, 1H), 4.70 (dd, J = 11.4, 5.2 Hz, 1H), 4.21 (t, J = 11.6 Hz, 1H), 3.82 (s, 3H), 3.20 - 2.88 (m, 2H), 2.35 (s, 3H), 2.05 (dddd, J = 22.4, 15.9, 9.7, 8.7 Hz, 1H); ^{19}F NMR (470 MHz, $CDCl_3$) δ -87.58 (ddd, J = 245.5, 23.2, 8.1 Hz), -95.75 (ddd, J = 245.4, 21.5, 16.8 Hz); ^{13}C NMR (125 MHz, $CDCl_3$) δ 191.9, 161.7, 160.8, 147.6, 128.8 (t, $^2J_{C\text{-}F}$ = 26.9 Hz), 127.4, 126.4 (t, $^3J_{C\text{-}F}$ = 6.1 Hz), 122.9 (t, $^1J_{C\text{-}F}$ = 242.0 Hz), 122.9, 118.1, 117.8, 113.9, 70.5, 55.4, 41.2, 34.2 (t, $^2J_{C\text{-}F}$ = 28.6 Hz), 21.9; IR (KBr): 3019, 2934, 1684, 1612, 1513, 1319, 1167, 974, 827; HRMS (ESI) calcd. for $C_{19}H_{18}F_2O_3$ $[M+H]^+$ 333.1302, found: 333.1300

6,8-di-tert-butyl-3-(2,2-difluoro-2-(4-methoxyphenyl)ethyl)chroman-4-one (3eb)

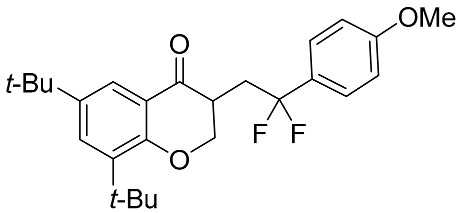

38.8 mg, 45% yield, light yellow oil; ^{1}H NMR (500 MHz, $CDCl_3$) δ 7.79 (d, J = 2.5 Hz, 1H), 7.54 (d, J = 2.6 Hz, 1H), 7.48 (d, J = 8.8 Hz, 2H), 6.95 (d, J = 8.8 Hz, 2H), 4.79 (s, 1H), 4.22 (t, J = 11.5 Hz, 1H), 3.83 (s, 3H), 3.18 - 2.97 (m, 2H), 2.18 - 2.00 (m, 1H), 1.40 (s, 9H), 1.30 (s, 9H); ^{19}F NMR (470 MHz, $CDCl_3$) δ -87.06 (ddd, J = 245.5, 23.6, 7.9 Hz), -95.98 (ddd, J = 245.5, 21.8, 16.8 Hz); ^{13}C NMR (125 MHz, $CDCl_3$) δ 193.2, 160.8, 158.8, 143.4, 138.3, 130.8, 128.9 (t, $^2J_{C\text{-}F}$ = 26.8 Hz), 126.5 (t, $^3J_{C\text{-}F}$ = 6.1 Hz), 123.0 (t, $^1J_{C\text{-}F}$ = 242.4 Hz), 121.5, 120.4, 113.9, 70.0, 55.4, 41.3, 35.1, 34.5, 34.4 (t, $^2J_{C\text{-}F}$ = 28.4 Hz), 31.3, 29.7; IR (KBr): 3006, 2920, 1682, 1613, 1471, 1364, 1171,1023, 846, 758, 678; HRMS (ESI) calcd. for $C_{26}H_{32}F_2O_3$ $[M+H]^+$ 431.2398, found: 431.2395

3-(2,2-difluoro-2-(4-methoxyphenyl)ethyl)-6-fluorochroman-4-one (3fb)

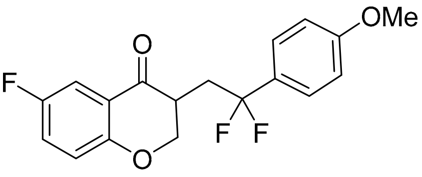

34.3 mg, 51% yield, white solid; mp 112.4 - 112.8 ºC; ^{1}H NMR (500 MHz, $CDCl_3$) δ 7.52 (dd, *J* = 8.3, 3.2 Hz, 1H), 7.46 (d, *J* = 8.8 Hz, 2H), 7.20 (ddd, *J* = 9.1, 7.7, 3.2 Hz, 1H), 6.95 (dd, *J* = 8.6, 5.0 Hz, 3H), 4.73 (dd, *J* = 11.7, 5.2 Hz, 1H), 4.23 (t, *J* = 11.8 Hz, 1H), 3.83 (s, 3H), 3.15 - 3.00 (m, 2H), 2.13 - 2.00 (m, 1H); ^{19}F NMR (470 MHz, $CDCl_3$) δ -87.90 (ddd, *J* = 245.6, 23.8, 8.2 Hz), -95.81 (ddd, *J* = 245.5, 21.9, 16.3 Hz), -121.27 (td, *J* = 8.1, 4.3 Hz); ^{13}C NMR (125 MHz, $CDCl_3$) δ 191.5, 160.9, 158.2, 157.9 (d, $^4J_{C\text{-}F}$ = 1.7 Hz), 156.3, 128.6 (t, $^2J_{C\text{-}F}$ = 26.8 Hz), 126.4 (dd, $^3J_{C\text{-}F}$ = 6.4Hz, *J* = 5.6Hz), 123.6 (d, $^2J_{C\text{-}F}$ = 24.6 Hz), 122.7 (t, $^1J_{C\text{-}F}$ = 242.1 Hz), 120.7 (d, $^3J_{C\text{-}F}$ = 6.6 Hz), 119.5 (d, $^3J_{C\text{-}F}$ = 7.3 Hz), 112.4 (d, $^2J_{C\text{-}F}$ = 23.4 Hz), 70.7, 55.6, 41.2, 34.2 (dd, $^2J_{C\text{-}F}$ = 29.2, 28.0 Hz); IR (KBr): 3061, 2939, 1686, 1619, 1487, 1441, 1380, 1122, 1020, 897, 822, 744; HRMS (ESI) calcd. for $C_{18}H_{15}F_3O_3$ $[M+H]^+$ 337.1052, found: 337.1049

6-chloro-3-[2,2-difluoro-2-(4-methoxy-phenyl)-ethyl]-chroman-4-one (3gb)

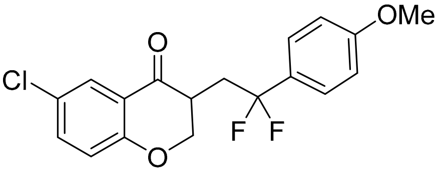

39.5 mg, 56% yield, white solid; mp 116.1- 116.5 ºC; ^{1}H NMR (500 MHz, $CDCl_3$) δ 7.83 (d, *J* = 2.7 Hz, 1H), 7.45 (d, *J* = 8.8 Hz, 2H), 7.41 (dd, *J* = 8.8, 2.7 Hz, 1H), 6.93 (dd, *J* = 8.8, 5.7 Hz, 3H), 4.74 (dd, *J* = 11.6, 5.2 Hz, 1H), 4.23 (t, *J* = 11.7 Hz, 1H), 3.83 (s, 3H), 3.15 - 3.00 (m, 2H), 2.12 - 1.98 (m, 1H); ^{19}F NMR (470 MHz, $CDCl_3$) δ -87.93 (ddd, *J* = 245.8, 24.0, 8.1 Hz), -95.83 (ddd, *J* = 245.5, 22.0, 16.1 Hz); ^{13}C NMR (125 MHz, $CDCl_3$) δ 191.1, 160.9, 160.1, 135.9, 128.7 (t, $^2J_{C\text{-}F}$= 26.9 Hz), 127.0, 126.8, 126.4 (t, $^3J_{C\text{-}F}$ = 6.1 Hz), 122.7 (t, $^1J_{C\text{-}F}$ = 242.3 Hz), 121.1, 119.6, 114.0, 70.6, 55.4, 41.2, 34.2 (t, $^2J_{C\text{-}F}$ = 28.9 Hz); IR (KBr): 3087, 2921, 1698, 1610, 1571, 1515, 1472, 1381, 1104, 1022, 889, 826, 776, 426; HRMS (ESI) calcd. for $C_{18}H_{15}ClF_2O_3$ $[M+H]^+$ 353.0756, found: 353.0753

6-bromo-3-(2,2-difluoro-2-(4-methoxyphenyl)ethyl)chroman-4-one (3hb)

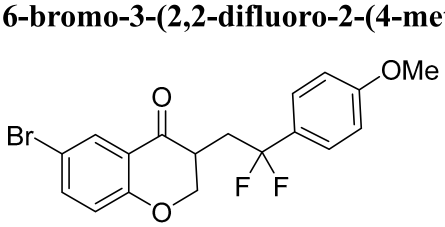

43.7 mg, 55% yield, white solid; mp 125.4 - 125.7 ºC; ^{1}H NMR (500 MHz, $CDCl_3$) δ 7.97 (d, *J* = 2.5 Hz, 1H), 7.54 (dd, *J* = 8.8, 2.6 Hz, 1H), 7.45 (d, *J* = 8.8 Hz, 2H), 6.94 (d, *J* = 8.8 Hz, 2H), 6.87 (d, *J* = 8.8 Hz, 1H), 4.75 (dd, *J* = 11.5, 5.1 Hz, 1H), 4.23 (t, *J* = 11.7 Hz, 1H), 3.83 (s, 3H), 3.18 - 2.96 (m, 2H), 2.19 - 1.91 (m, 1H); ^{19}F NMR (470

MHz, $CDCl_3$) δ -87.93 (ddd, J = 245.7, 24.0, 8.0 Hz), -95.83 (ddd, J = 245.5, 22.0, 16.0 Hz); ^{13}C NMR (125 MHz, $CDCl_3$) δ 191.0, 160.9, 160.5, 138.7, 129.9, 128.6 (t, $^2J_{C\text{-}F}$= 26.9 Hz), 126.4 (t, $^3J_{C\text{-}F}$ = 5.9 Hz), 122.7 (t, $^1J_{C\text{-}F}$ = 242.2 Hz), 121.6, 119.9, 114.1, 114.0, 70.6, 55.4, 41.1, 34.2 (t, $^2J_{C\text{-}F}$ = 28.7 Hz); IR (KBr): 3083, 2908, 1697, 1608, 1515, 1470, 1103, 1023, 889, 824, 777, 675; HRMS (ESI) calcd. for $C_{18}H_{15}BrF_2O_3$ $[M+H]^+$ 397.0251, found: 397.0253

7-chloro-3-(2,2-difluoro-2-(4-methoxyphenyl)ethyl)chroman-4-one (3ib)

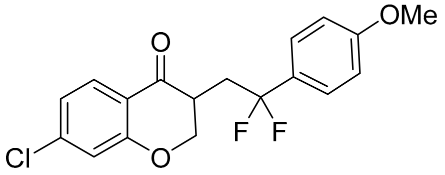

45.2 mg, 64% yield, white solid; mp 116.3 - 116.6 ºC; ^{1}H NMR (500 MHz, $CDCl_3$) δ 7.80 (d, J = 8.9 Hz, 1H), 7.45 (d, J = 8.7 Hz, 2H), 7.00 - 6.97 (m, 2H), 6.94 (d, J = 8.7 Hz, 2H), 4.75 (dd, J = 11.7, 5.2 Hz, 1H), 4.24 (t, J = 11.8 Hz, 1H), 3.82 (s, 3H), 3.17 - 2.97 (m, 2H), 2.15 - 1.95 (m, 1H); ^{19}F NMR (470 MHz, $CDCl_3$) δ -87.90 (ddd, J = 245.6, 23.9, 8.2 Hz), -95.75 (ddd, J = 245.6, 21.9, 16.2 Hz); ^{13}C NMR (125MHz, $CDCl_3$) δ 191.2, 162.0, 160.9, 141.9, 128.8, 128.6 (t, $^2J_{C\text{-}F}$ = 26.8 Hz), 126.4 (t, $^3J_{C\text{-}F}$ = 6.1 Hz), 122.8 (t, $^1J_{C\text{-}F}$ = 242.0 Hz), 122.3, 118.9, 118.0, 114.0, 70.8, 55.4, 41.2, 34.2 (t, $^2J_{C\text{-}F}$ = 28.6 Hz); IR (KBr): 3012, 2949, 1674, 1605, 1516, 1468, 1379, 1069, 1025, 934, 834, 806, 705, 579, 448; HRMS (ESI) calcd. for $C_{18}H_{15}ClF_2O_3$ $[M+H]^+$ 353.0756, found: 353.0755

8-chloro-3-(2,2-difluoro-2-(4-methoxyphenyl)ethyl)chroman-4-one (3jb)

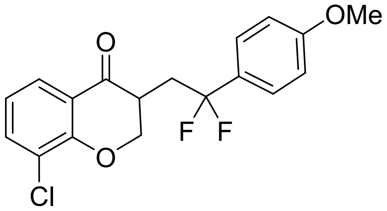

24.7 mg, 35% yield, light yellow solid; mp 76.1 - 76.8 ºC; ^{1}H NMR (500 MHz, $CDCl_3$) δ 7.80 (dd, J = 8.0, 1.6 Hz, 1H), 7.56 (dd, J = 8.0, 1.6 Hz, 1H), 7.46 (d, J = 9.2 Hz, 2H), 7.00 - 6.91 (m, 3H), 4.89 (dd, J = 11.2, 5.2 Hz, 1H), 4.31 (t, J = 11.6 Hz, 1H), 3.83 (s, 3H), 3.23 - 2.95 (m, 2H), 2.15 - 2.01 (m, 1H); ^{19}F NMR (470 MHz, $CDCl_3$) δ -87.87 (ddd, J = 245.9, 23.0, 8.2 Hz), -95.55 (ddd, J = 246.0, 21.4, 16.8 Hz); ^{13}C NMR (125 MHz, $CDCl_3$) δ 191.3, 160.9, 157.1, 136.1, 128.5 (t, $^2J_{C\text{-}F}$ = 26.8 Hz), 126.4 (t, $^3J_{C\text{-}F}$ = 6.1 Hz), 126.1, 122.7 (t, $^1J_{C\text{-}F}$ = 242.4 Hz), 122.6, 121.6, 121.6, 114.0, 71.1, 55.4, 41.2, 34.1 (t, $^2J_{C\text{-}F}$ = 28.9 Hz); IR (KBr): 3074, 2924, 1682, 1608, 1450, 1515, 1374, 1115, 1023, 835, 767, 730, 595, 432; HRMS (ESI) calcd. for $C_{18}H_{15}ClF_2O_3$ $[M+H]^+$ 353.0756, found: 353.0759

3-(2,2-difluoro-2-(4-methoxyphenyl)ethyl)-6-iodochroman-4-one (3kb)

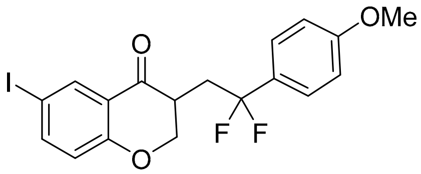

35.5 mg, 40% yield, white solid; mp 130.1 -131.1 ºC; ^{1}H NMR (500 MHz, $CDCl_3$) δ 8.15 (d, J = 2.2 Hz, 1H), 7.70 (dd, J = 8.7, 2.3 Hz, 1H), 7.44 (d, J = 8.7 Hz, 2H), 6.93 (d, J = 8.7 Hz, 2H), 6.74 (d, J = 8.7 Hz, 1H), 4.74 (dd, J = 11.5, 5.1 Hz, 1H), 4.22 (t, J = 11.7 Hz, 1H), 3.82 (s, 3H), 3.14 - 2.99 (m, 2H), 2.10 - 1.99 (m, 2H); ^{19}F NMR (470MHz, $CDCl_3$) δ -87.86 (ddd, J = 245.8, 24.0, 7.8 Hz), -95.71 (ddd, J = 245.5, 21.8, 16.2 Hz); ^{13}C NMR (125 MHz, $CDCl_3$) δ 190.8, 161.2, 160.9, 144.3, 136.1, 128.6 (t, $^{2}J_{C\text{-}F}$ = 26.7 Hz), 126.4 (t, $^{3}J_{C\text{-}F}$ = 6.1 Hz), 122.7 (t, $^{1}J_{C\text{-}F}$ = 242.3 Hz), 122.2, 120.2, 114.0, 83.8, 70.5, 55.4, 41.0, 34.2 (t, $^{2}J_{C\text{-}F}$ = 28.7 Hz); IR (KBr): 3078, 2902, 1692, 1592, 1514, 1101, 1027, 893, 823, 781, 571; HRMS (ESI) calcd. for $C_{18}H_{15}F_2IO_3$ $[M+H]^+$ 445.0122, found: 445.0124

6,8-dibromo-3-(2,2-difluoro-2-(4-methoxyphenyl)ethyl)chroman-4-one (3lb)

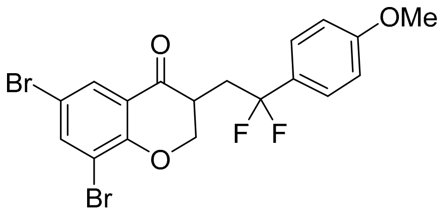

17.1 mg, 18% yield, white solid; mp 125.2 - 125.4 ºC; ^{1}H NMR (500MHz, $CDCl_3$) δ 7.94 (d, J = 2.4 Hz, 1H), 7.83 (d, J = 2.4 Hz, 1H), 7.44 (d, J = 8.8 Hz, 2H), 6.94 (d, J = 8.7 Hz, 2H), 4.90 (dd, J = 11.5, 5.4 Hz, 1H), 4.30 (t, J = 12.0 Hz, 1H), 3.83 (s, 3H), 3.21 - 2.97 (m, 2H), 2.20 - 1.96 (m, 1H); ^{19}F NMR (470 MHz, $CDCl_3$) δ -87.96 (ddd, J = 25.8, 23.4, 7.7 Hz), -95.58 (ddd, J = 245.8, 21.4, 16.2 Hz); ^{13}C NMR (125 MHz, $CDCl_3$) δ 190.2, 160.9, 157.1, 141.1, 129.4, 128.4 (t, $^{2}J_{C\text{-}F}$ = 26.6 Hz), 126.4 (t, $^{3}J_{C\text{-}F}$ = 6.0 Hz), 122.6 (t, $^{1}J_{C\text{-}F}$ = 241.7 Hz), 122.2, 114.0, 112.6, 71.2, 55.4, 40.8, 34.1 (t, $^{2}J_{C\text{-}F}$ = 28.9 Hz); IR (KBr): 3063, 2939, 1685, 1614, 1515, 1438, 1114, 1022, 894, 837, 806, 767, 576; HRMS (ESI) calca. For $C_{18}H_{14}Br_2F_2O_3$ $[M+H]^+$ 474.9356, found: 474.9353

3-(2,2-difluoro-2-(4-methoxyphenyl)ethyl)-3-methylchroman-4-one (3mb)

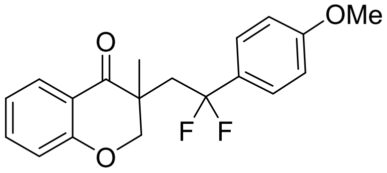

27.3 mg, 41% yield, light yellow oil; ^{1}H NMR (500 MHz, $CDCl_3$) δ 7.89 (dd, J = 7.9, 1.8 Hz, 1H), 7.48 (ddd, J = 8.4, 7.2, 1.8 Hz, 1H), 7.41 (d, J = 8.9 Hz, 2H), 7.03 (ddd, J = 8.0, 7.2, 1.0 Hz, 1H), 6.98 (dd, J = 8.4, 0.7 Hz, 1H), 6.90 (d, J = 8.8 Hz, 2H), 4.58 (d,

J = 11.6 Hz, 1H), 4.35 (d, J = 11.7 Hz, 1H), 3.82 (s, 3H), 2.74 (ddd, J = 29.9, 15.7, 7.9 Hz, 1H), 2.42 (ddd, J = 26.9, 15.7, 11.5 Hz, 1H), 1.32 (s, 3H); ^{19}F NMR (470 MHz, $CDCl_3$) δ -89.01 (ddd, J = 243.5, 29.9, 11.5 Hz), -90.24 (ddd, J = 243.3, 26.7, 7.9 Hz); ^{13}C NMR (125 MHz, $CDCl_3$) δ 195.2, 161.0, 160.6, 135.7, 130.0 (t, $^2J_{C-F}$ = 26.7 Hz), 128.1, 126.3 (t, $^3J_{C-F}$ = 6.3 Hz), 122.9 (t, $^1J_{C-F}$ =244.2 Hz), 121.6, 119.3, 117.7, 113.7, 74.5, 55.3, 44.1, 41.7 (t, $^2J_{C-F}$ = 27.6 Hz), 19.8; IR (KBr): 3072, 2932, 1690, 1611, 1515, 1467, 1363, 1111, 1031, 829, 764; HRMS (ESI) calcd. for $C_{19}H_{18}F_2O_3$ $[M+H]^+$ 333.1302, found: 333.1306

2-(2,2-difluoro-2-(*p*-tolyl)ethyl)-2,3-dihydro-1H-inden-1-one (3na)

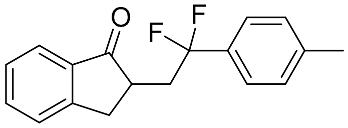

21.1mg, 37% yield, white solid; mp 122.8 - 123.3 ºC; ^{1}H NMR (500 MHz, $CDCl_3$) δ 7.75 (d, J = 7.7 Hz, 1H), 7.43 (d, J = 8.1 Hz, 2H), 7.38 (d, J = 7.1 Hz, 1H), 7.24 (d, J = 7.9 Hz, 2H), 3.44 (dd, J = 17.5, 8.1 Hz, 1H), 3.08 - 2.92 (m, 2H), 2.90 - 2.81 (m, 1H), 2.39 (s, 3H), 2.14 (dddd, J = 20.7, 14.9, 11.5, 8.0 Hz, 1H); ^{19}F NMR (470 MHz, $CDCl_3$) δ -90.63 (ddd, J = 244.7, 21.8, 7.9 Hz), -96.99 (ddd, J = 244.6, 20.3, 16.9 Hz); ^{13}C NMR (125 MHz, $CDCl_3$) δ 206.6, 153.7, 140.0, 136.0, 135.0, 134.1 (t, $^2J_{C-F}$ = 26.4 Hz), 129.2, 127.5, 126.5, 124.8 (t, $^3J_{C-F}$ = 6.2 Hz), 124.0, 123.2 (t, $^1J_{C-F}$ = 242.7 Hz), 42.9, 40.2 (t, $^2J_{C-F}$ = 27.8 Hz), 33.9, 21.3; IR (KBr): 3054, 2924, 1711, 1597, 1460, 1080, 1021, 849, 811, 760; HRMS (ESI) calcd. for $C_{18}H_{16}F_2O$ $[M+H]^+$ 287.1247, found: 287.1243

1,1,2,2-tetrafluoro-1,2-di-p-tolylethane (4)[4]

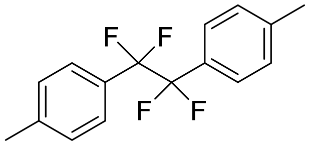

13.6 mg, 16% yield, white solid; mp 130.0 - 131.0 ºC; ^{1}H NMR (500 MHz, $CDCl_3$) δ 7.35 (d, J = 8.2 Hz, 4H), 7.22 (d, J = 8.1 Hz, 4H), 2.40 (s, 6H); ^{19}F NMR (470 MHz, $CDCl_3$) δ -111.34 (s). IR (KBr): 3050, 1450, 1261, 1140,1078, 929, 875, 750, 696

6. Single crystal X-ray analysis of 3aq′

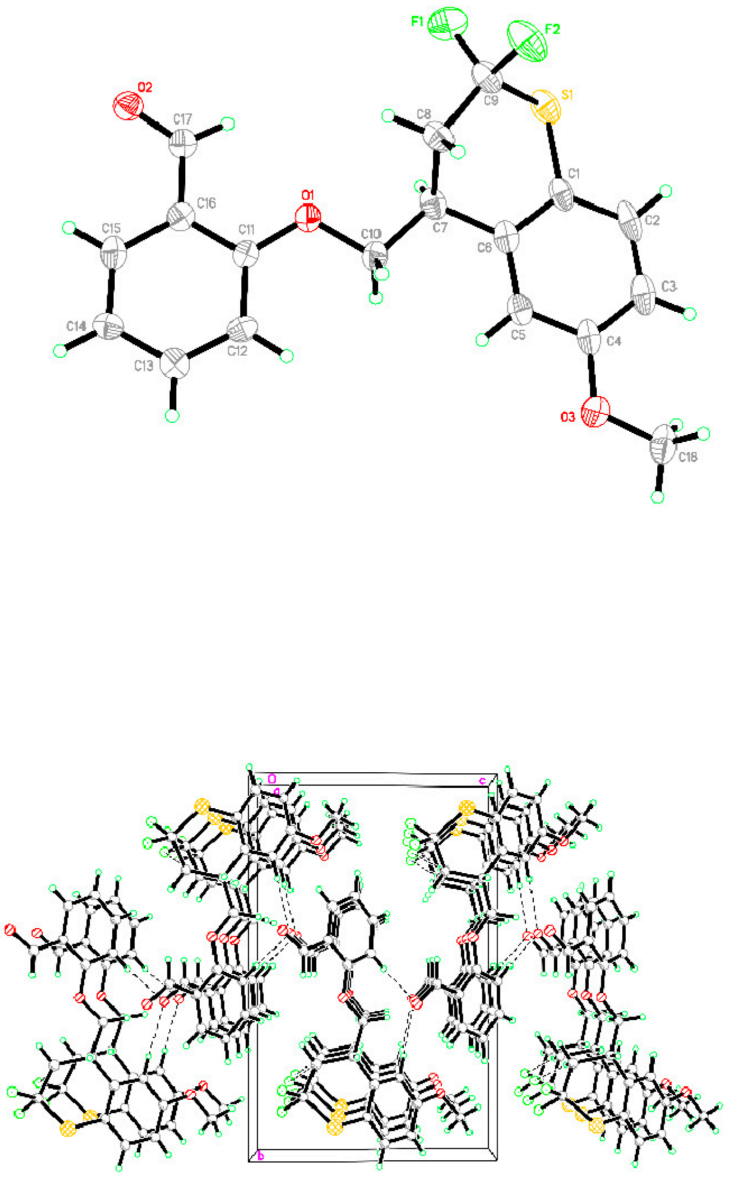

Figure S1. Crystal structure of **3aq′** (gray for carbon atoms, green for fluorine atom, yellow for sulfur atom and red for oxygen atom).

Table S3 Sample and crystal data for **3aq′**

Chemical formula	$C_{18}H_{16}F_2O_3S$
Formula weight	350.37 g/mol
Temperature	150(2) K
Wavelength	1.54178 Å
Crystal size	0.020 × 0.040 × 0.100 mm
Crystal system	monoclinic

Space group	P 1 c 1		
Unit cell dimensions	a = 4.8627(4) Å	α	= 90°
	b = 16.0653(12) Å	β	= 93.788(6)°
	c = 10.2801(7) Å	γ	= 90°
Volume	801.33(10) Å3		
Z	2		
Density (calculated)	1.452 g/cm^3		
Absorption coefficient	2.123 mm^{-1}		
F (000)	364		

Table S4. Bond lengths (Å) for **3aq′**

S1-C1	1.756(11)	S1-C9	1.809(11)
F1-C9	1.360(12)	F2-C9	1.370(11)
O1-C11	1.353(10)	O1-C10	1.442(10)
O2-C17	1.238(11)	O3-C4	1.386(14)
O3-C18	1.433(12)	C1-C6	1.380(12)
C1-C2	1.391(14)	C2-C3	1.426(17)
C2-H2	0.950000	C3-C4	1.354(14)
C3-H3	0.950000	C4-C5	1.404(13)
C5-C6	1.394(14)	C5-H5	0.950000
C6-C7	1.512(12)	C7-C10	1.518(12)
C7-C8	1.533(13)	C7-H7	1.000000
C8-C9	1.519(13)	C8-H8A	0.990000
C8-H8B	0.990000	C10-H10A	0.990000
C10-H10B	0.990000	C11-C16	1.404(12)
C11-C12	1.415(12)	C12-C13	1.368(12)
C12-H12	0.950000	C13-C14	1.389(13)
C13-H13	0.950000	C14-C15	1.376(14)
C14-H14	0.950000	C15-C16	1.397(13)
C15-H15	0.950000	C16-C17	1.445(13)
C17-H17	0.950000	C18-H18A	0.980000
C18-H18B	0.980000	C18-H18C	0.980000

Table S5. Bond angles (°) for **3aq′**

C1-S1-C9	100.0(4)	C11-O1-C10	117.3(7)
C4-O3-C18	117.5(8)	C6-C1-C2	120.4(10)
C6-C1-S1	119.8(7)	C2-C1-S	119.8(7)
C1-C2-C3	120.4(8)	C1-C2-H2	119.800000
C3-C2-H2	119.800000	C4-C3-C2	118.7(10)
C4-C3-H3	120.700000	C2-C3-H3	120.700000
C3-C4-O3	124.7(9)	C3-C4-C5	120.9(11)
O3-C4-C5	114.4(7)	C6-C5-C4	120.7(8)
C6-C5-H5	119.700000	C4-C5-H5	119.700000

C1-C6-C5	119.0(8)	C1-C6-C7	118.3(9)
C5-C6-C7	122.7(8)	C6-C7-C10	113.6(8)
C6-C7-C8	108.0(7)	C10-C7-C8	111.6(7)
C6-C7-H7	107.800000	C10-C7-H7	107.800000
C8-C7-H7	107.800000	C9-C8-C7	114.1(8)
C9-C8-H8A	108.700000	C7-C8-H8A	108.700000
C9-C8-H8B	108.700000	C7-C8-H8B	108.700000
H8A-C8-H8B	107.600000	F1-C9-F2	104.0(8)
F1-C9-C8	111.6(8)	F2-C9-C8	108.3(8)
F1-C9-S1	106.0(7)	F2-C9-S1	110.6(7)
C8-C9-S1	115.8(7)	O1-C10-C7	106.2(7)
O1-C10-H10A	110.500000	C7-C10-H10A	110.500000
O1-C10-H10B	110.500000	C7-C10-H10B	110.500000
H10A-C10-H10B	108.700000	O1-C11-C16	117.2(7)
O1-C11-C12	123.4(7	C16-C11-C12	119.4(8)
C13-C12-C11	119.6(8)	C13-C12-H12	120.200000
C11-C12-H12	120.200000	C12-C13-C14	122.1(9
C12-C13-H13	119.000000	C14-C13-H13	119.000000
C15-C14-C13	118.1(8)	C15-C14-H14	120.900000
C13-C14-H14	120.900000	C14-C15-C16	122.4(9)
C14-C15-H15	118.800000	C16-C15-H15	118.800000
C15-C16-C11	118.5(8)	C15-C16-C17	120.5(8)
C11-C16-C17	121.0(8	O2-C17-C16	123.9(8)
O2-C17-H17	118.000000	C16-C17-H17	118.000000
O3-C18-H18A	109.500000	O3-C18-H18B	109.500000
H18A-C18-H18B	109.500000	O3-C18-H18C	109.500000
H18A-C18-H18C	109.500000	H18B-C18-H18	109.500000

7. Reference

1. Hu H.; Chen X. L.; Sun K.; Wang J. C.; Liu Y.; Liu H.; Yu B.; Sun Y. Q.; Qu L. B.; Zhao Y. F., *Org. Chem. Front.*, **2018**, *5*, 2925.
2. Jagdale A. R.; Park J. H.; Youn S. W., *J. Org. Chem.,* **2011**, *76*, 7204.
3. Mizuta, S.; Stenhagen, I. S. R.; O'Duill, M.; Wolstenhulme, J.; Kirjavainen, A. K.; Forsback, S. J.; Tredwell, M.; Sandford, G.; Moore, P. R.; Huiban, M.; Luthra, S. K.; Passchier, J.; Solin O.; Gouverneur, V., *Org. Lett.*, **2013**, *15*, 2648.
4. Zhou Y. L.; Xiong Z. M.; Qiu J.Y.; Kong L. C.; Zhu G. G., *Org. Chem. Front.*, **2019**, *6*, 1022.
5. Chang Y.; Tewari A.; Adi A.; Bae C., *Tetrahedron,* **2008**, *64,* 9837.

8. Copies of ^{1}H NMR, ^{19}F NMR and ^{13}C NMR Spectra of the componds 3

Figure S2. Copies of NMR Spectra of the componds 3

3-(2,2-difluoro-2-(p-tolyl)ethyl)chroman-4-one (3aa)

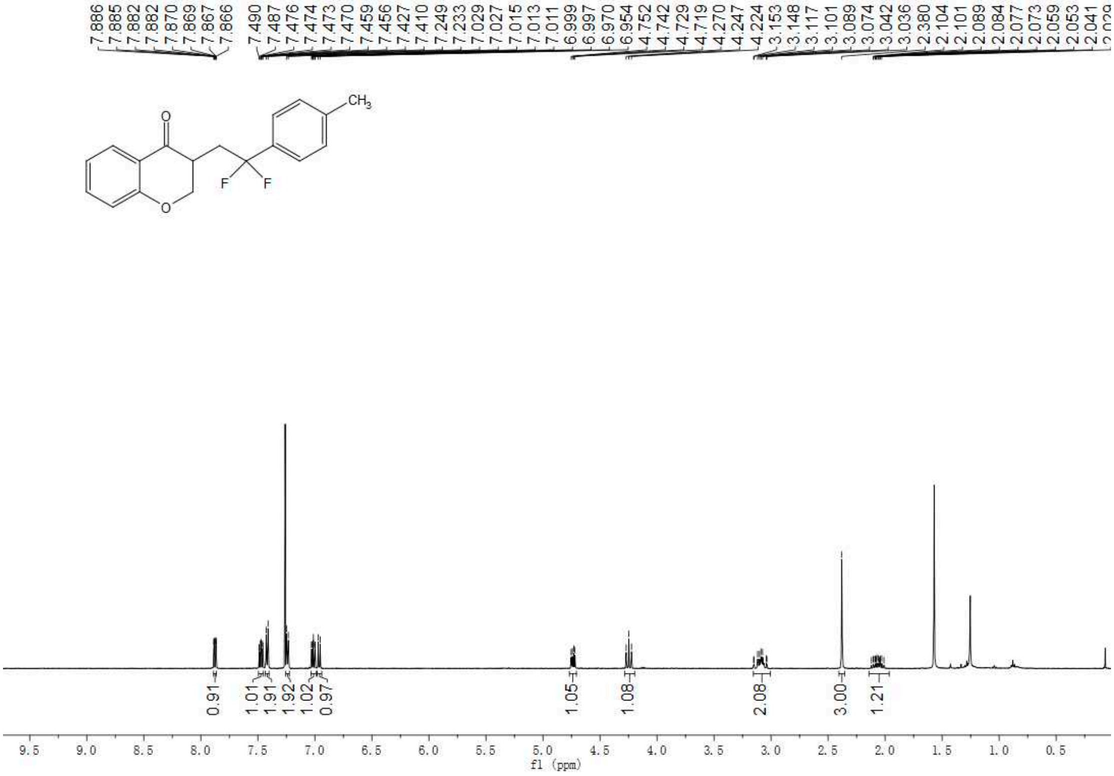

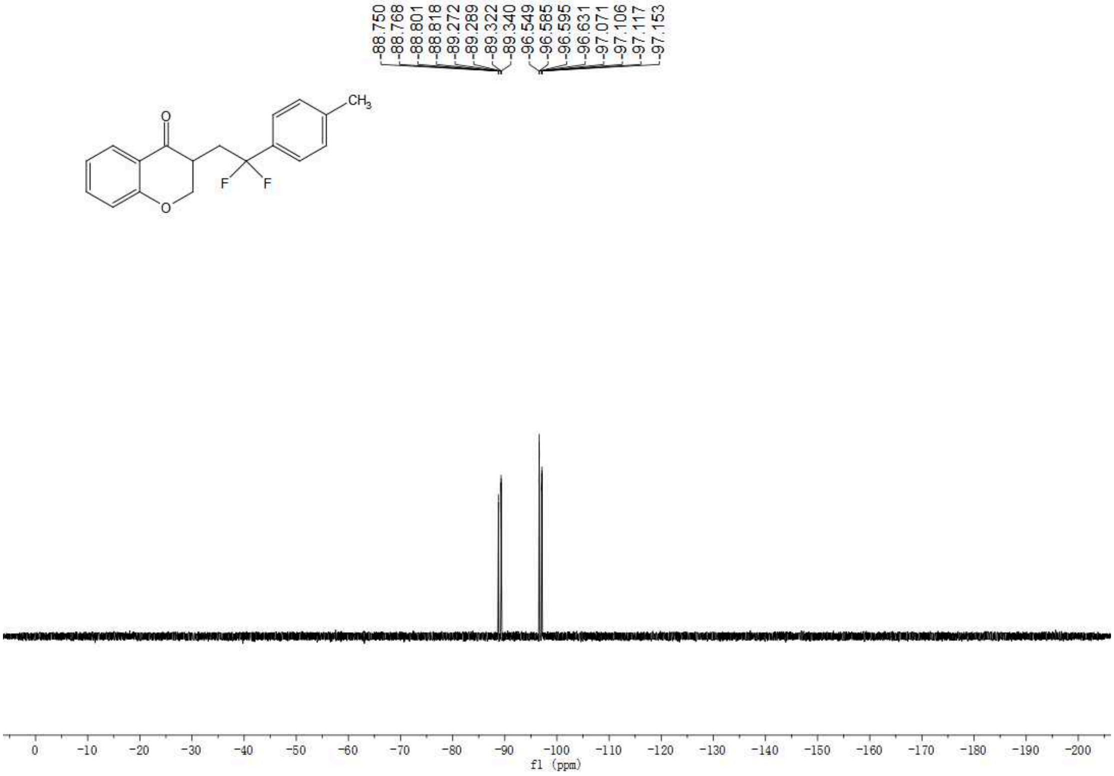

3-(2,2-difluoro-2-(4-methoxyphenyl)ethyl)chroman-4-one (3ab)

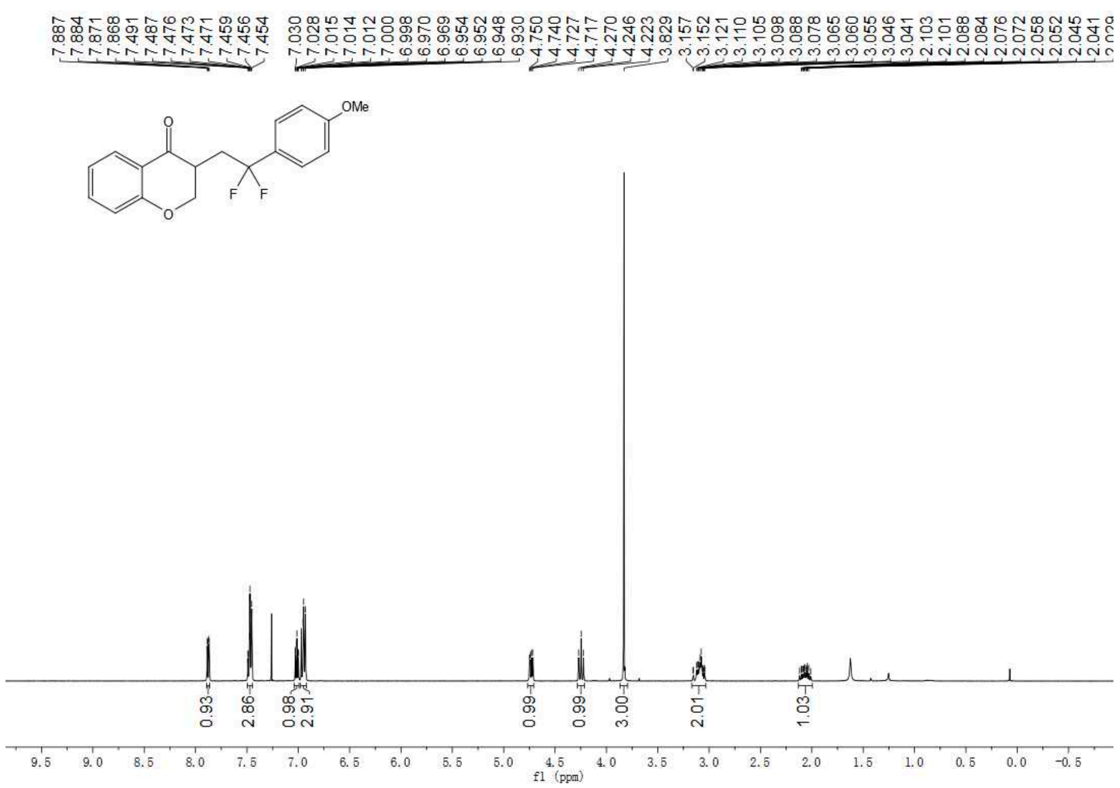

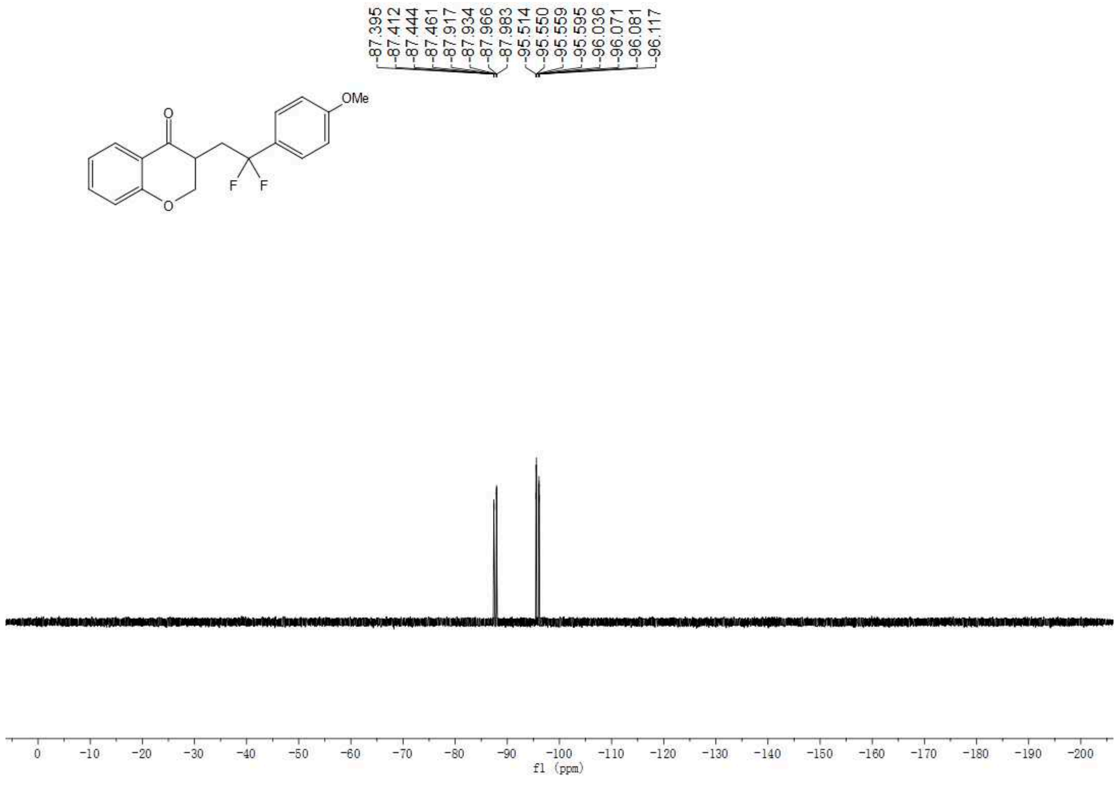

3-(2-(4-(tert-butyl)phenyl)-2,2-difluoroethyl)chroman-4-one (3ac)

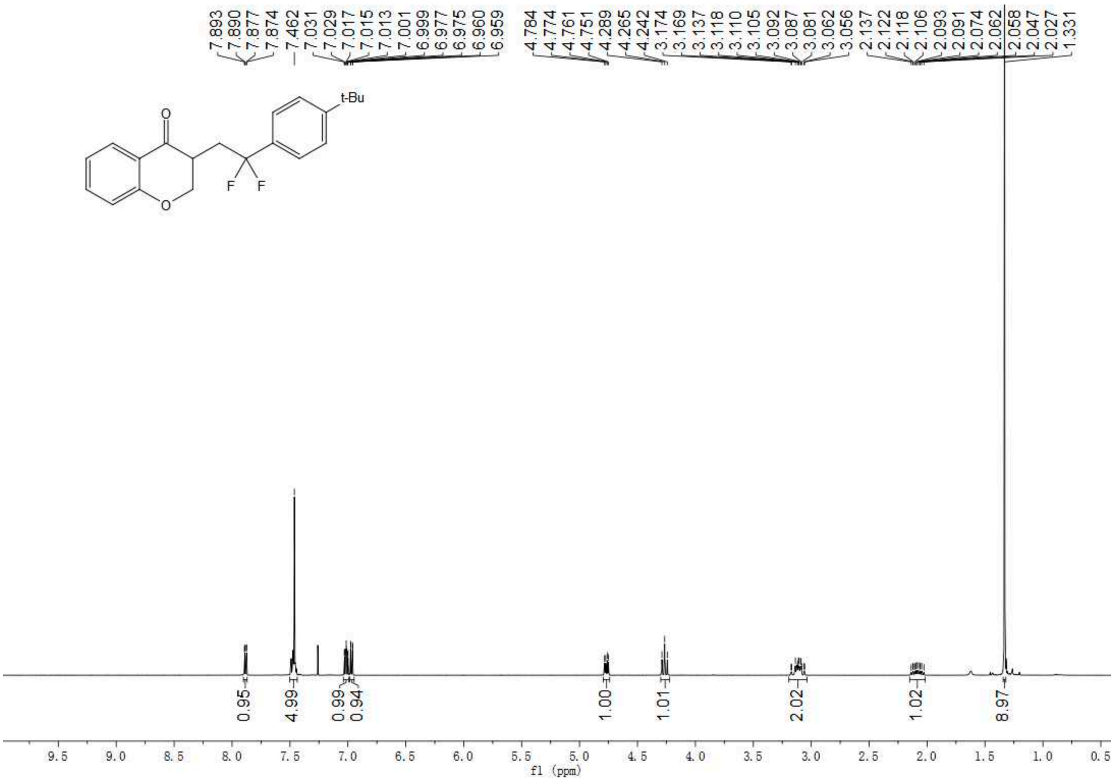

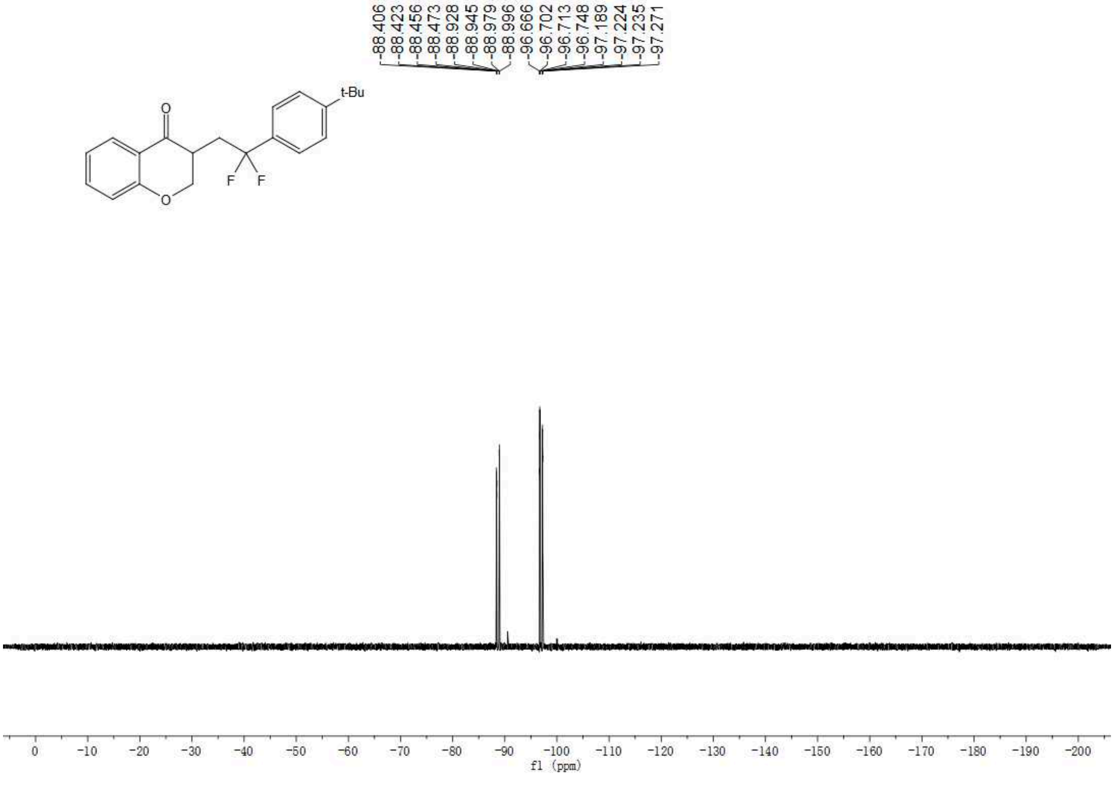

3-(2-([1,1'-biphenyl]-4-yl)-2,2-difluoroethyl)chroman-4-one (3ad)

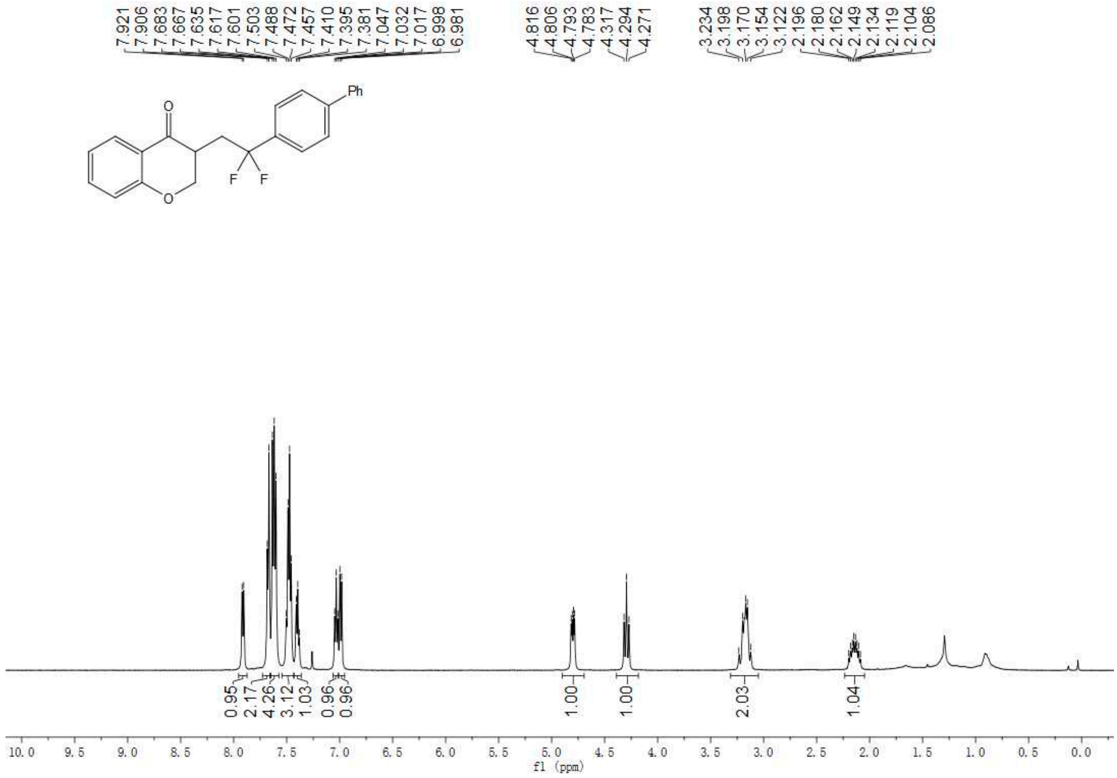

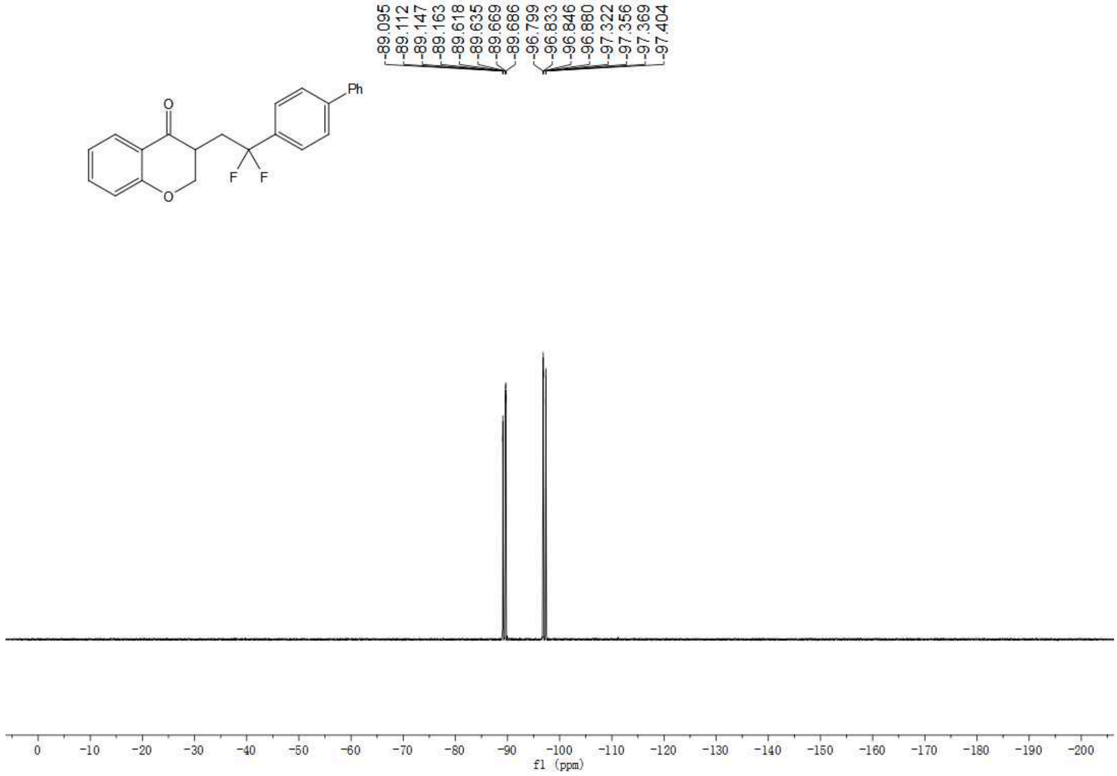

3-(2,2-difluoro-2-(m-tolyl)ethyl)chroman-4-one (3ae)

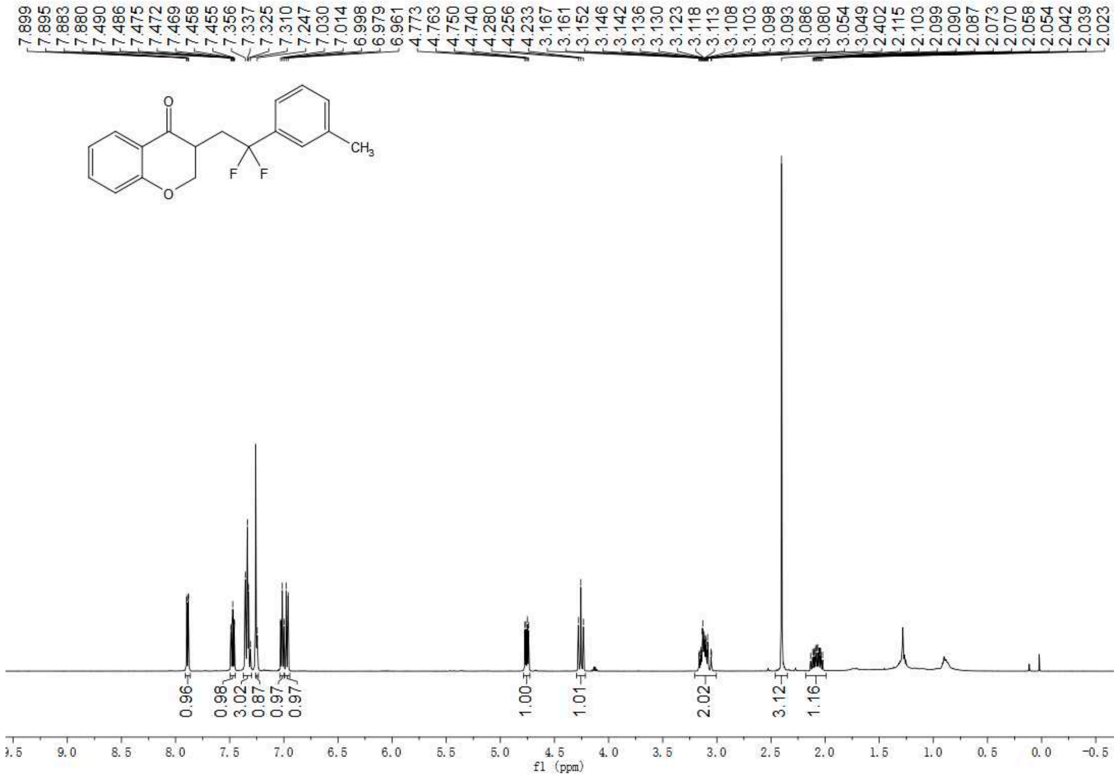

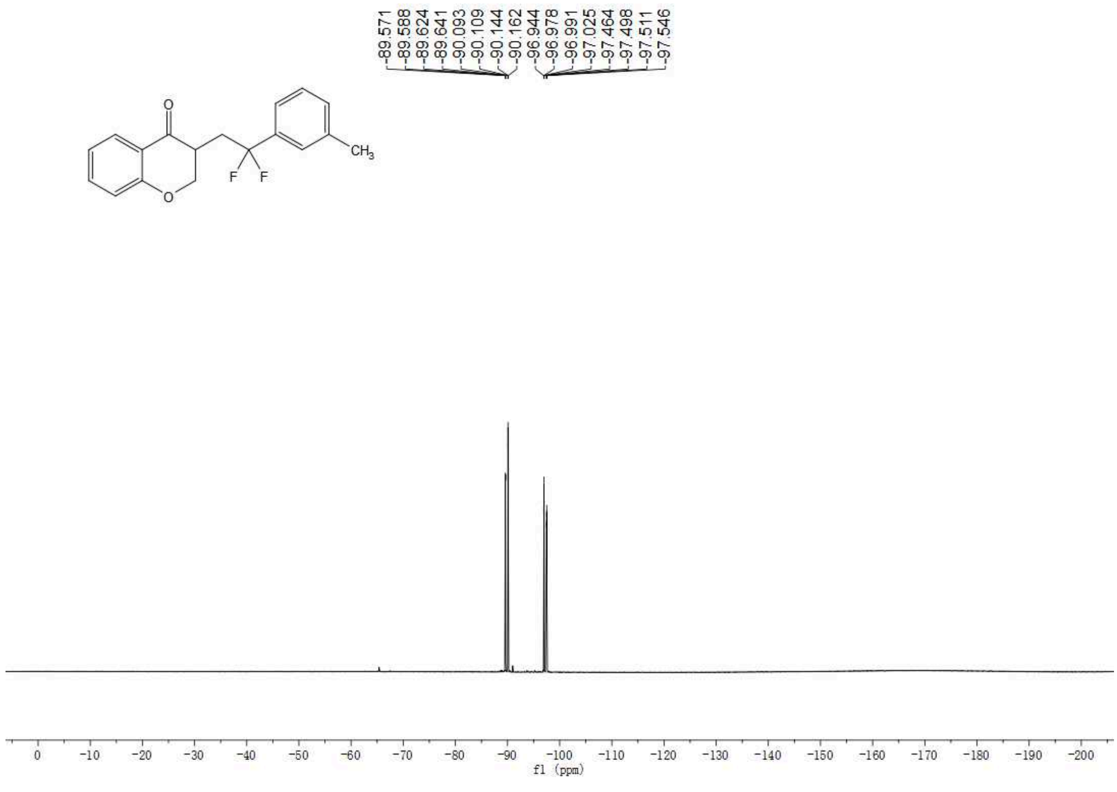

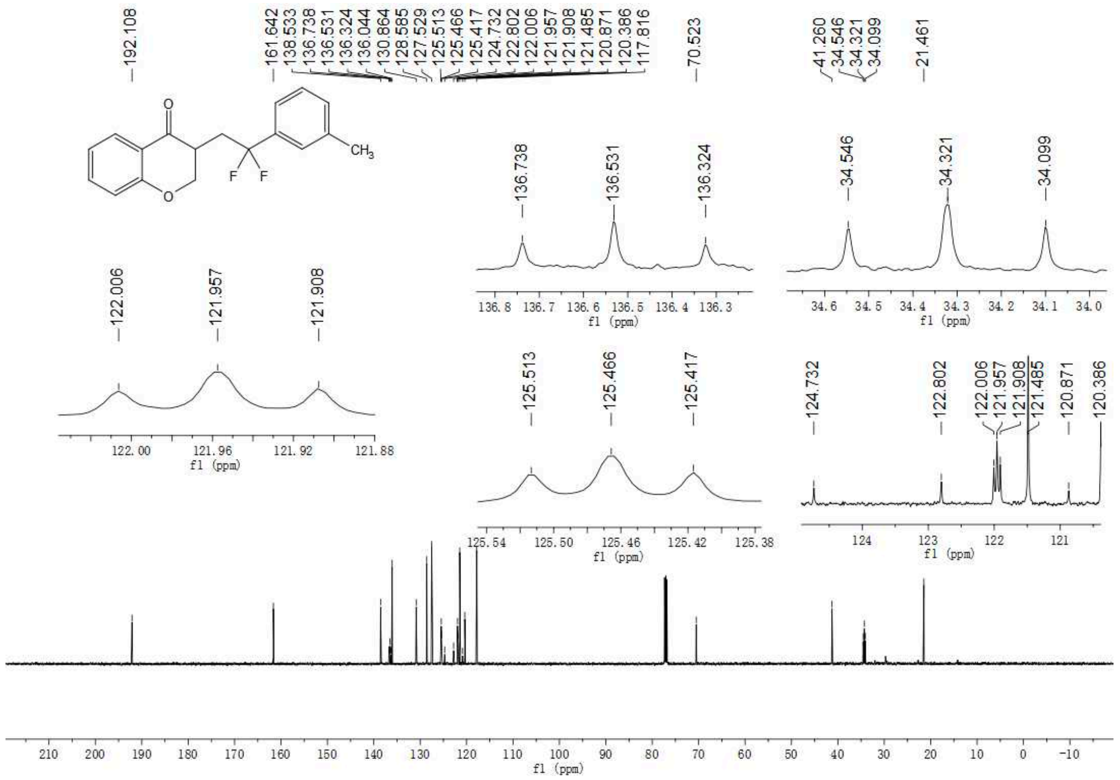

3-(2,2-difluoro-2-(o-tolyl)ethyl)chroman-4-one (3af)

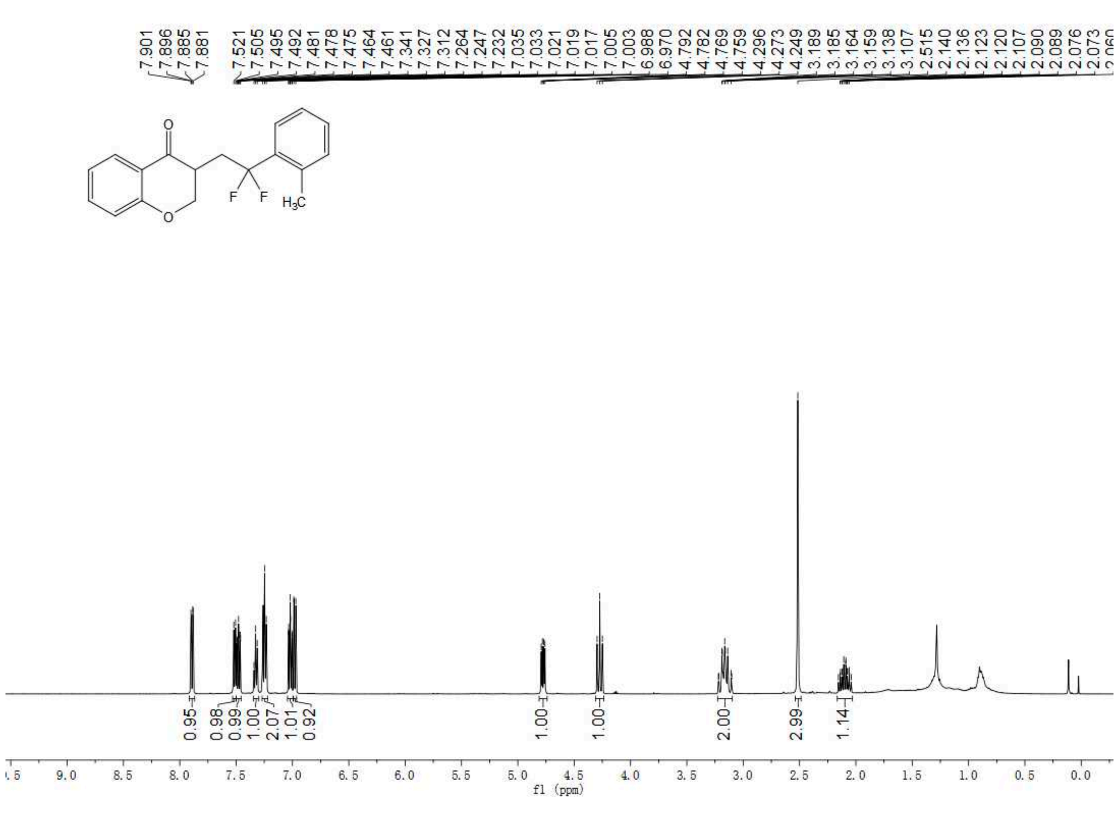

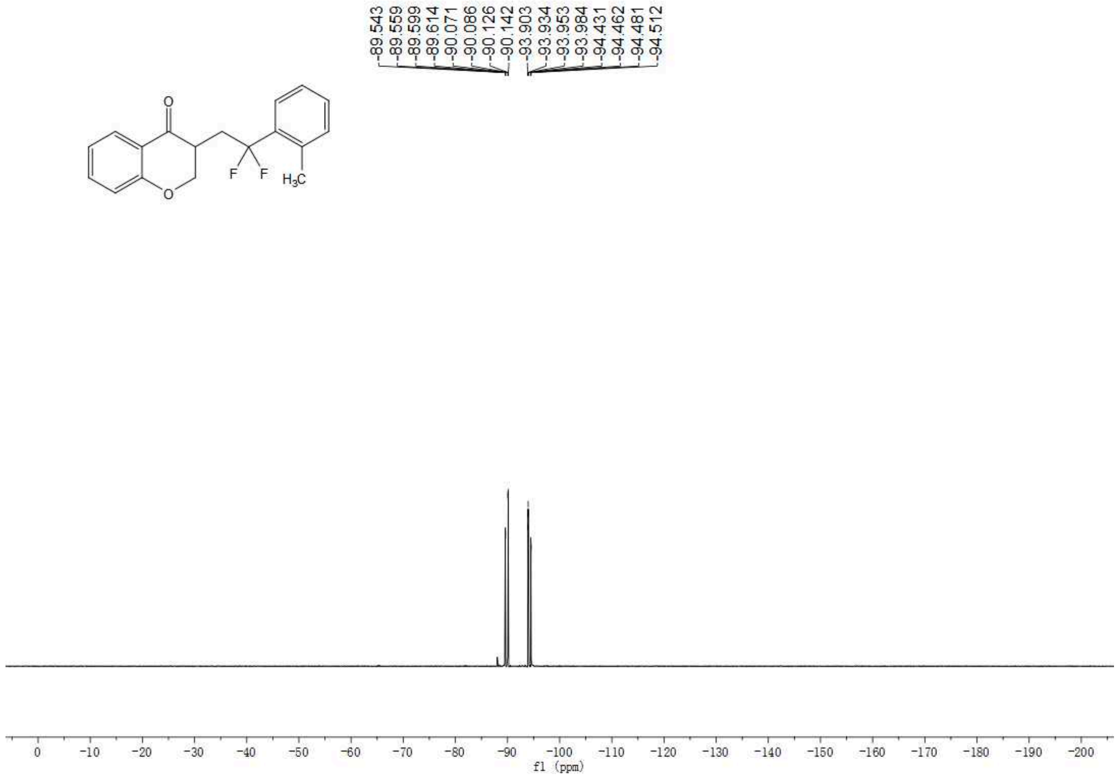

3-(2-(3,4-dimethylphenyl)-2,2-difluoroethyl)chroman-4-one (3ag)

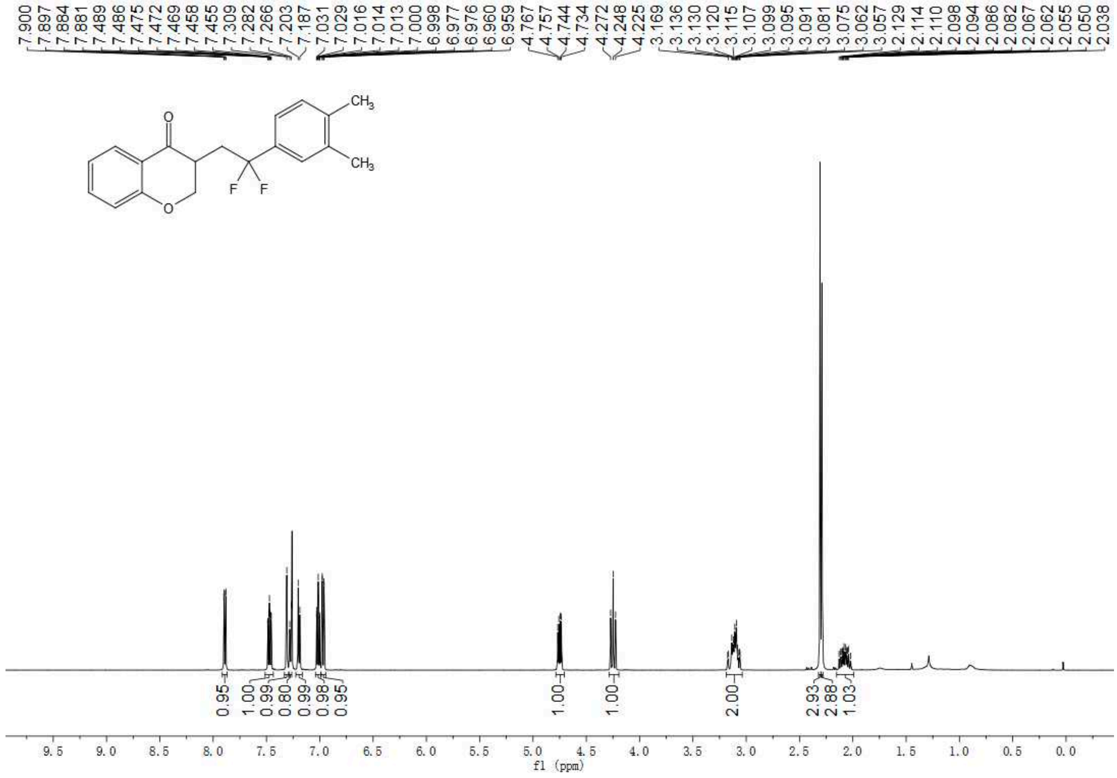

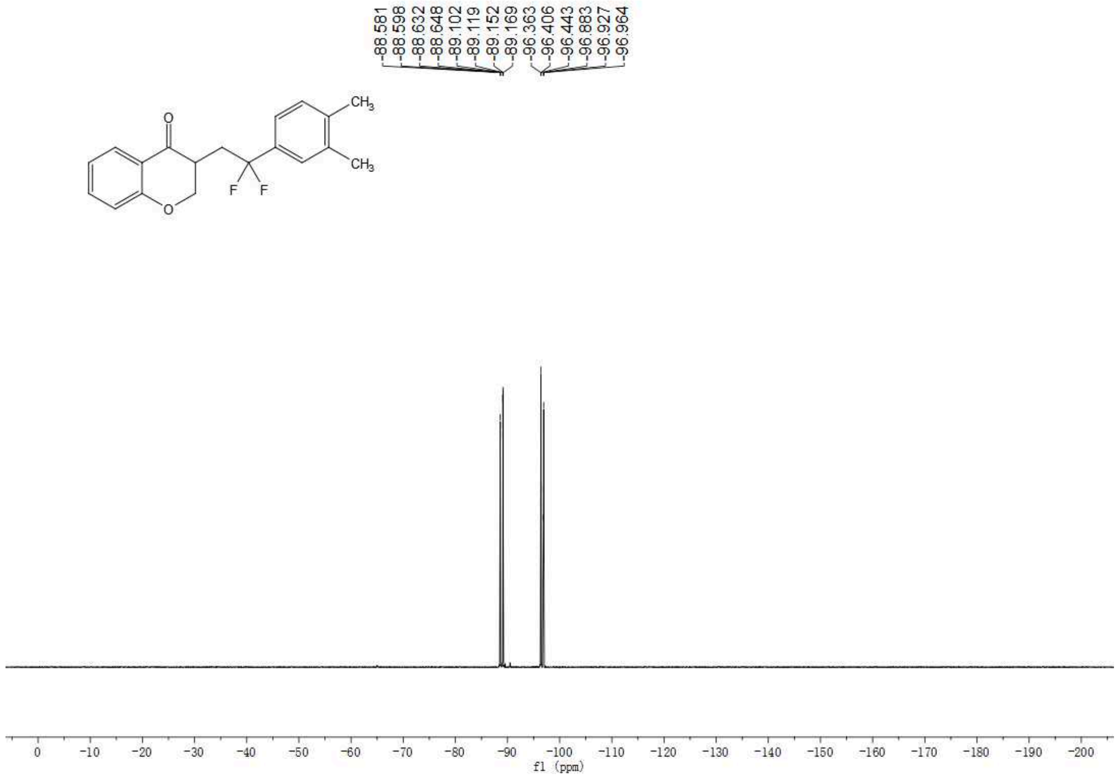
-88.581
-88.598
-88.632
-88.648
-89.102
-89.119
-89.152
-89.169
-96.363
-96.406
-96.443
-96.883
-96.927
-96.964
fl (ppm)

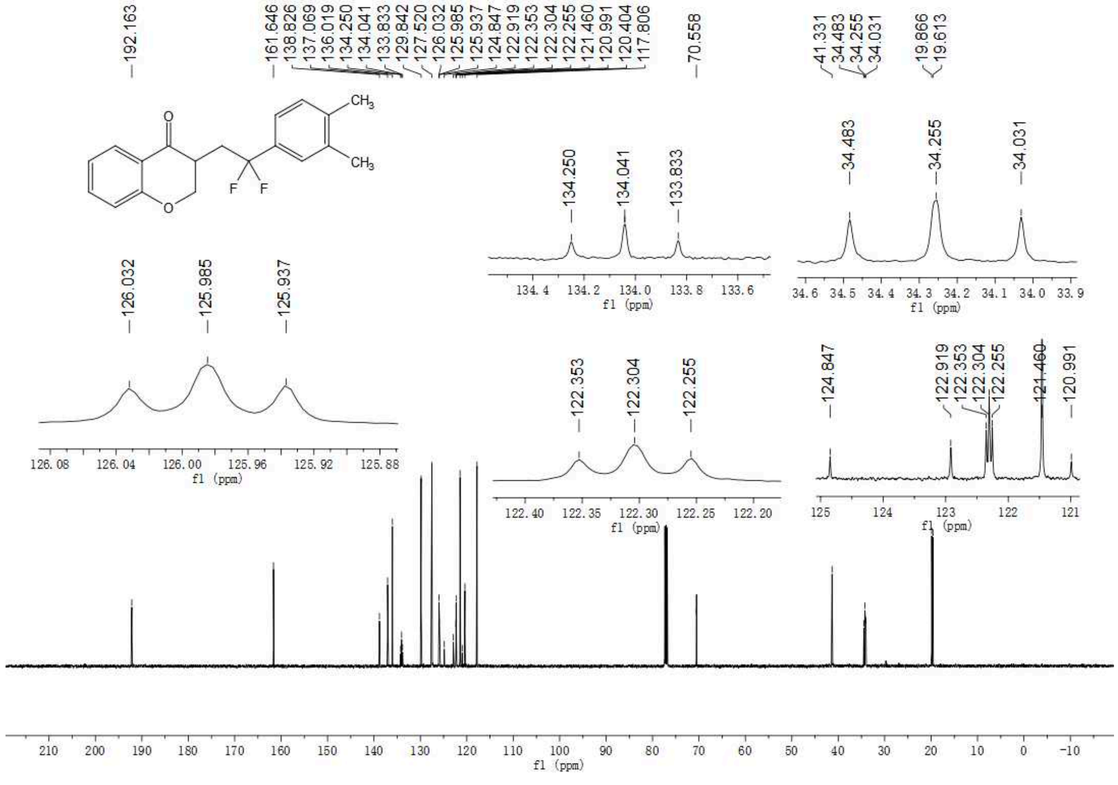
192.163
161.646
138.826
137.069
136.019
134.250
134.041
133.833
129.842
127.520
126.032
125.985
125.937
124.847
122.919
122.353
122.304
122.255
121.460
120.991
120.404
117.806
70.558
41.331
34.483
34.255
34.031
19.866
19.613
fl (ppm)

3-(2,2-difluoro-2-mesitylethyl)chroman-4-one (3ah)

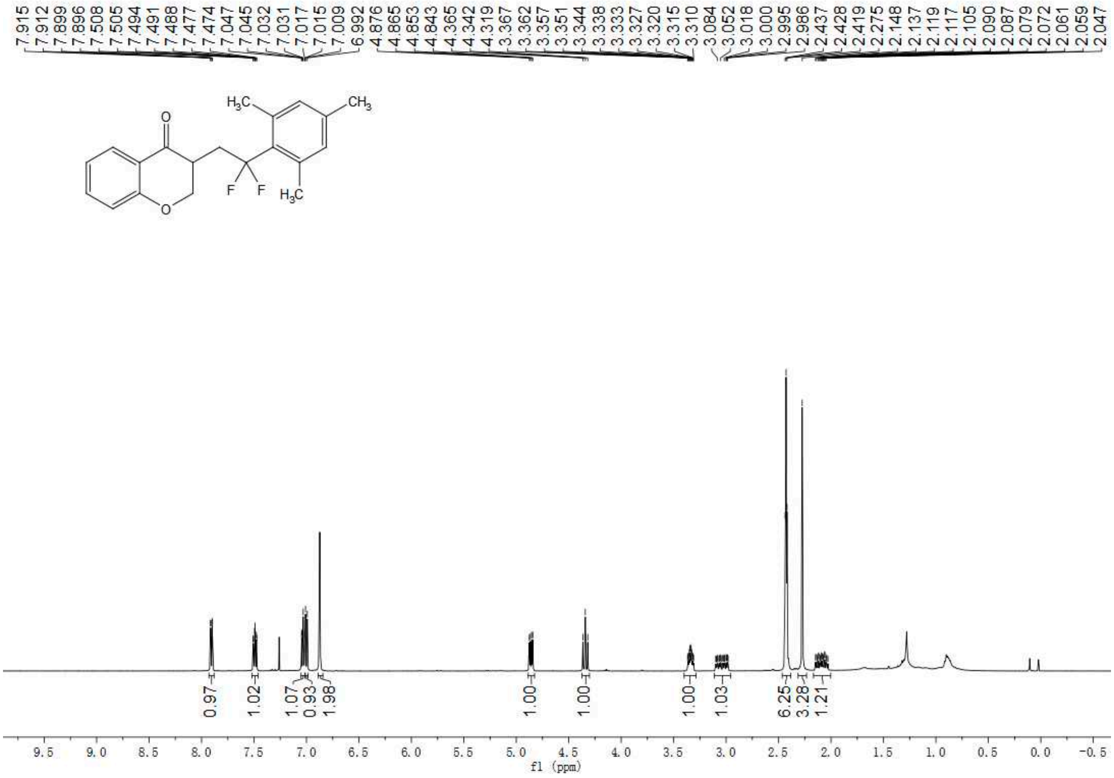

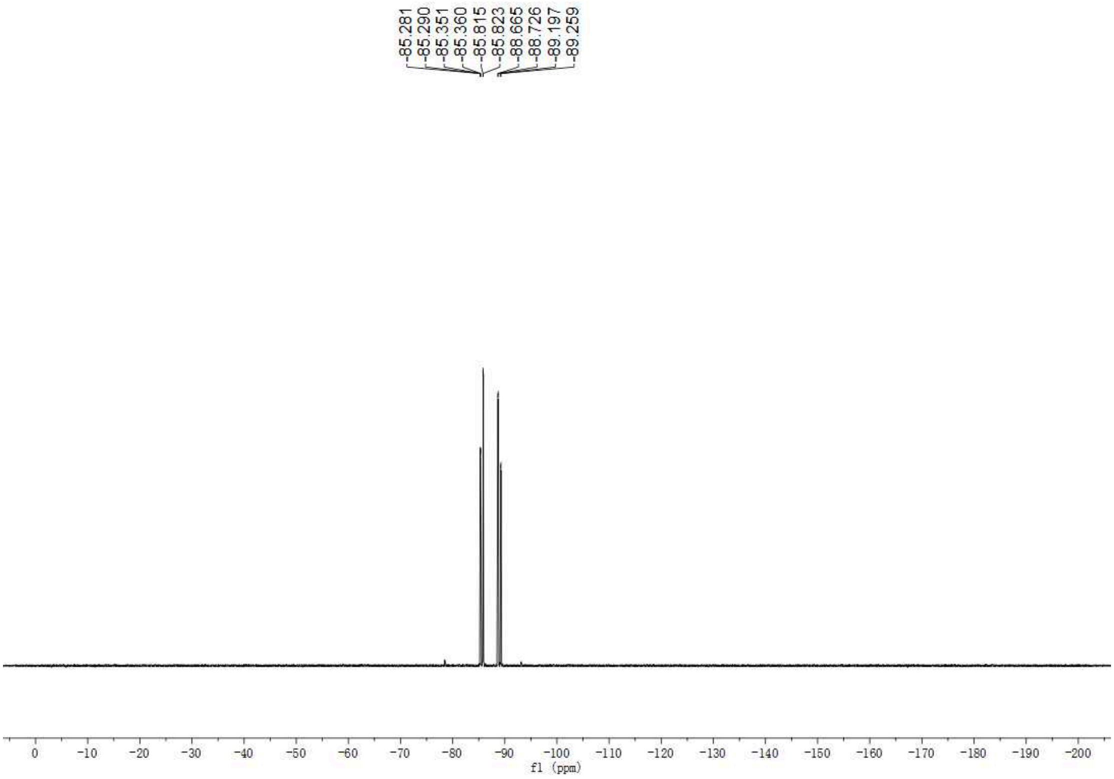

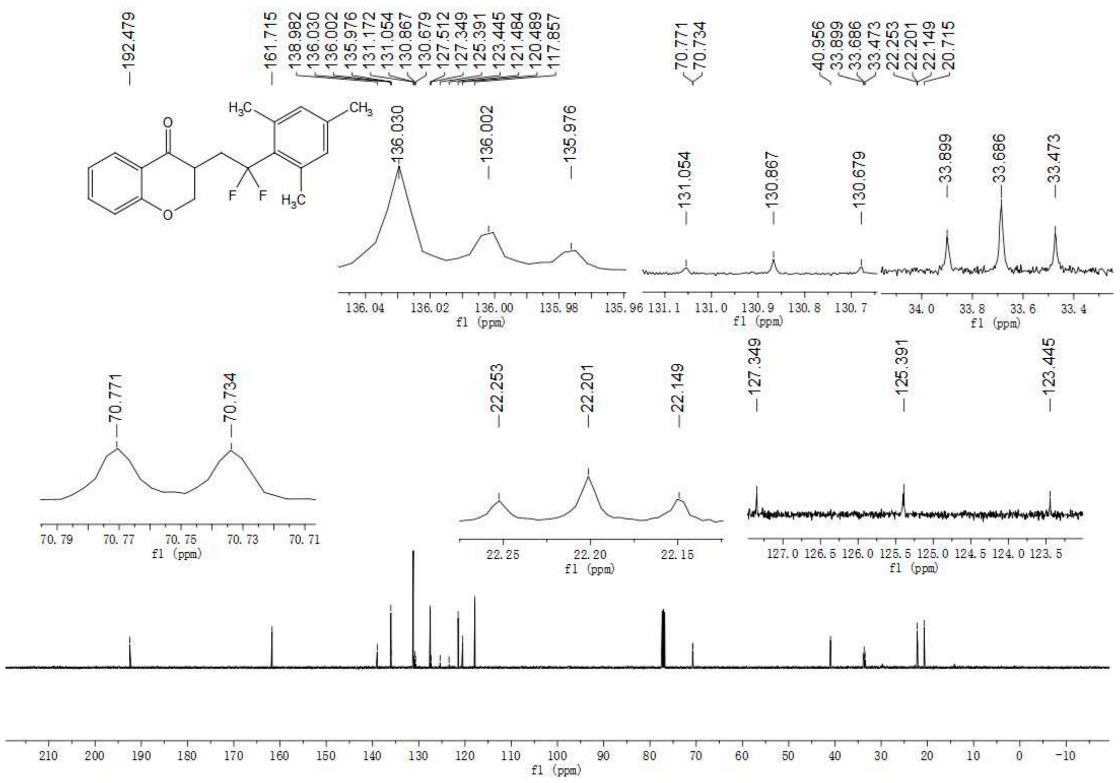

3-(2,2-difluoro-2-phenylethyl)chroman-4-one (3ai)

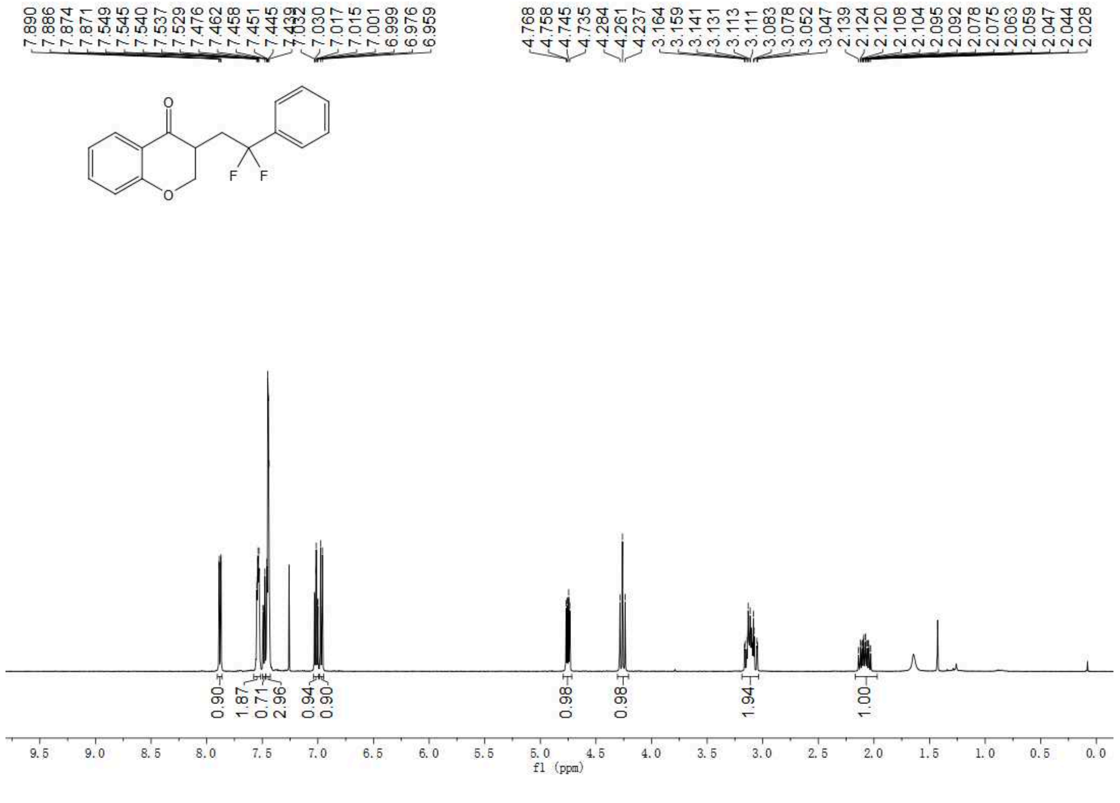

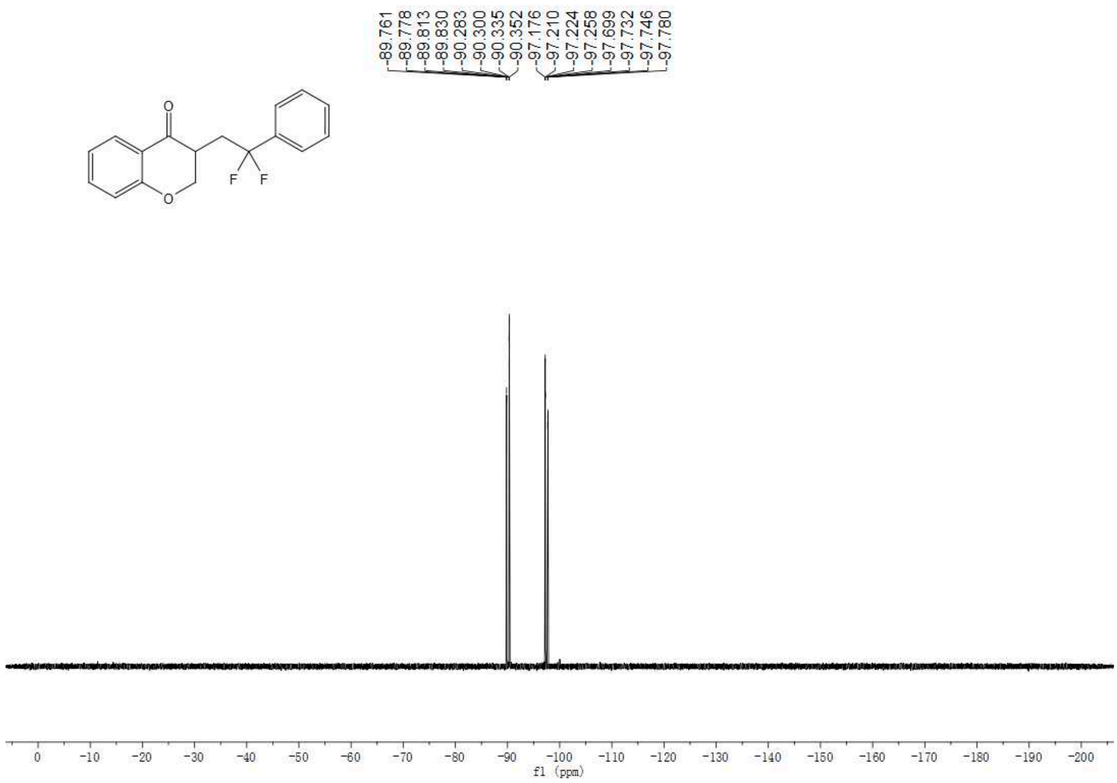

3-(2-(benzo[d][1,3]dioxol-5-yl)-2,2-difluoroethyl)chroman-4-one (3aj)

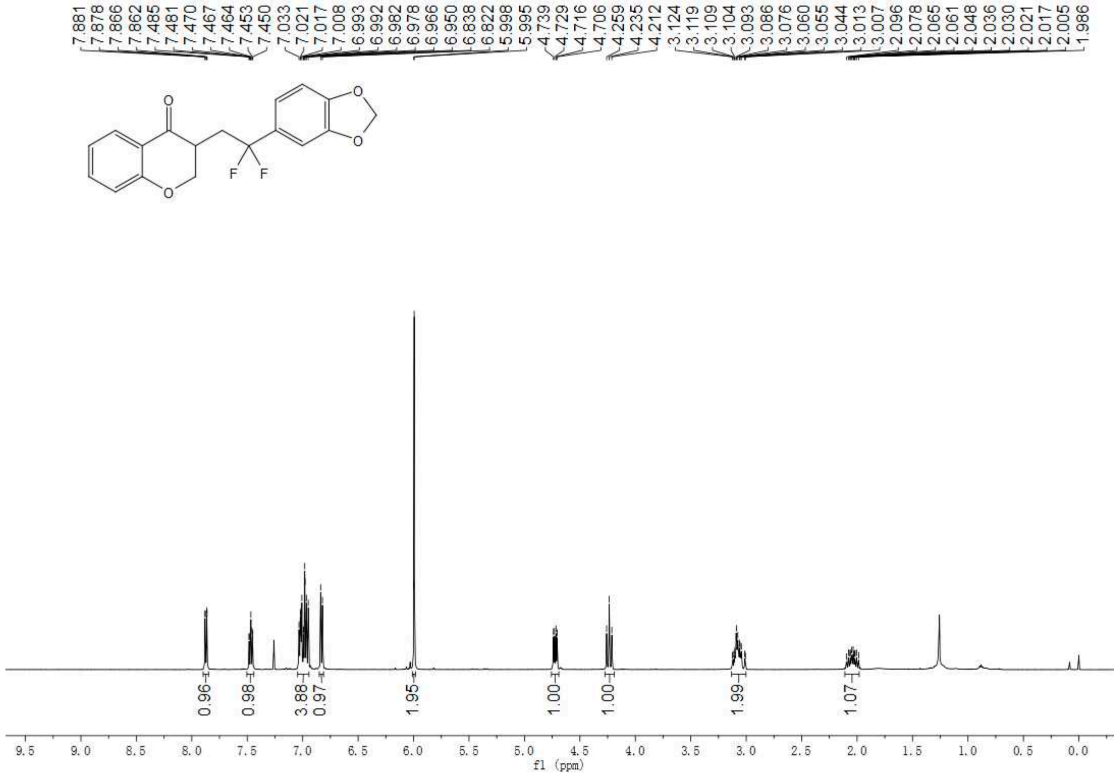

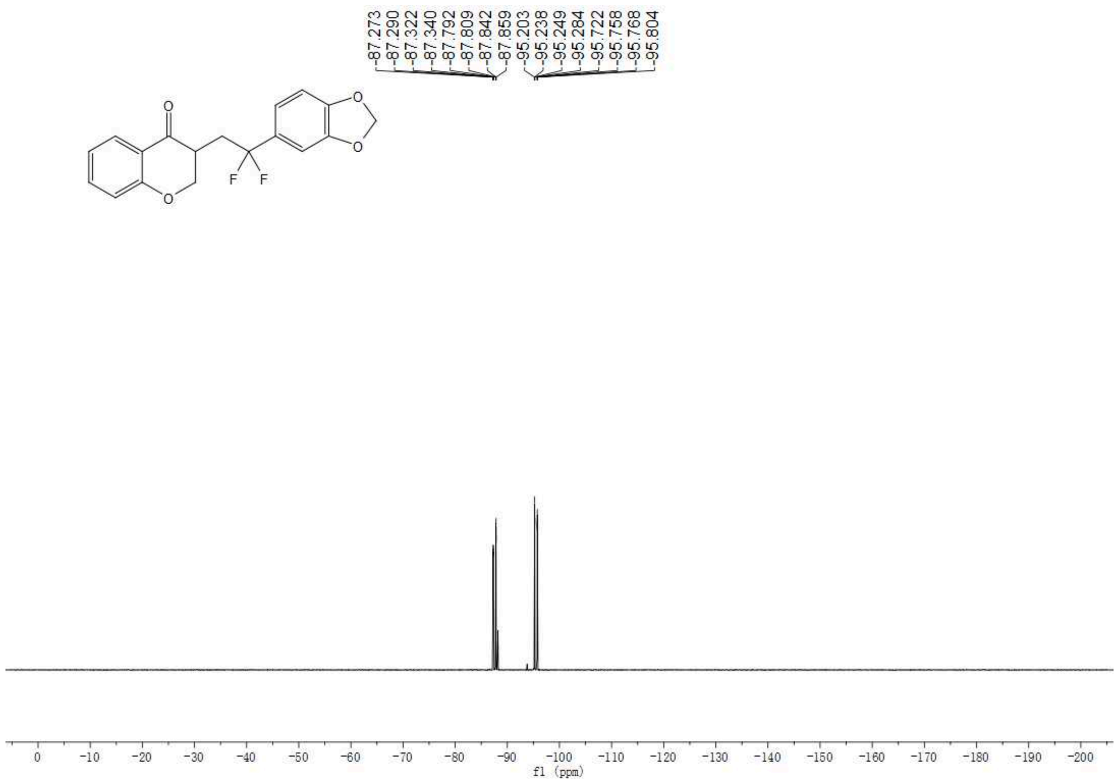
-87.273
-87.290
-87.322
-87.340
-87.792
-87.809
-87.842
-87.859
-95.203
-95.238
-95.249
-95.284
-95.722
-95.758
-95.768
-95.804
O
O
O
O
F F
f1 (ppm)

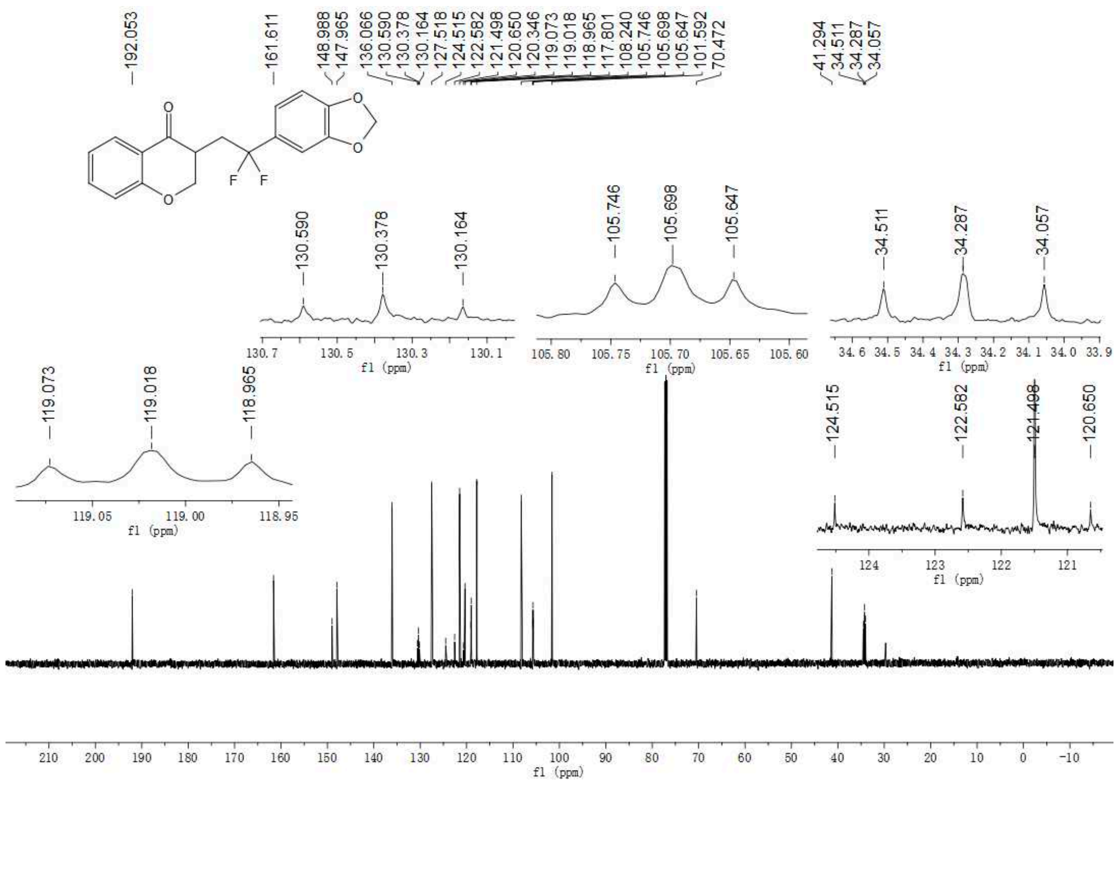
192.053
161.611
148.988
147.965
136.066
130.590
130.378
130.164
127.518
124.515
122.582
121.498
120.650
120.346
119.073
119.018
118.965
117.801
108.240
105.746
105.698
105.647
101.592
70.472
41.294
34.511
34.287
34.057
O
O
O
O
F F
f1 (ppm)

3-(2,2-difluoro-2-(4-fluorophenyl)ethyl)chroman-4-one (3ak)

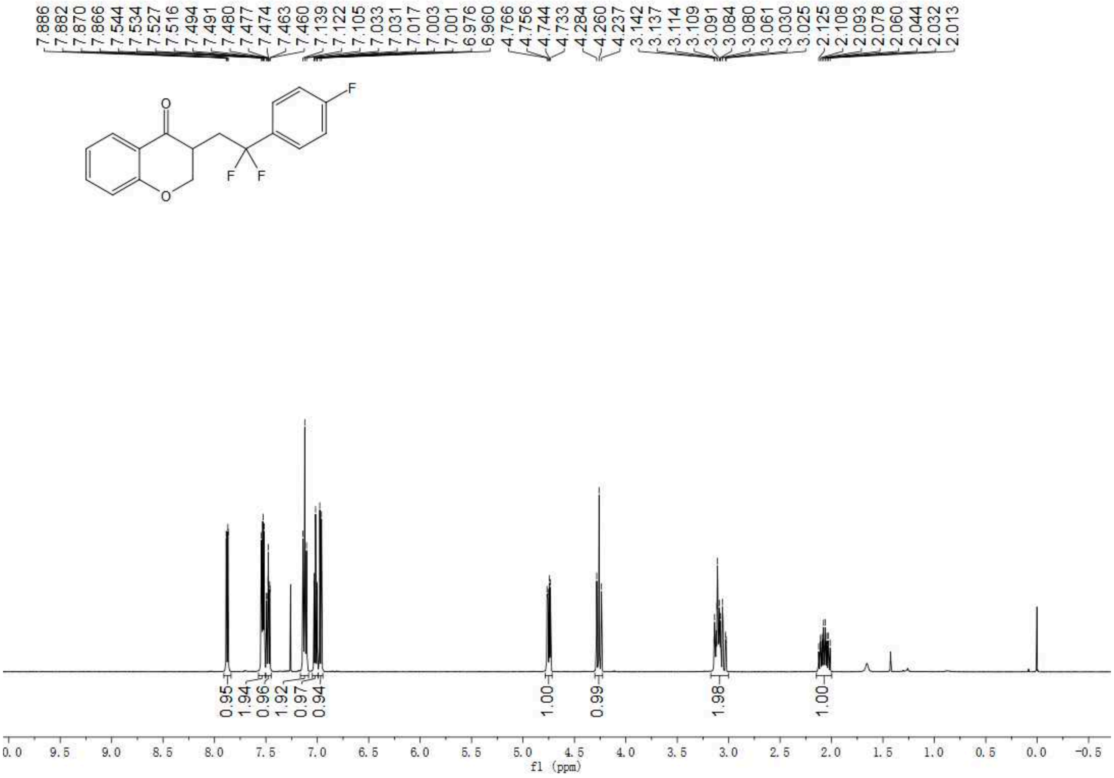

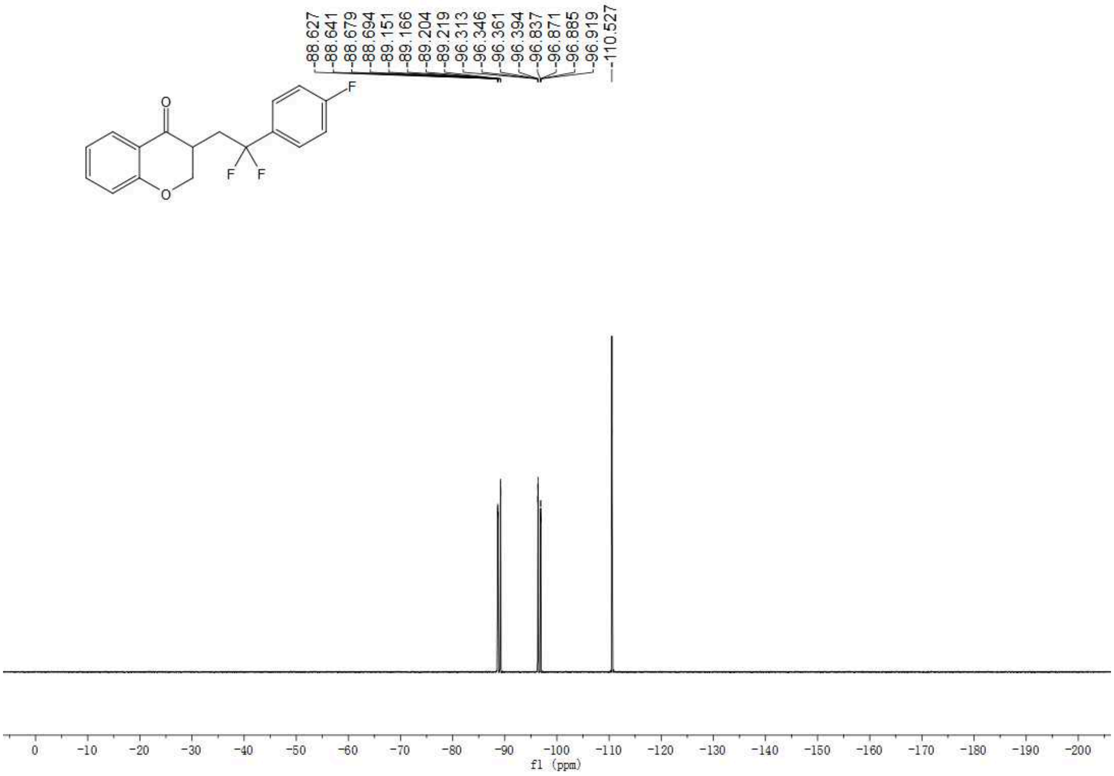

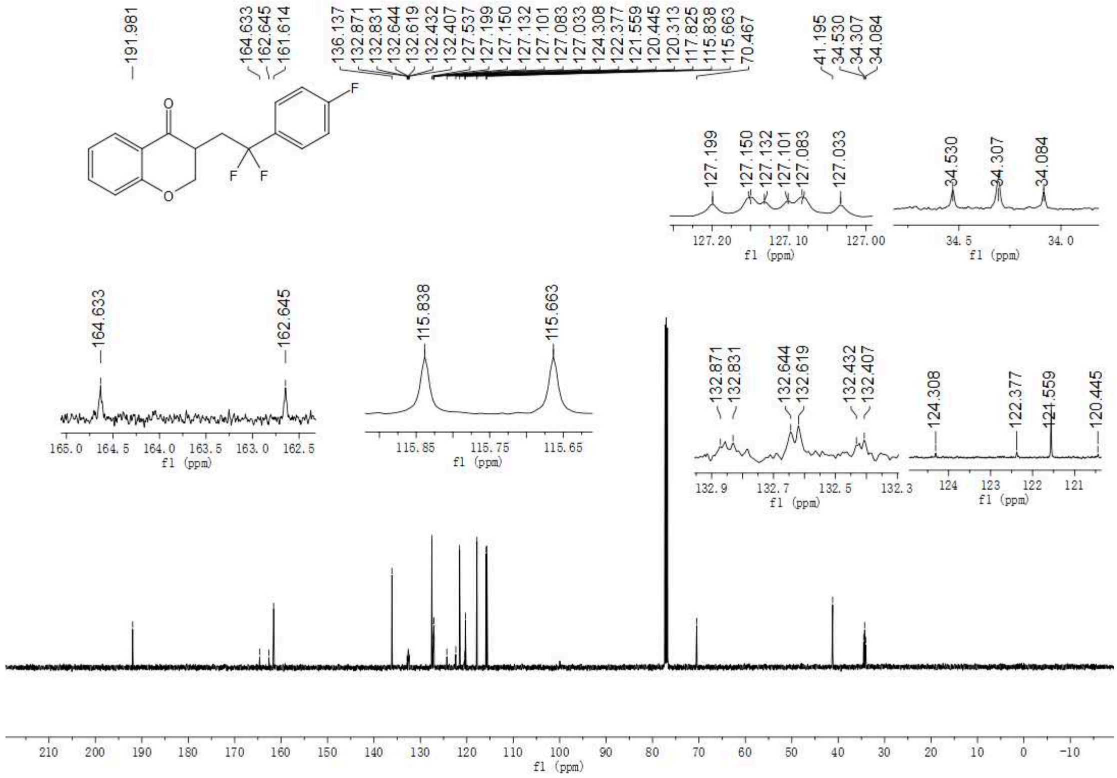

3-(2-(4-chlorophenyl)-2,2-difluoroethyl)chroman-4-one (3al)

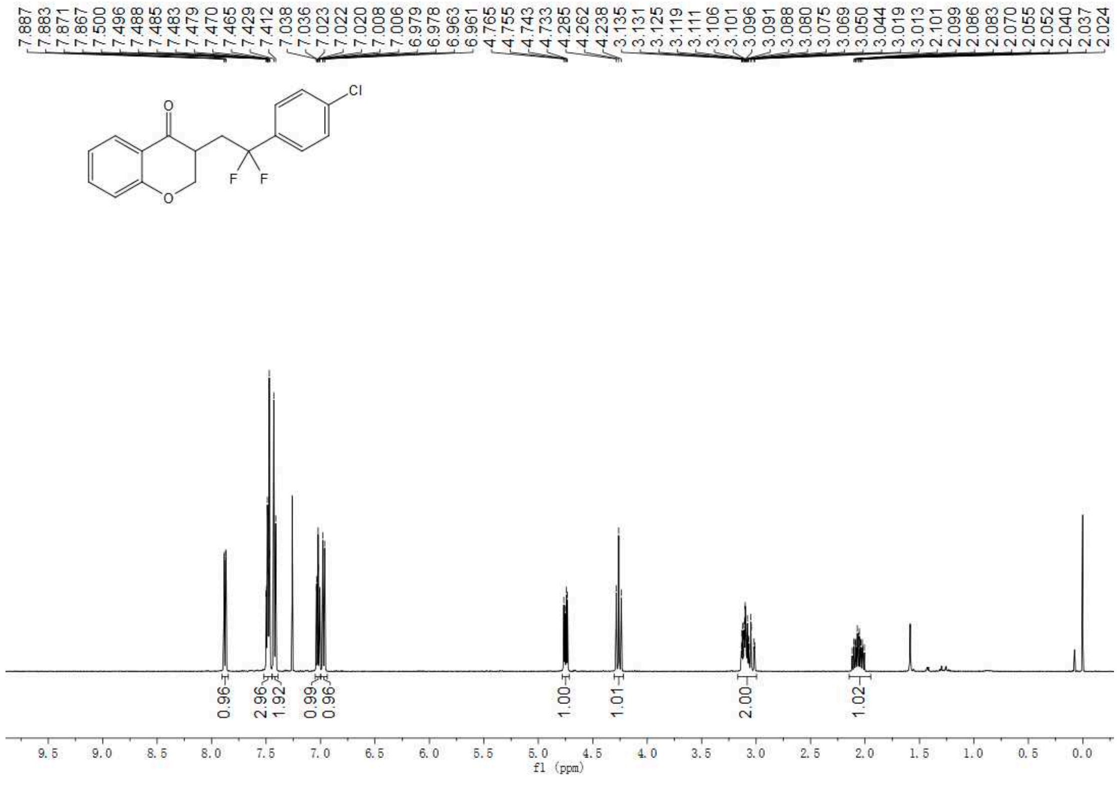

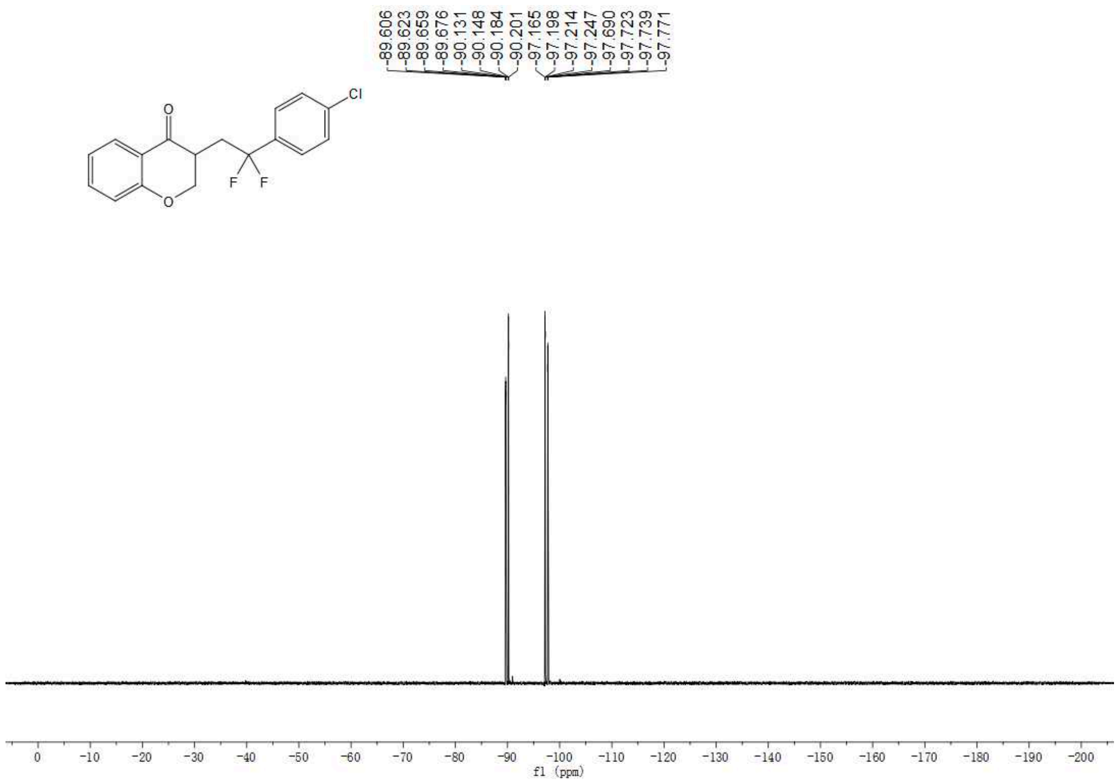

3-(2-(4-bromophenyl)-2,2-difluoroethyl)chroman-4-one (3am)

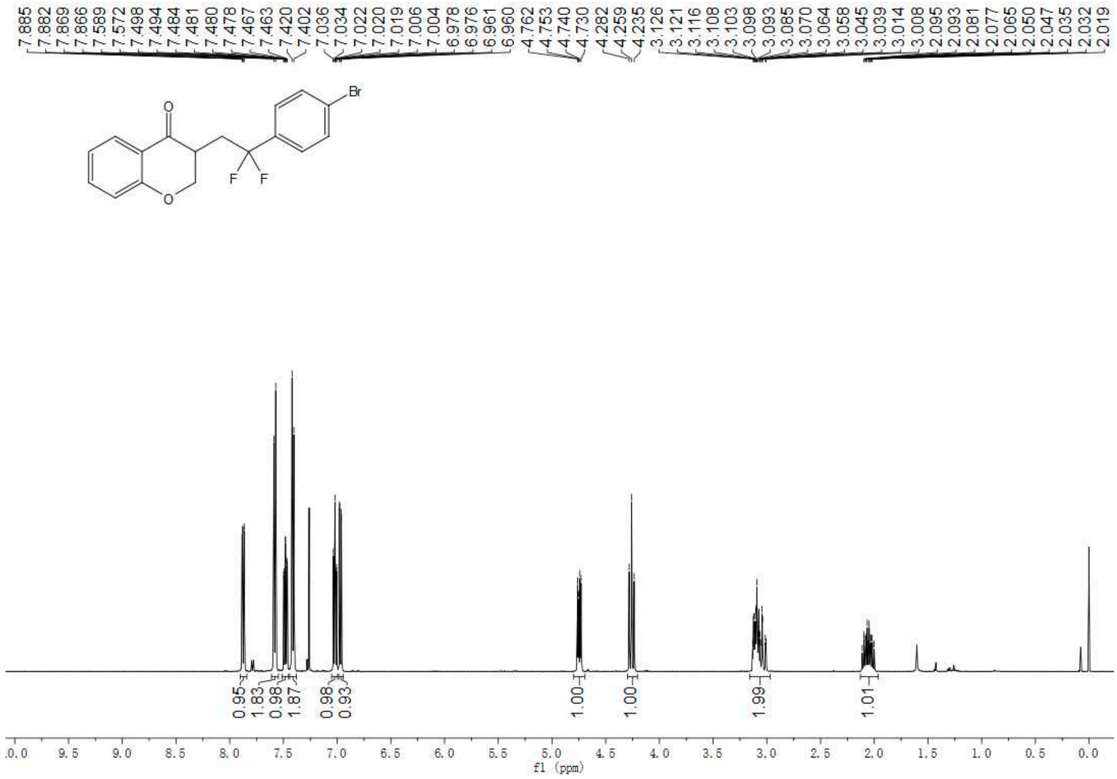

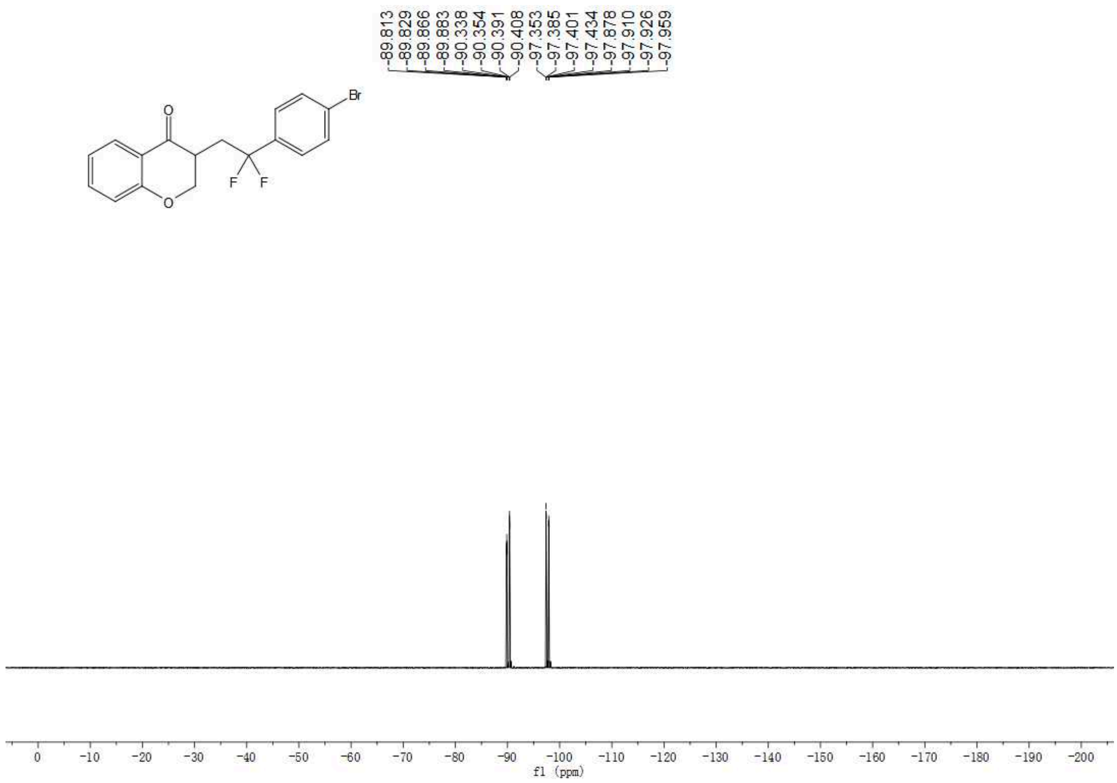

3-(2-(3-bromophenyl)-2,2-difluoroethyl)chroman-4-one(3an)

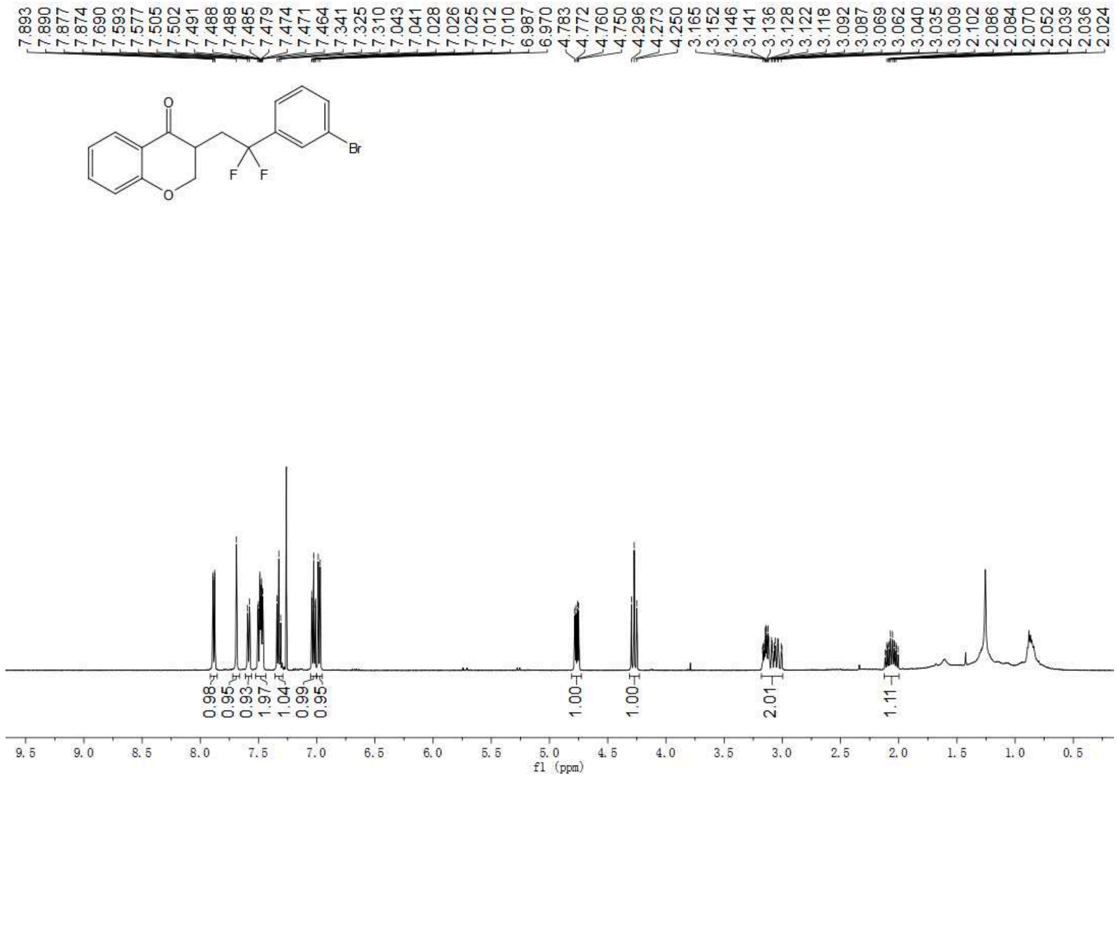

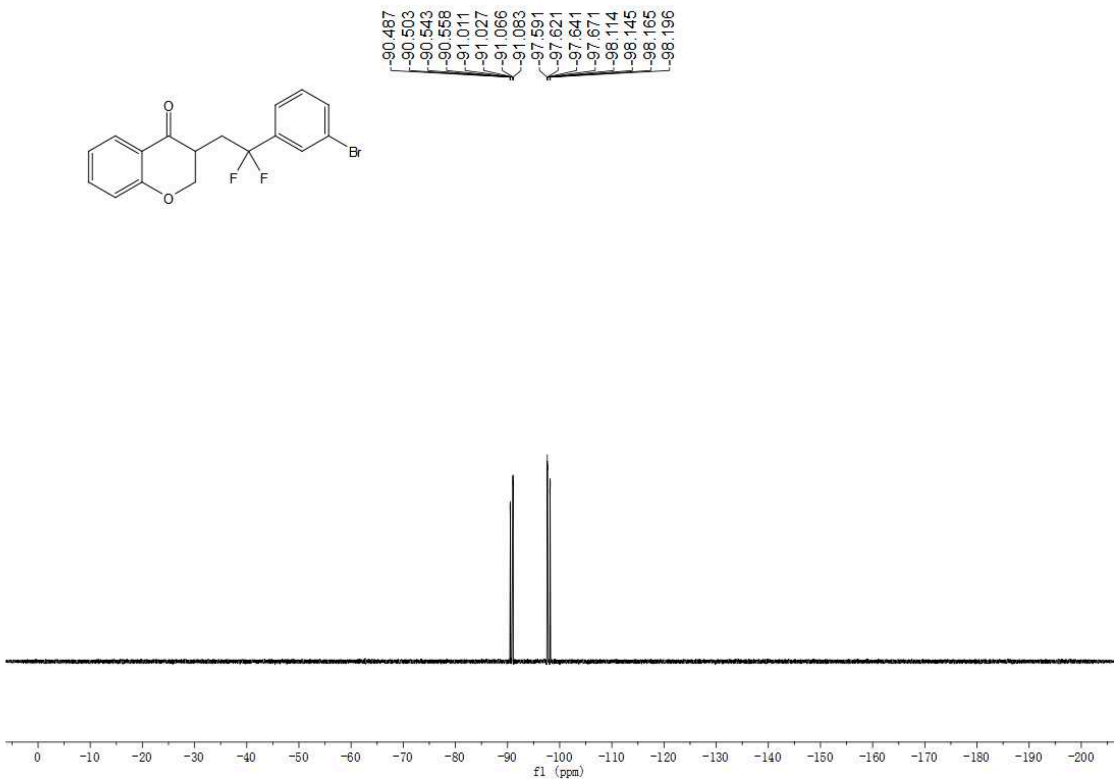

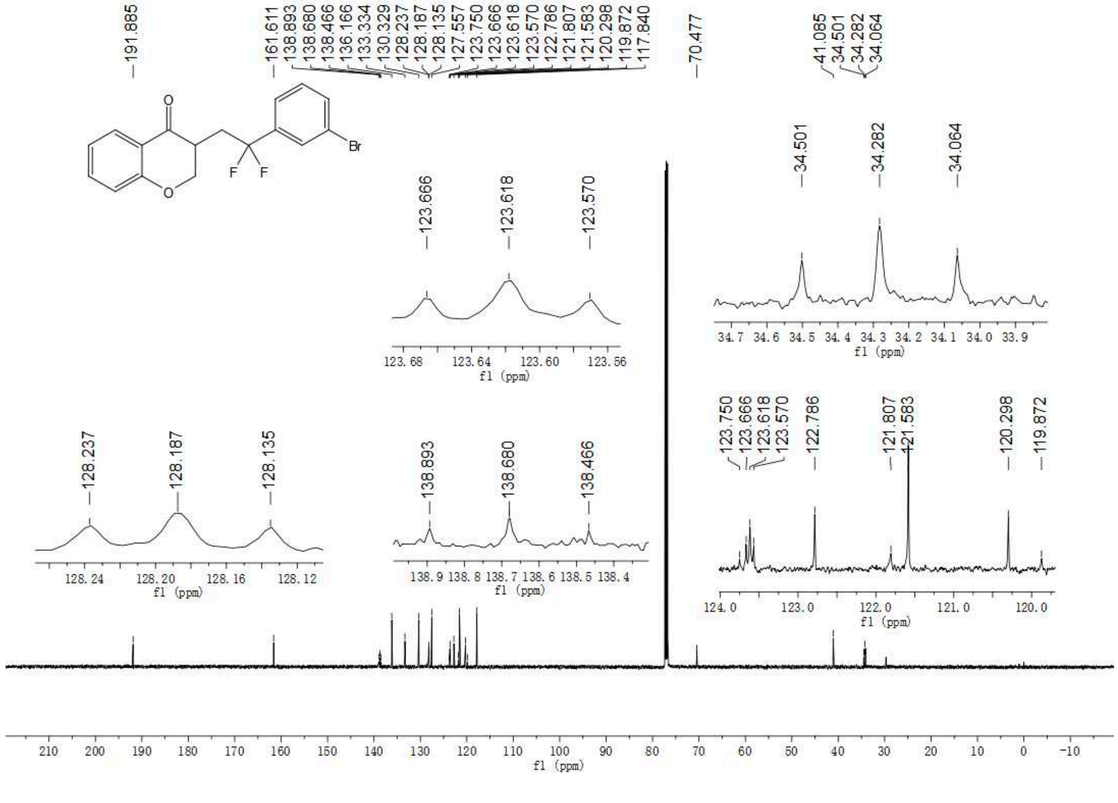

3-(2,2-difluoro-2-(4-iodophenyl)ethyl)chroman-4-one (3ao)

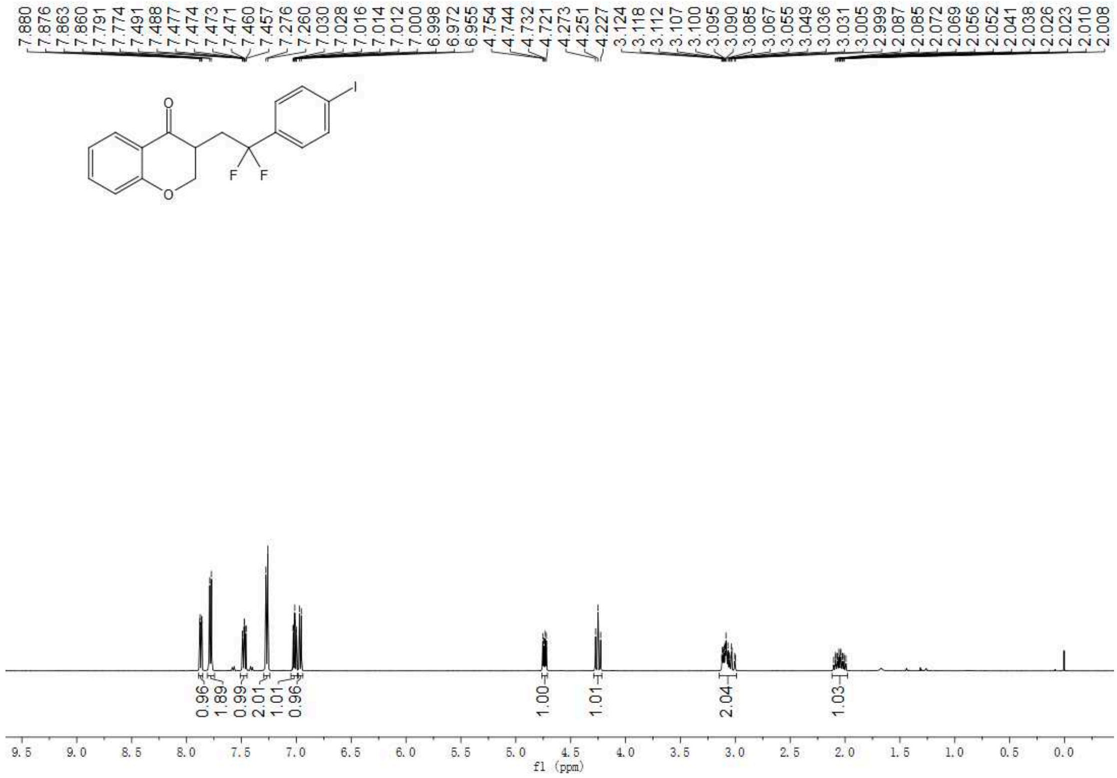

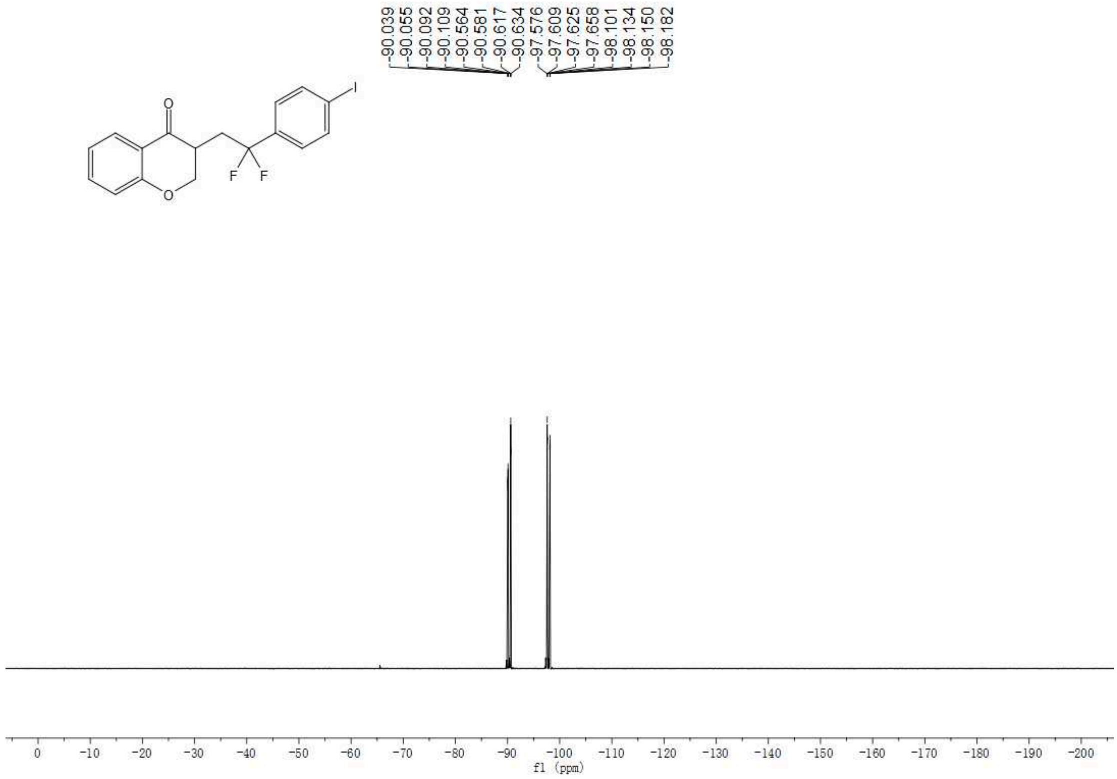

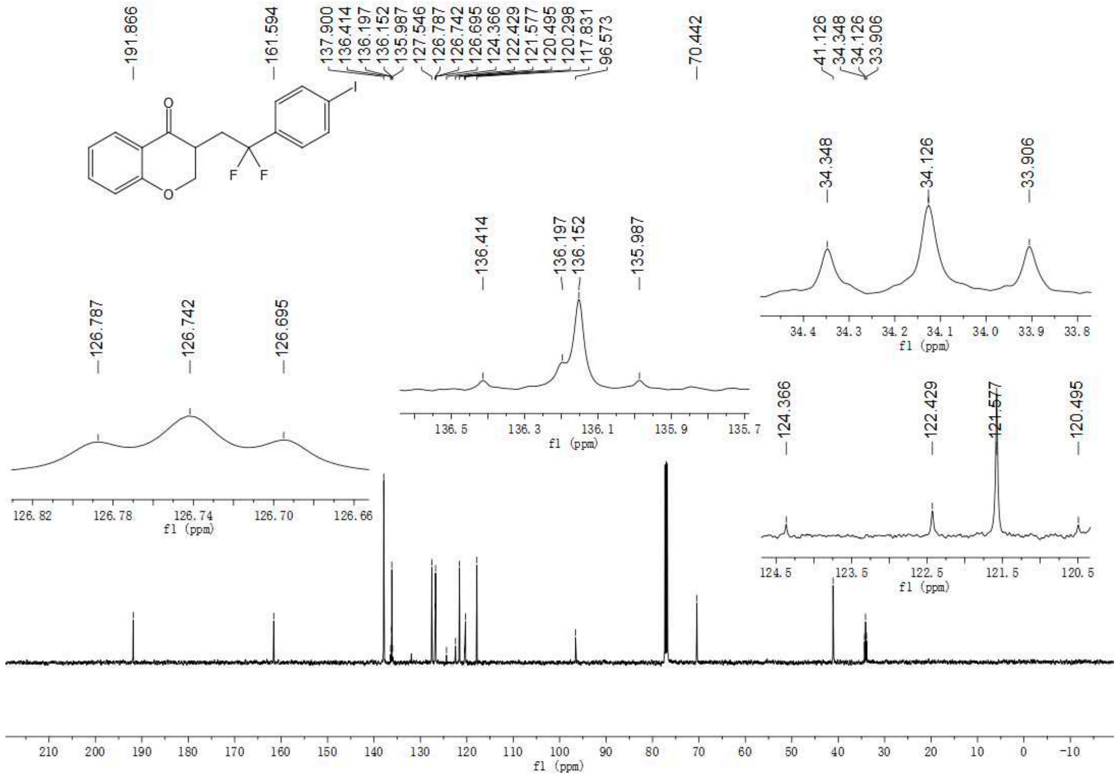

3-(2,2-difluoro-2-((4-methoxyphenyl)thio)ethyl)chroman-4-one (3aq)

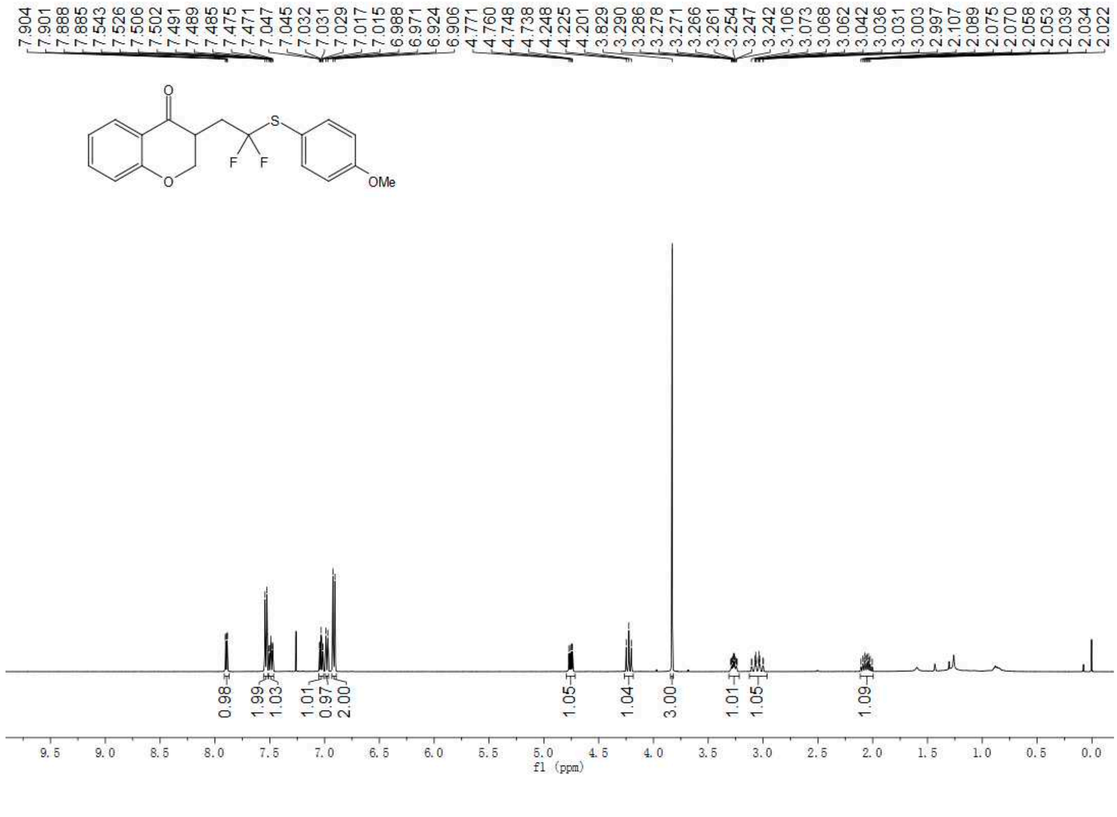

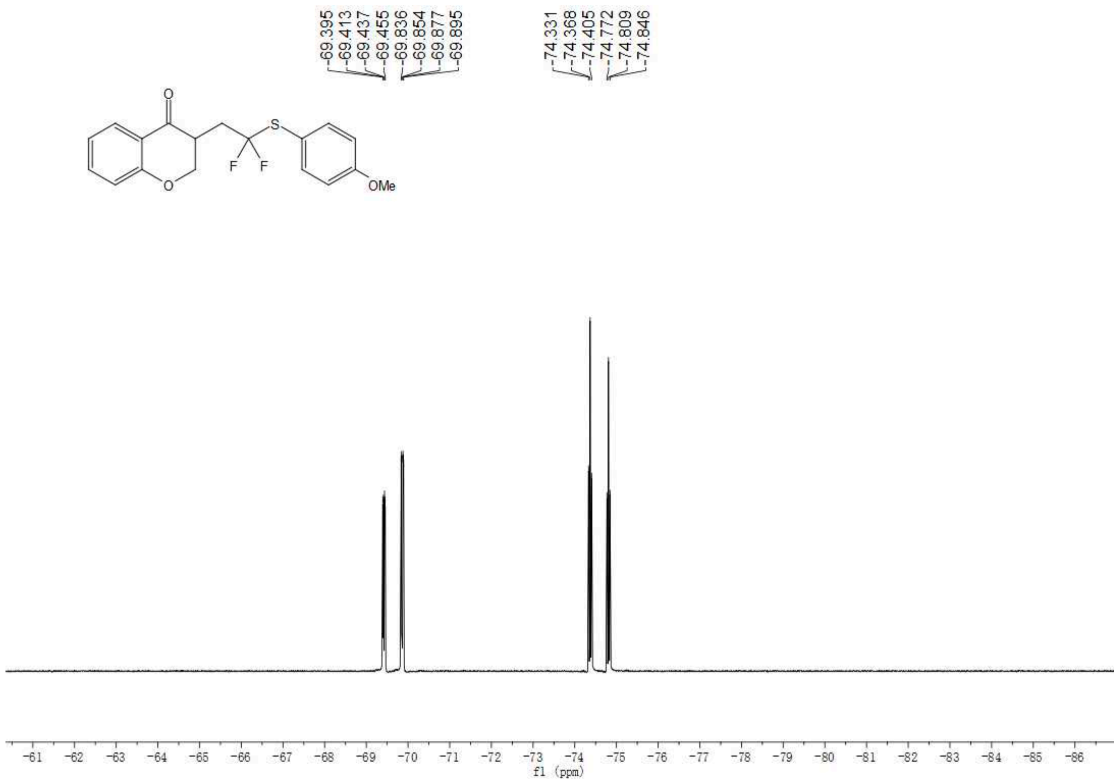
-69.395
-69.413
-69.437
-69.455
-69.836
-69.854
-69.877
-69.895
-74.331
-74.368
-74.405
-74.772
-74.809
-74.846
O
S
F F
O
OMe
f1 (ppm)

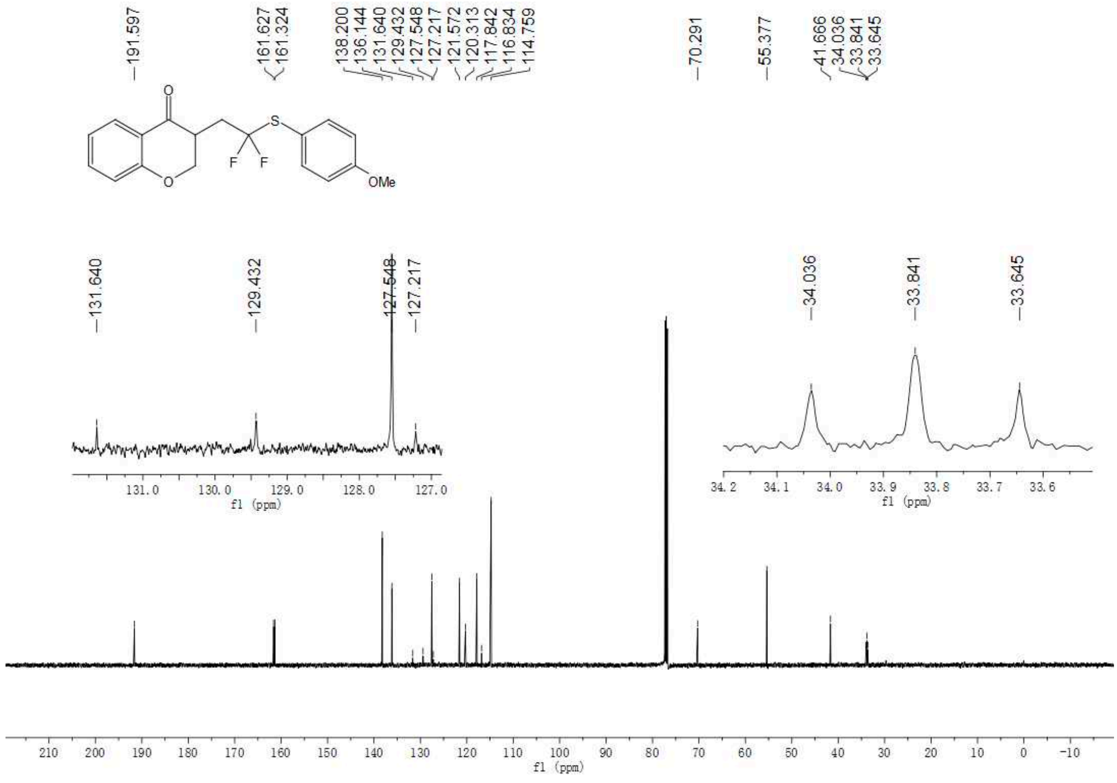
191.597
161.627
161.324
138.200
136.144
131.640
129.432
127.548
127.217
121.572
120.313
117.842
116.834
114.759
70.291
55.377
41.666
34.036
33.841
33.645
O
S
F F
O
OMe
131.640
129.432
127.548
127.217
f1 (ppm)
34.036
33.841
33.645
f1 (ppm)
f1 (ppm)

2-((2,2-difluoro-6-methoxythiochroman-4-yl)methoxy)benzaldehyde (3aq’)

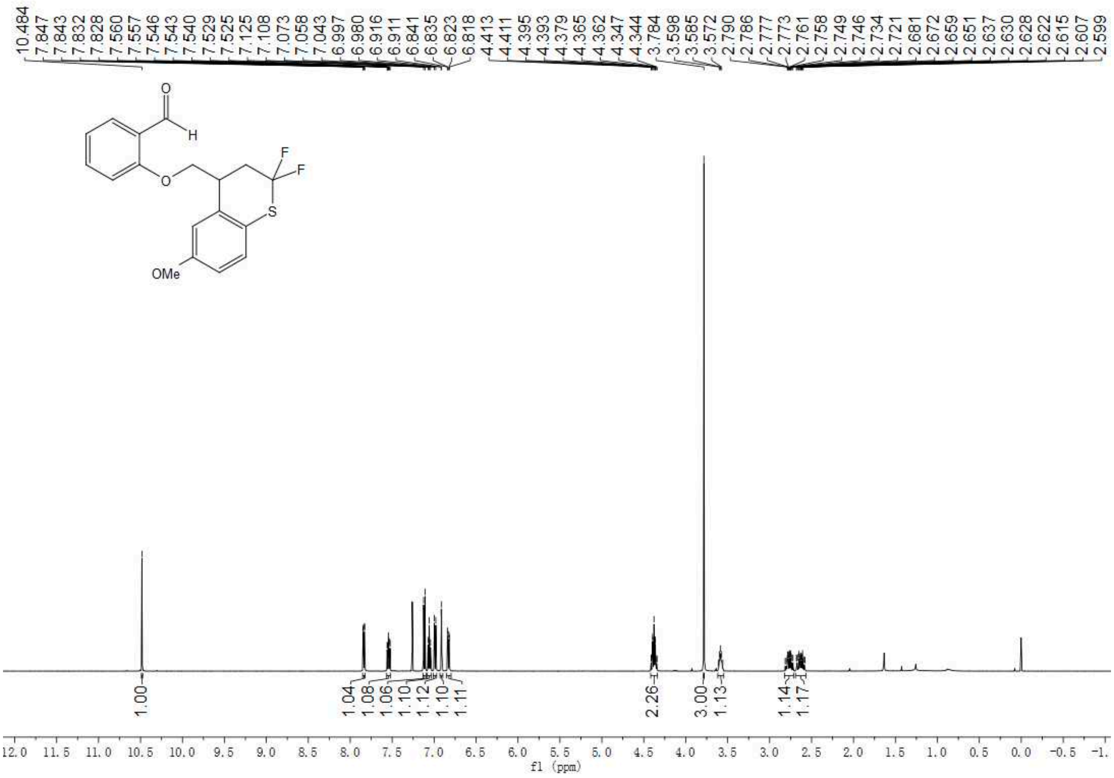

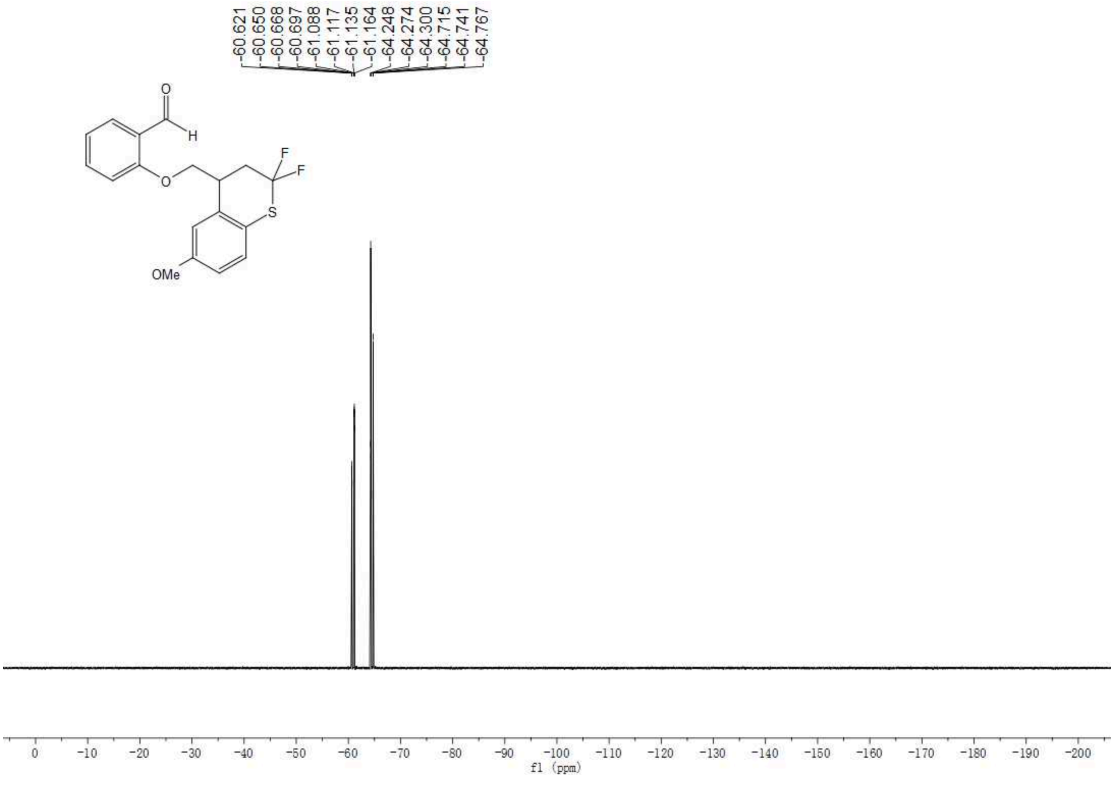

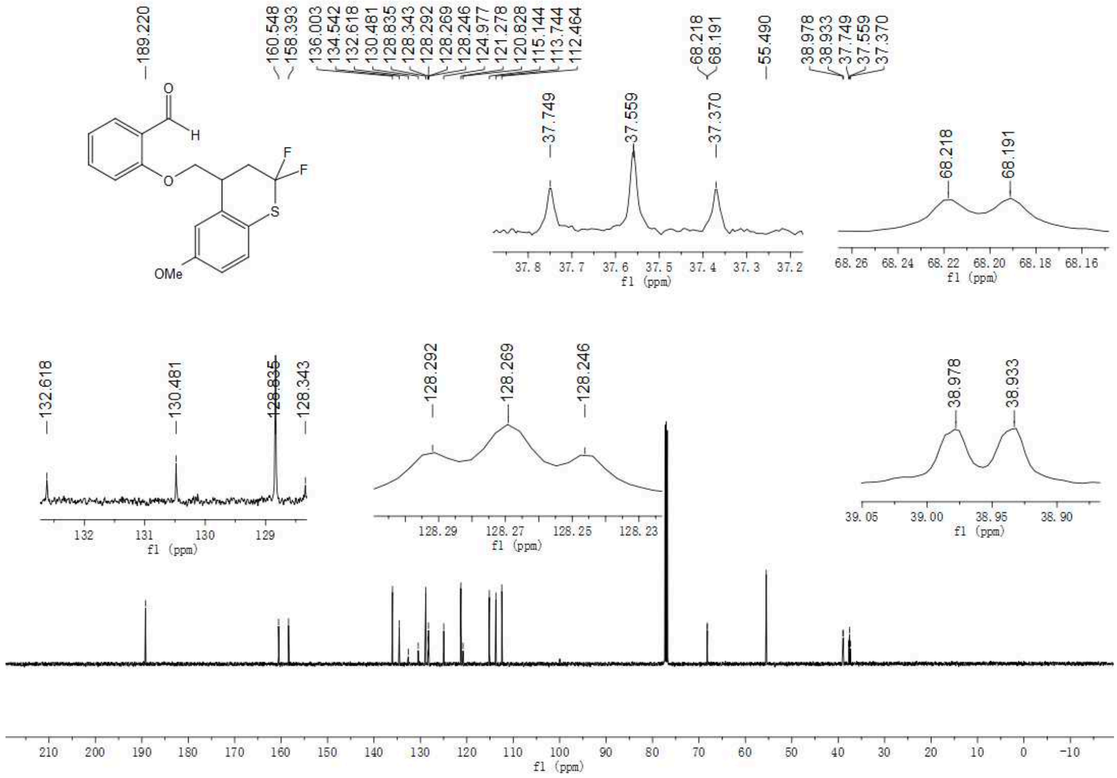

3-(2,2-difluorobutyl)chroman-4-one (3ar)

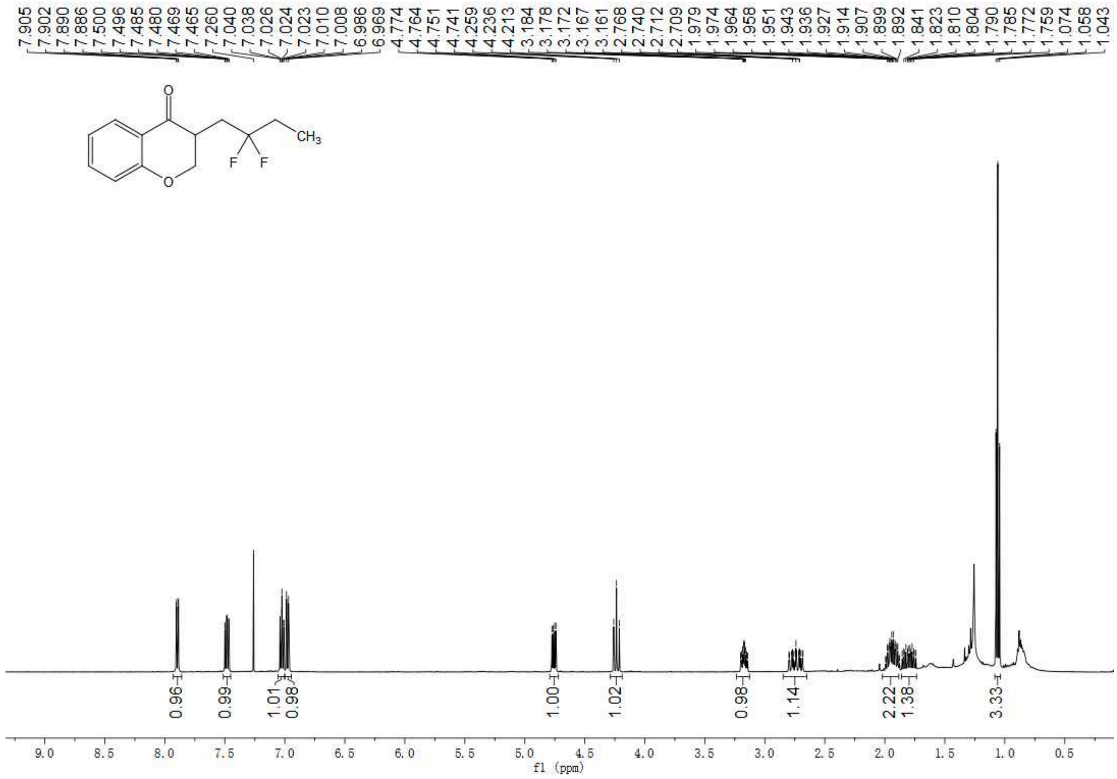

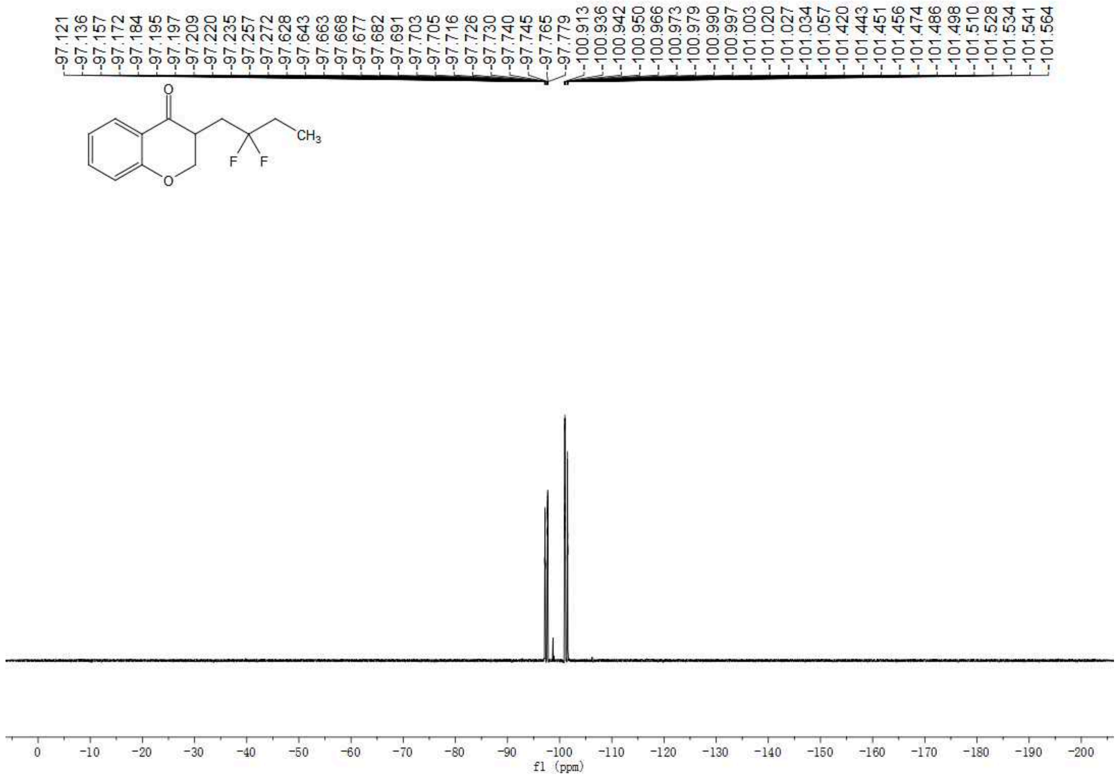

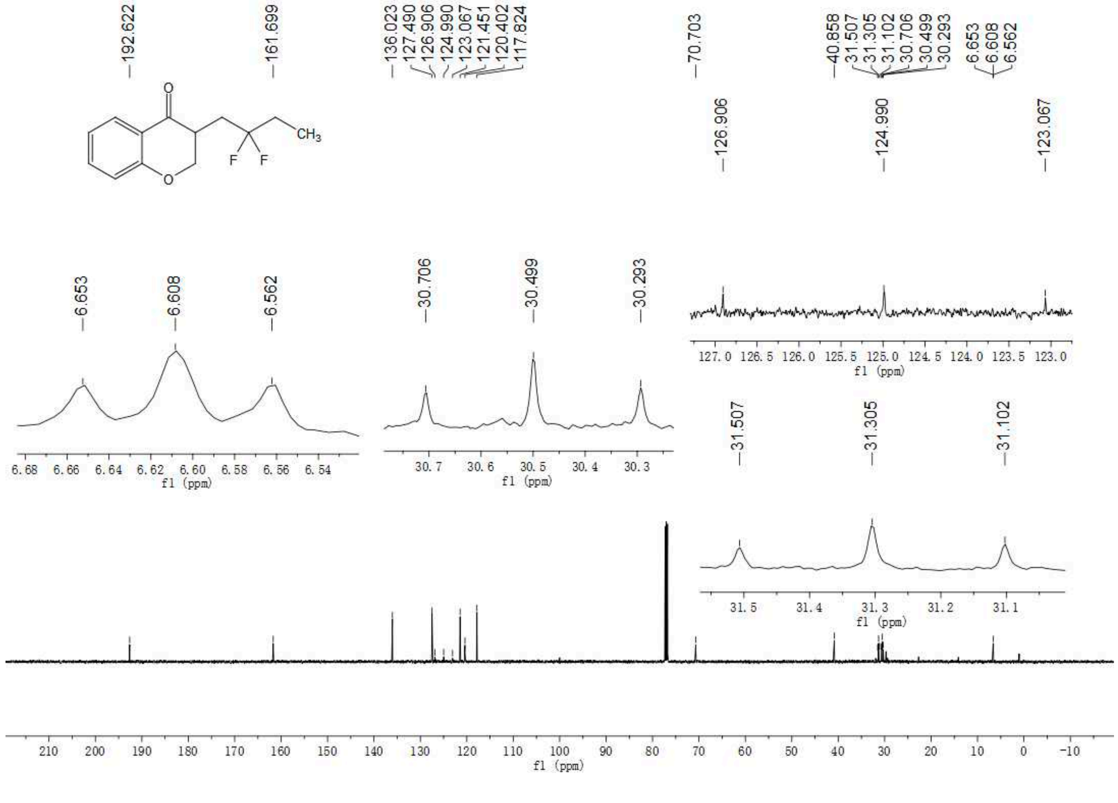

3-(2,2-difluoro-2-(4-methoxyphenyl)ethyl)-7-methoxychroman-4-one (3bb)

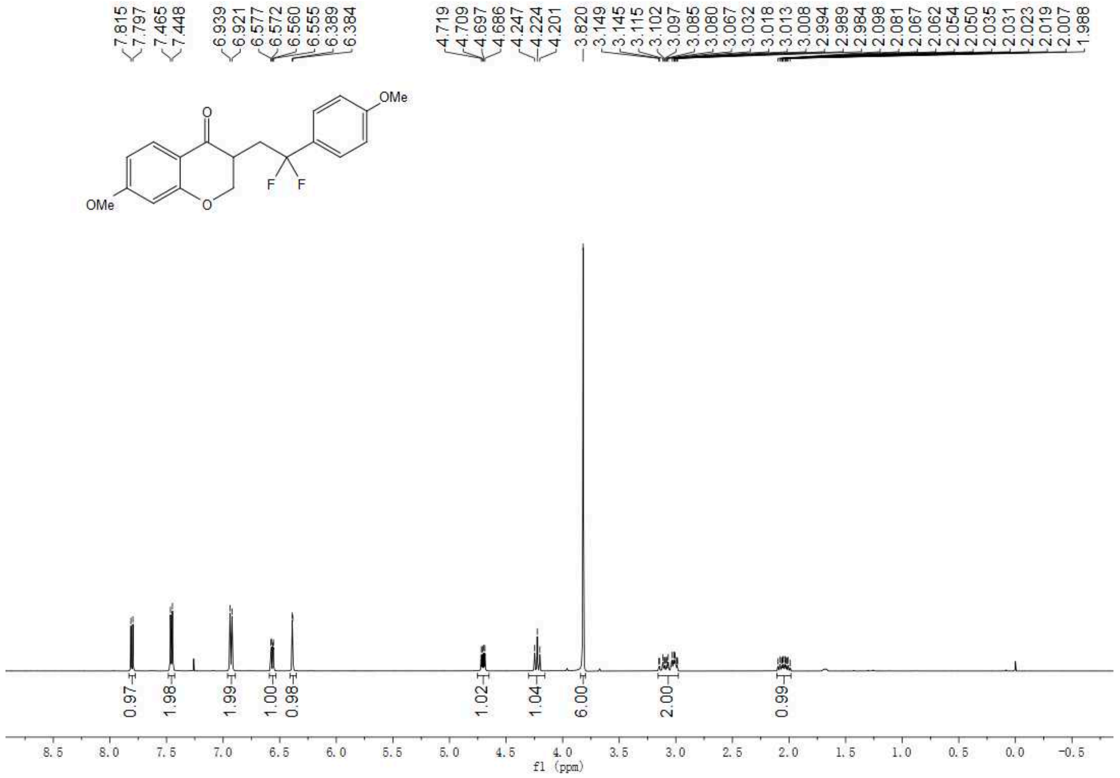

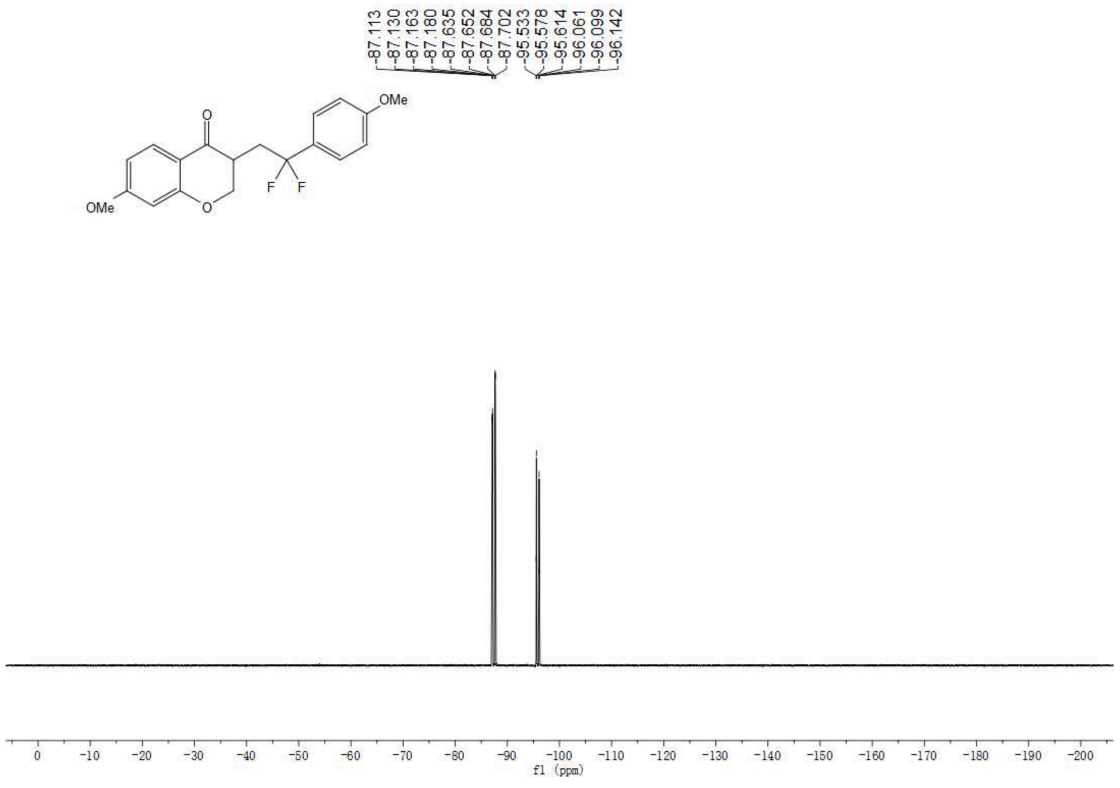

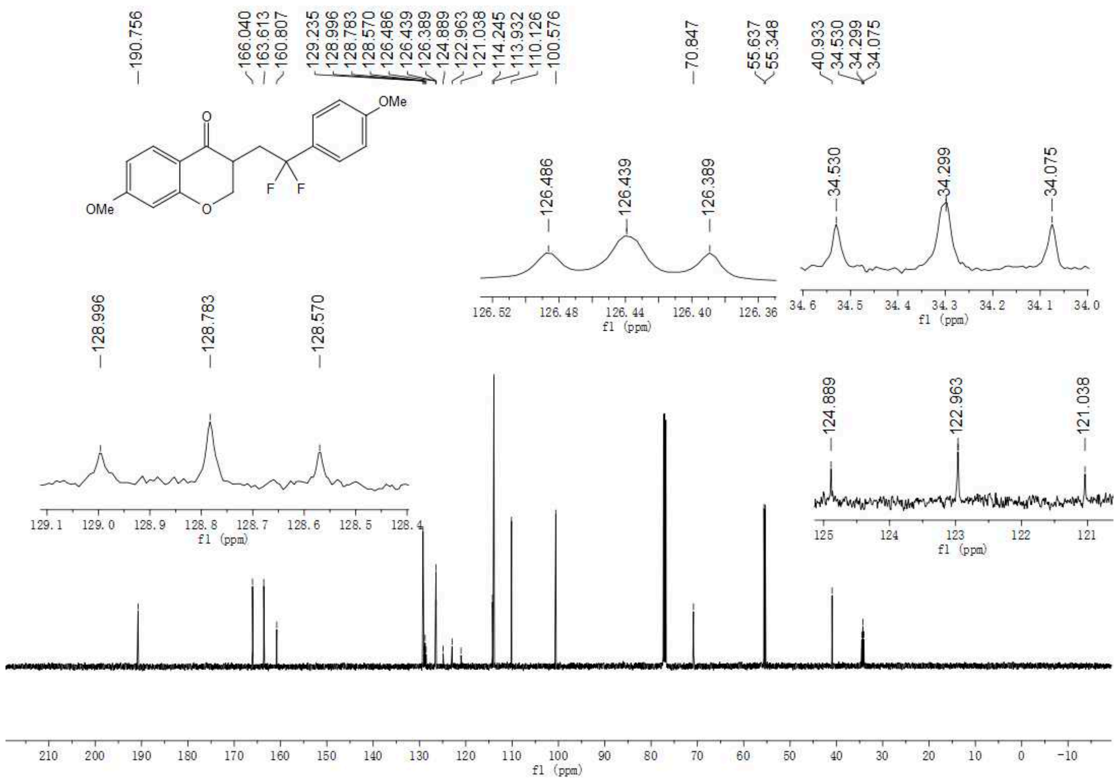

3-(2,2-difluoro-2-(4-methoxyphenyl)ethyl)-6-methylchroman-4-one (1cb)

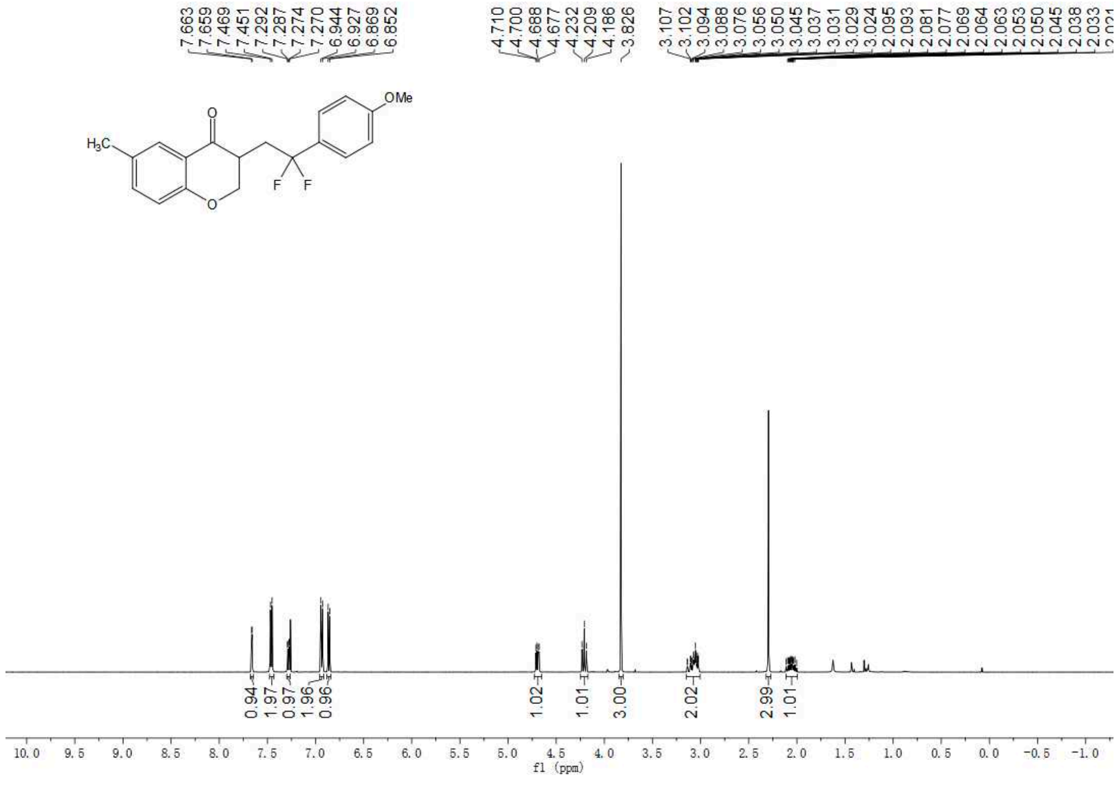

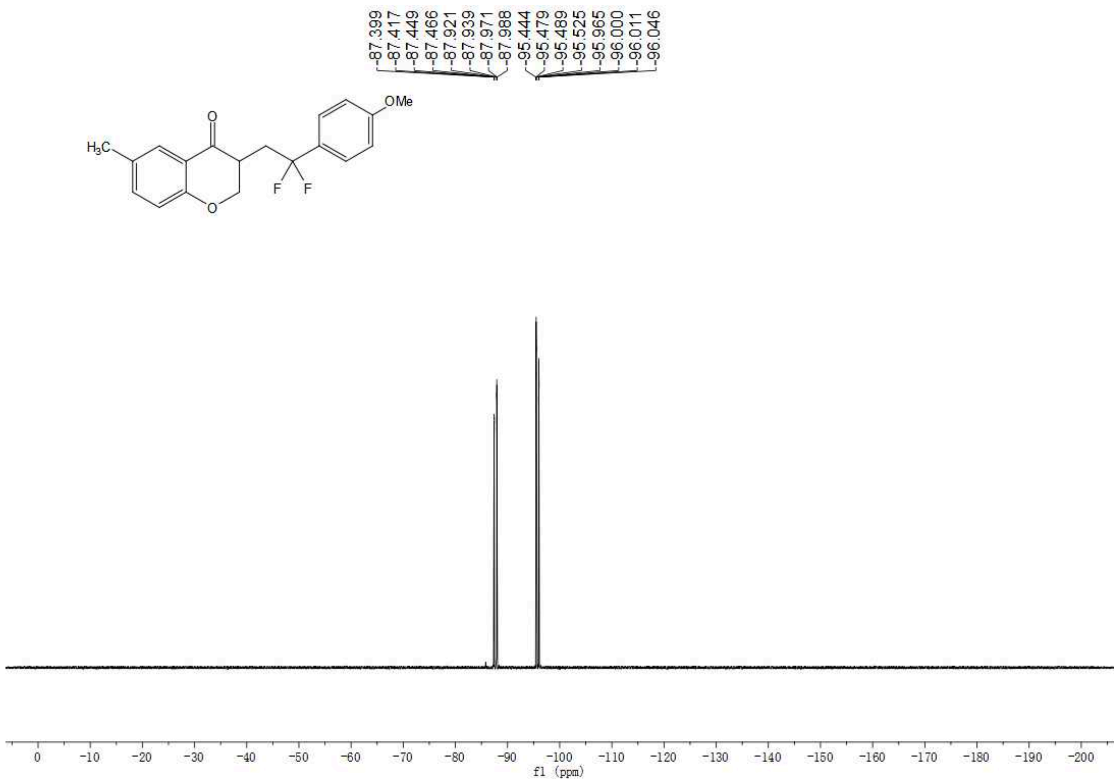
-87.399
-87.417
-87.449
-87.466
-87.921
-87.939
-87.971
-87.988
-95.444
-95.479
-95.489
-95.525
-95.965
-96.000
-96.011
-96.046
OMe
O
H3C
O
F F
f1 (ppm)

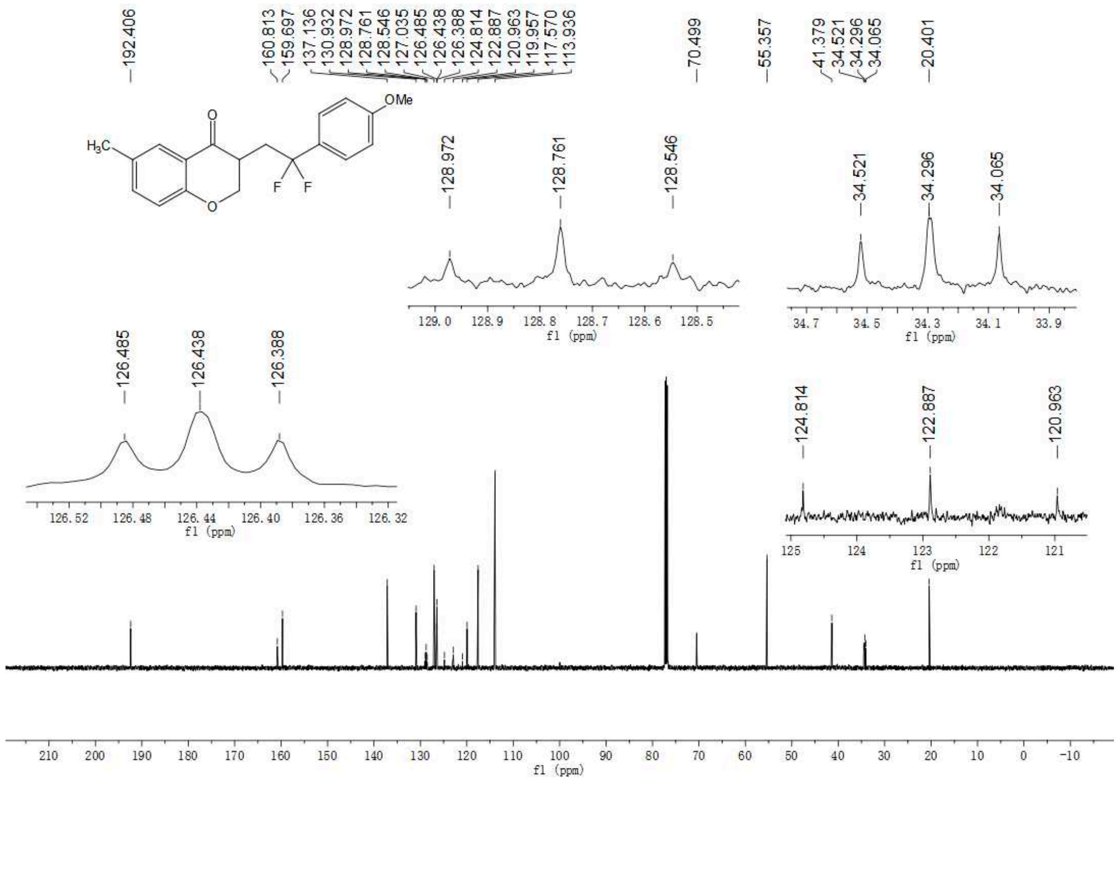
192.406
160.813
159.697
137.136
130.932
128.972
128.761
128.546
127.035
126.485
126.438
126.388
124.814
122.887
120.963
119.957
117.570
113.936
70.499
55.357
41.379
34.521
34.296
34.065
20.401
OMe
O
H3C
O
F F
f1 (ppm)

3-(2,2-difluoro-2-(4-methoxyphenyl)ethyl)-7-methylchroman-4-one (1db)

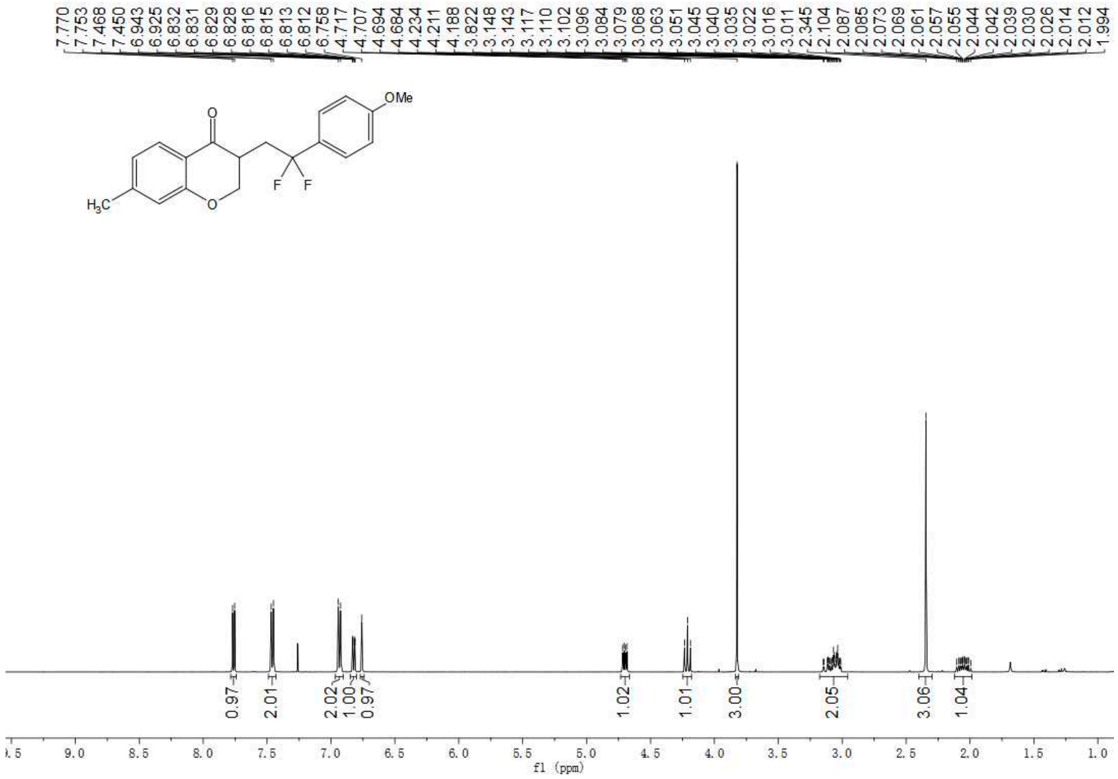

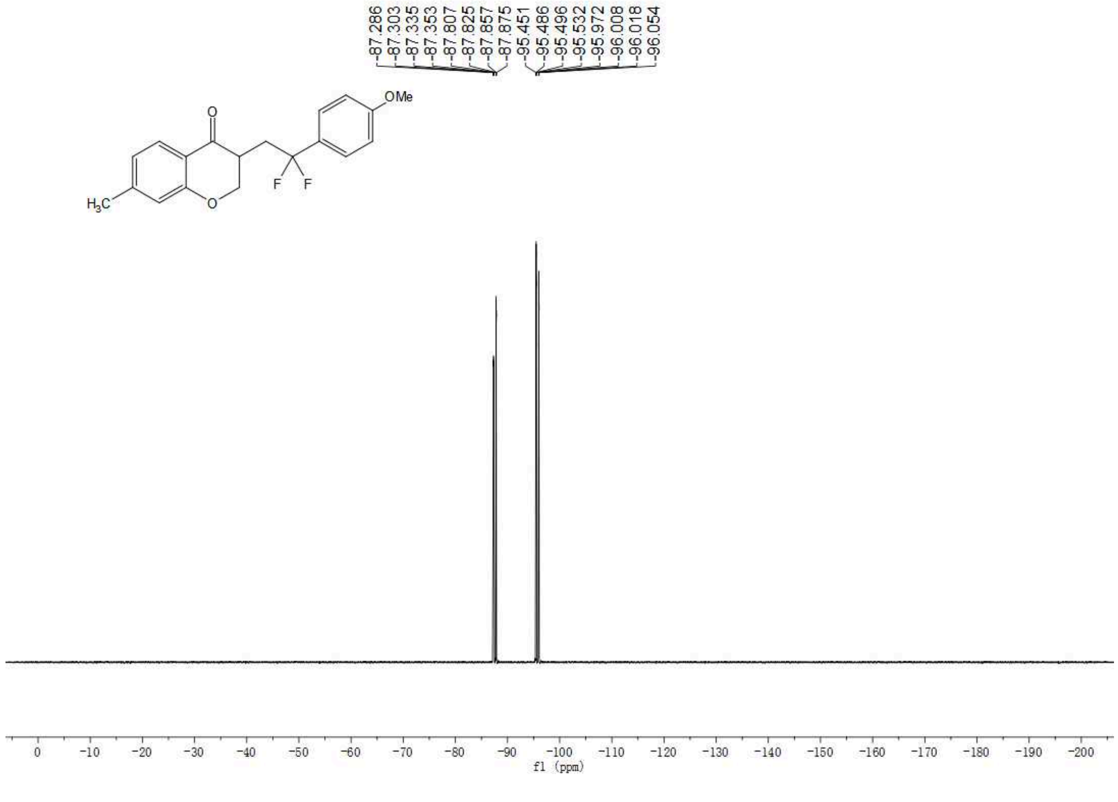

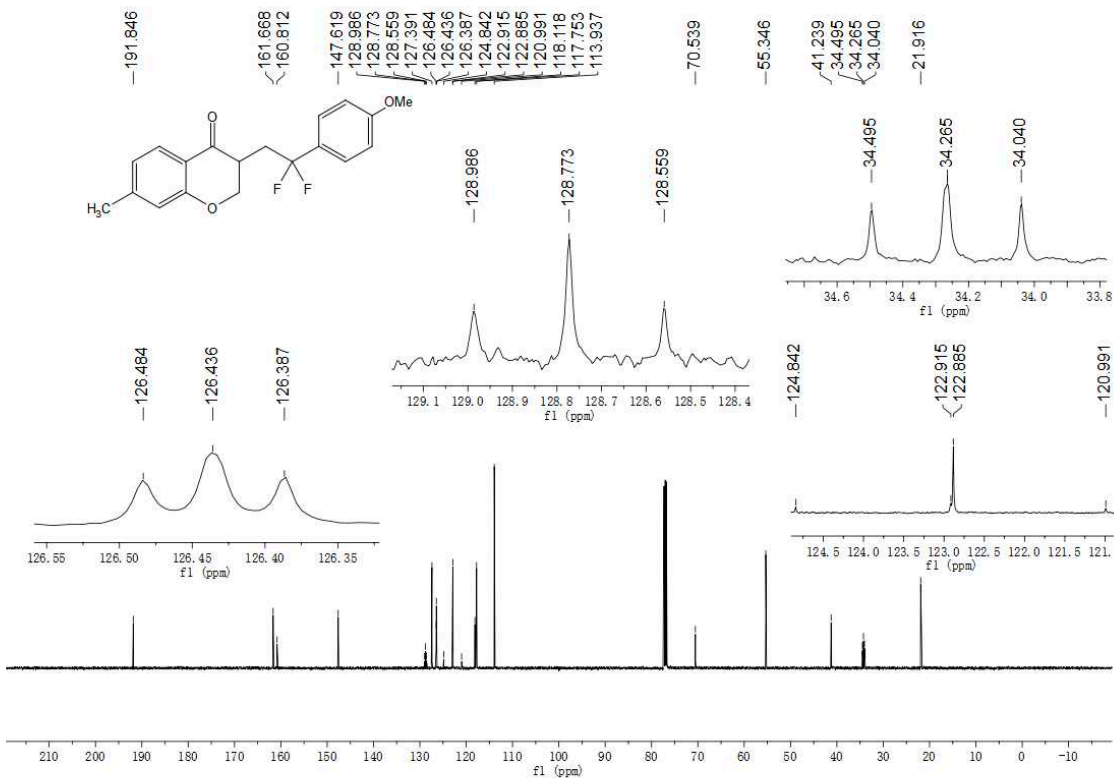

6,8-di-tert-butyl-3-(2,2-difluoro-2-(4-methoxyphenyl)ethyl)chroman-4-one (1eb)

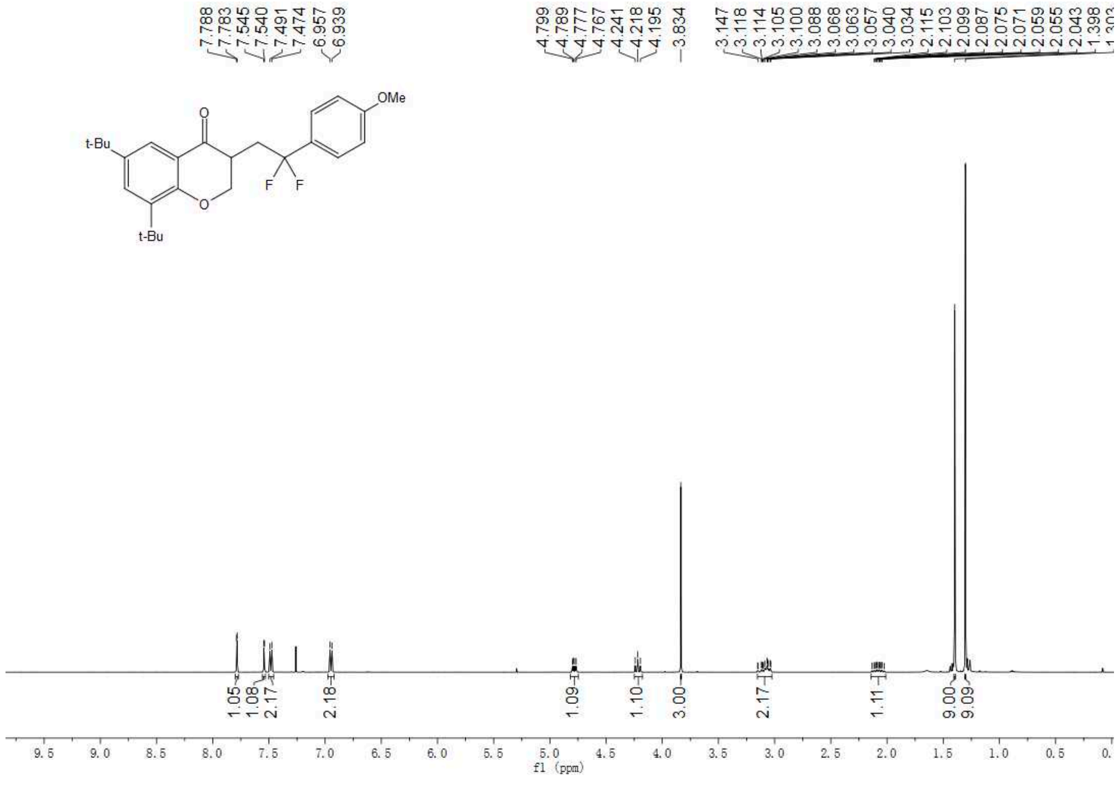

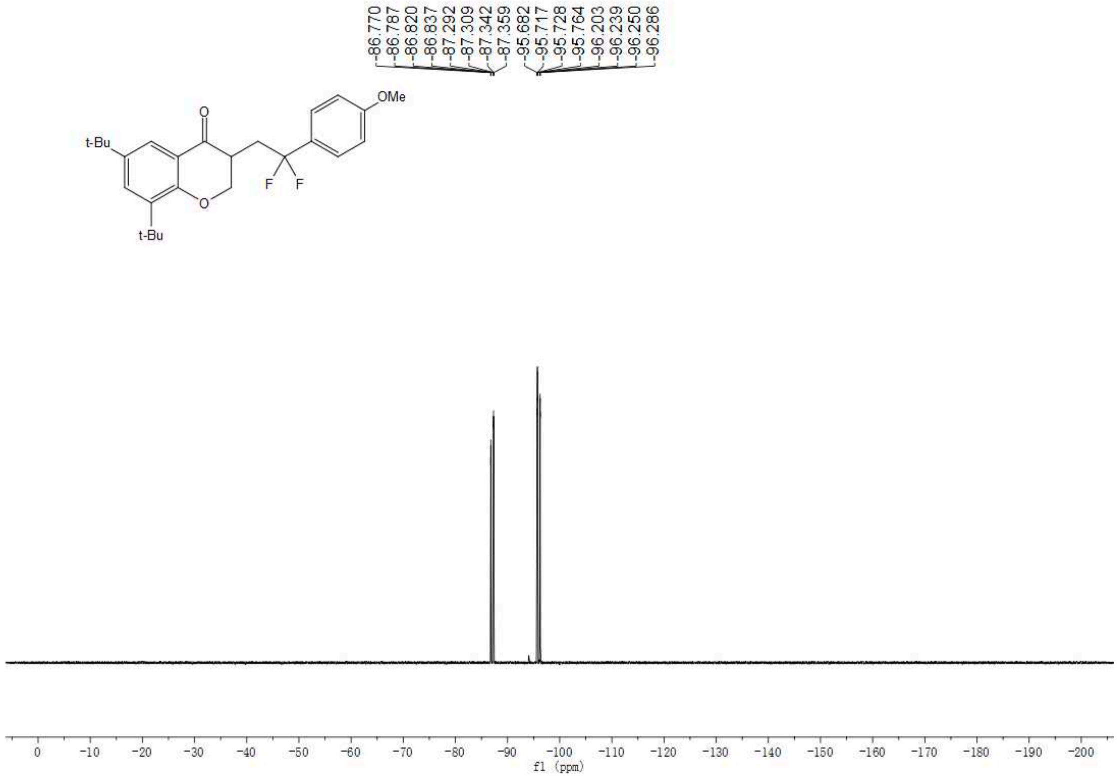
-86.770
-86.787
-86.820
-86.837
-87.292
-87.309
-87.342
-87.359
-95.682
-95.717
-95.728
-95.764
-96.203
-96.239
-96.250
-96.286
OMe
t-Bu
t-Bu
O
F F
f1 (ppm)

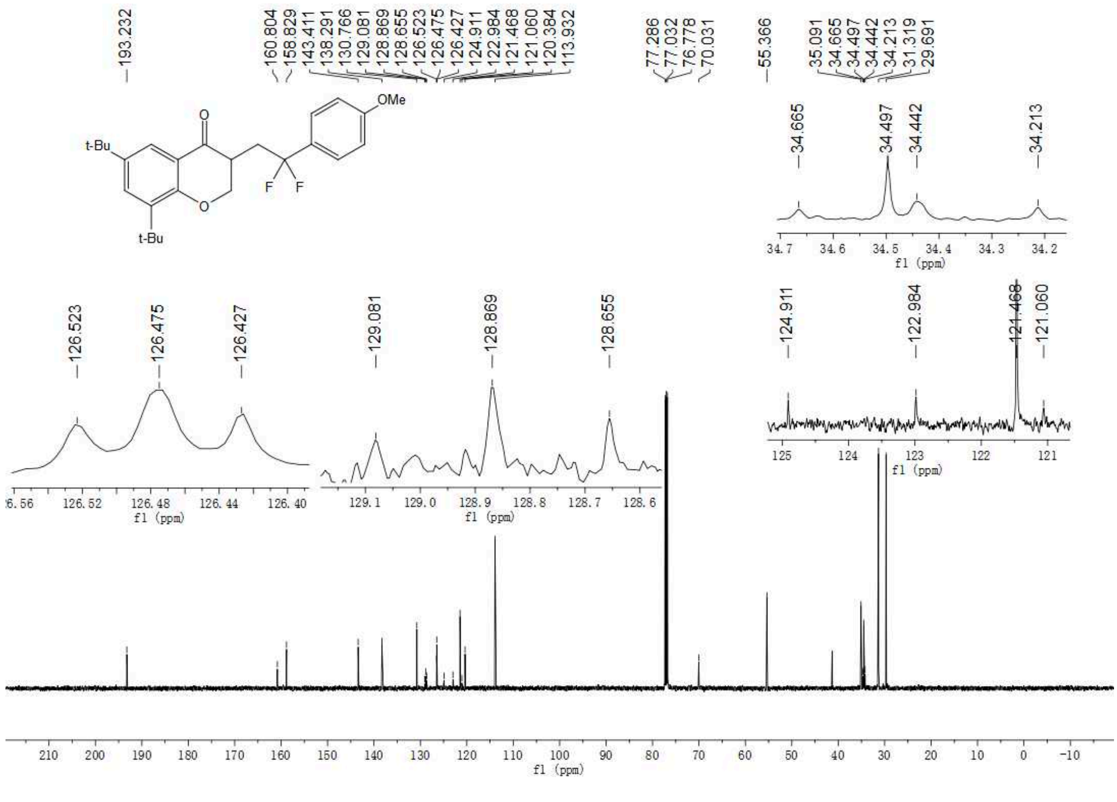
193.232
160.804
158.829
143.411
138.291
130.766
129.081
128.869
128.655
126.523
126.475
126.427
124.911
122.984
121.468
121.060
120.384
113.932
77.286
77.032
76.778
70.031
55.366
35.091
34.665
34.497
34.442
34.213
31.319
29.691
OMe
t-Bu
t-Bu
O
F F
f1 (ppm)

3-(2,2-difluoro-2-(4-methoxyphenyl)ethyl)-6-fluorochroman-4-one (1fb)

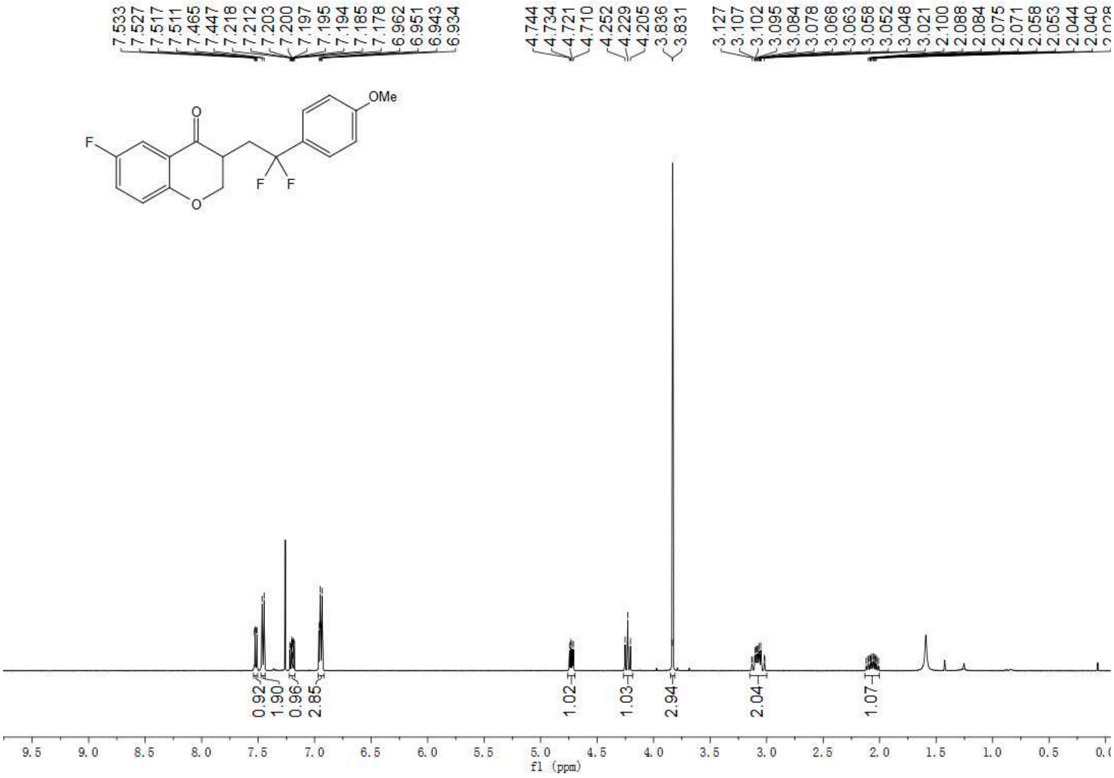

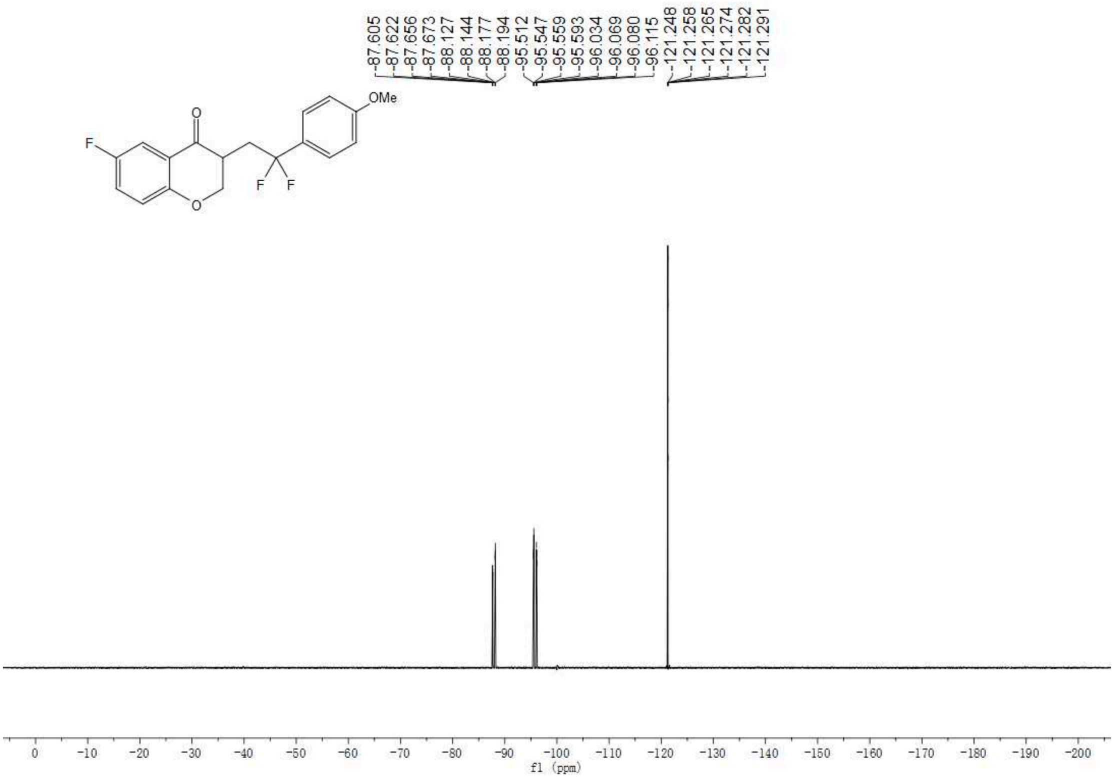

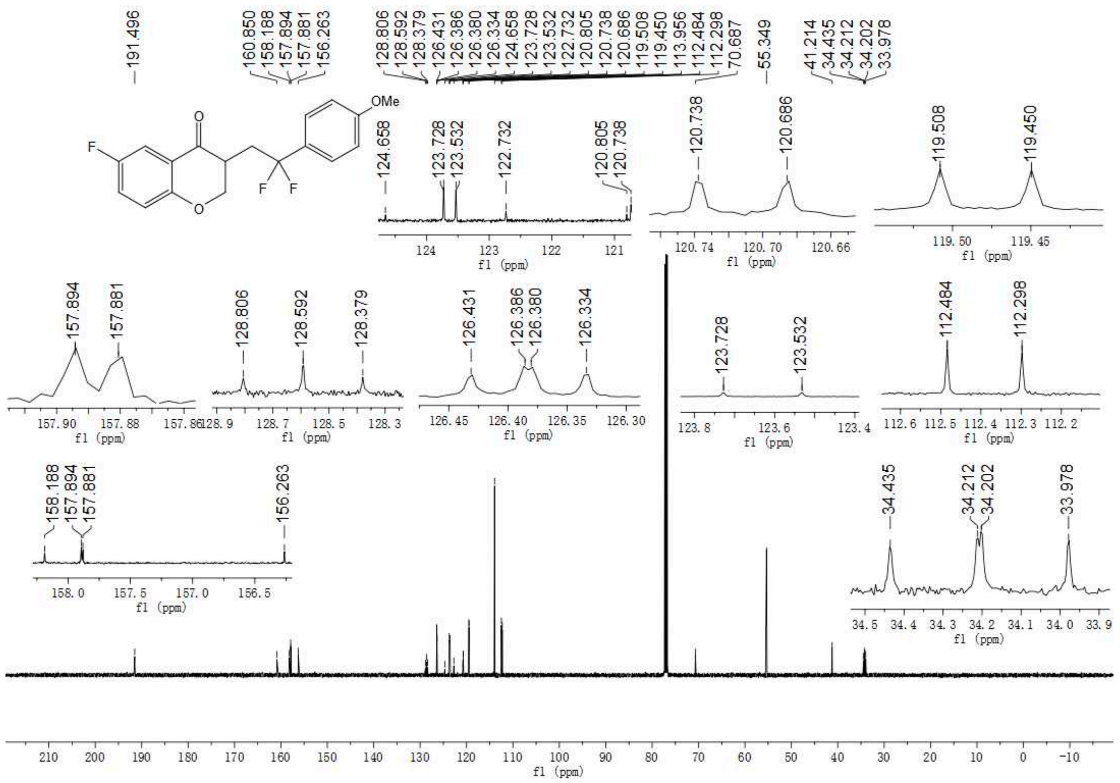

6-Chloro-3-[2,2-difluoro-2-(4-methoxy-phenyl)-ethyl]-chroman-4-one (3gb)

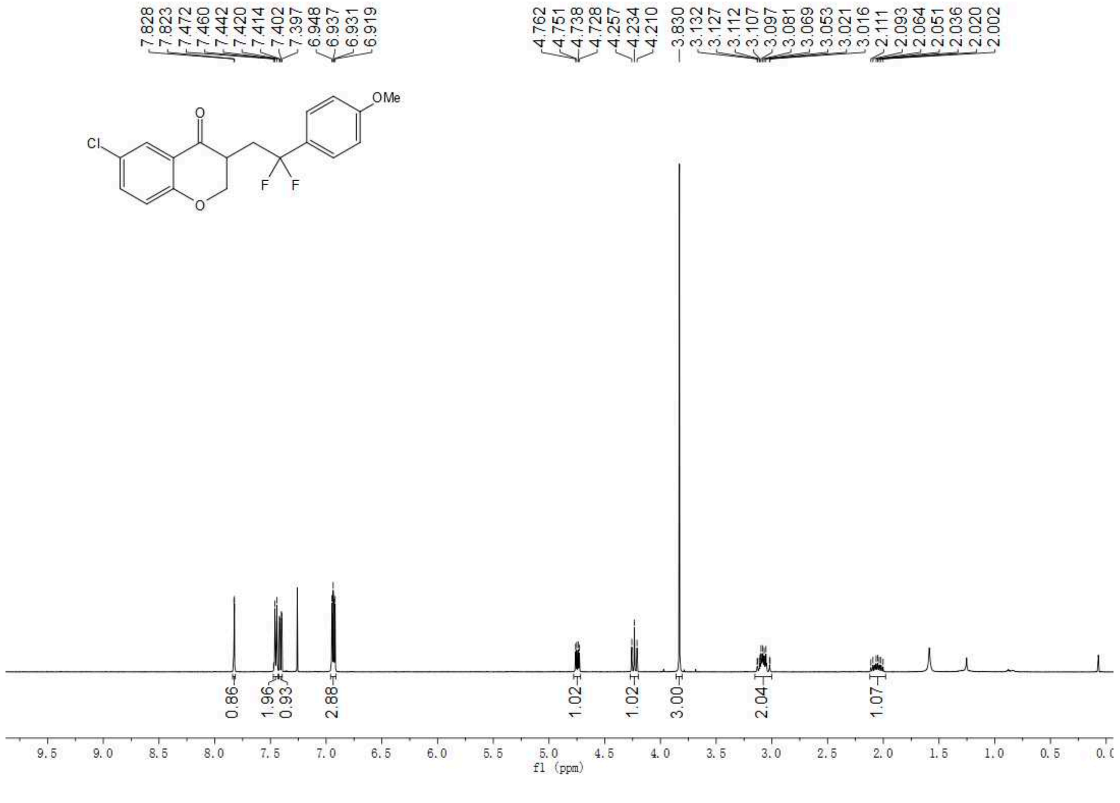

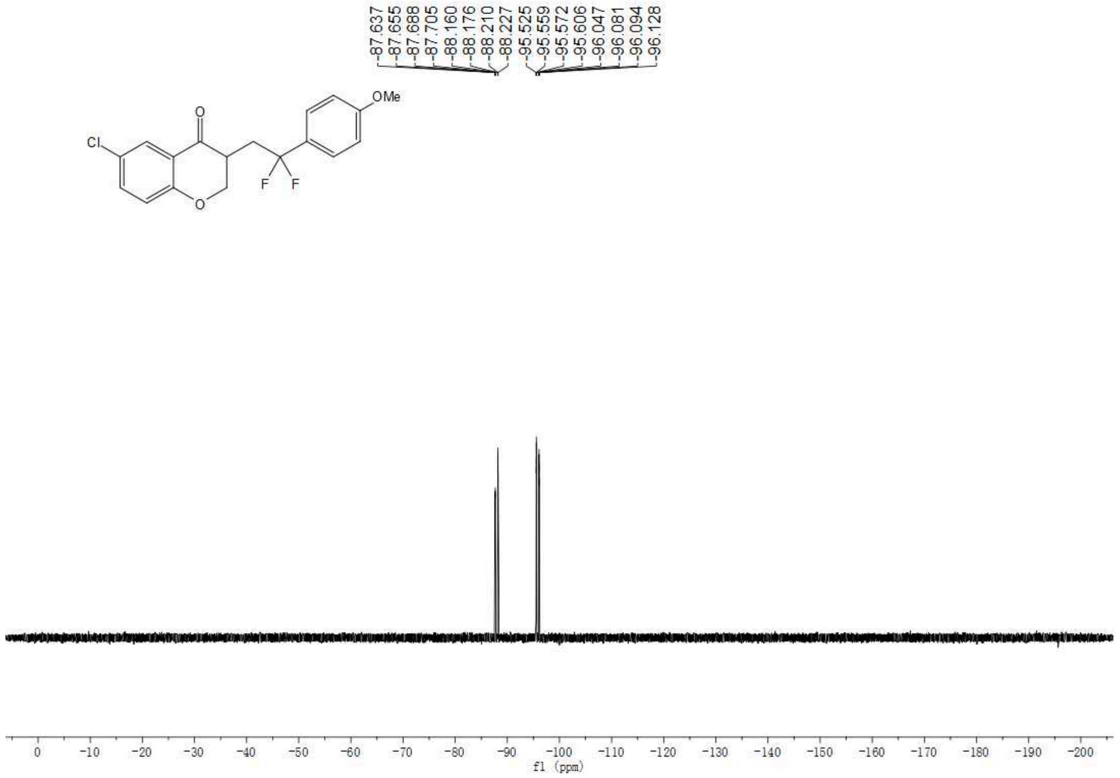
-87.637
-87.655
-87.688
-87.705
-88.160
-88.176
-88.210
-88.227
-95.525
-95.559
-95.572
-95.606
-96.047
-96.081
-96.094
-96.128
OMe
Cl
O
O
F F
f1 (ppm)

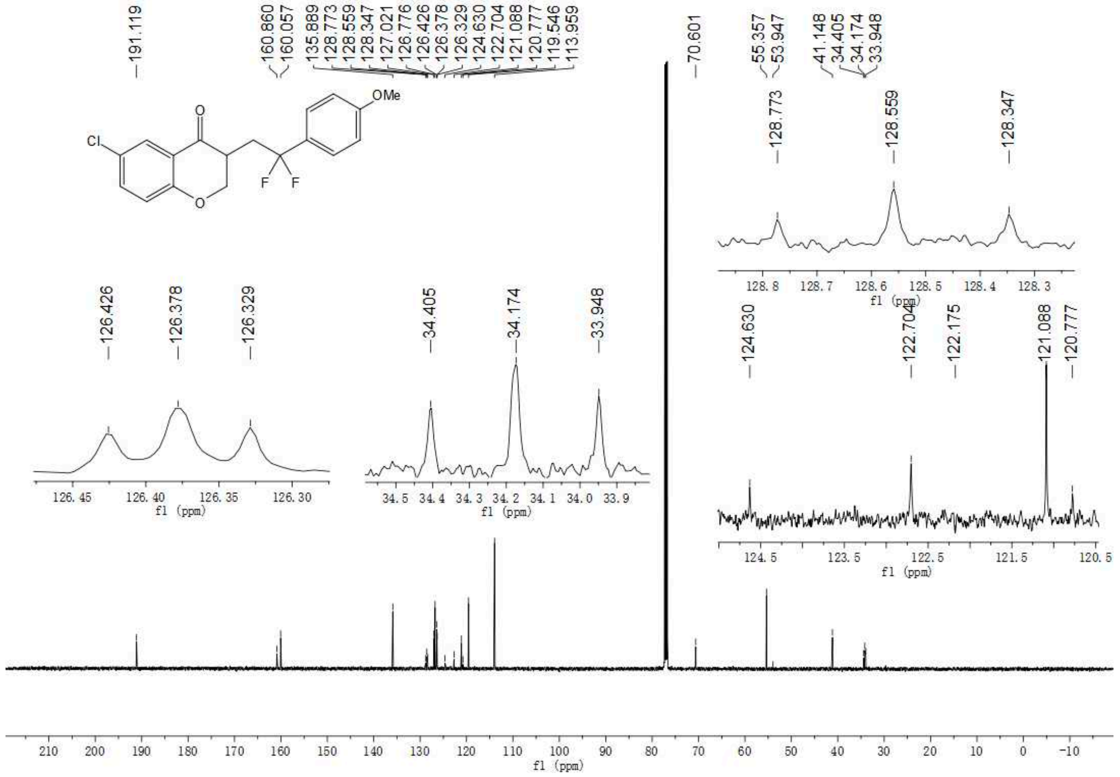
191.119
160.860
160.057
135.889
128.773
128.559
128.347
127.021
126.776
126.426
126.378
126.329
124.630
122.704
121.088
120.777
119.546
113.959
70.601
55.357
53.947
41.148
34.405
34.174
33.948
122.175
OMe
Cl
O
O
F F
f1 (ppm)

6-bromo-3-(2,2-difluoro-2-(4-methoxyphenyl)ethyl)chroman-4-one(3hb)

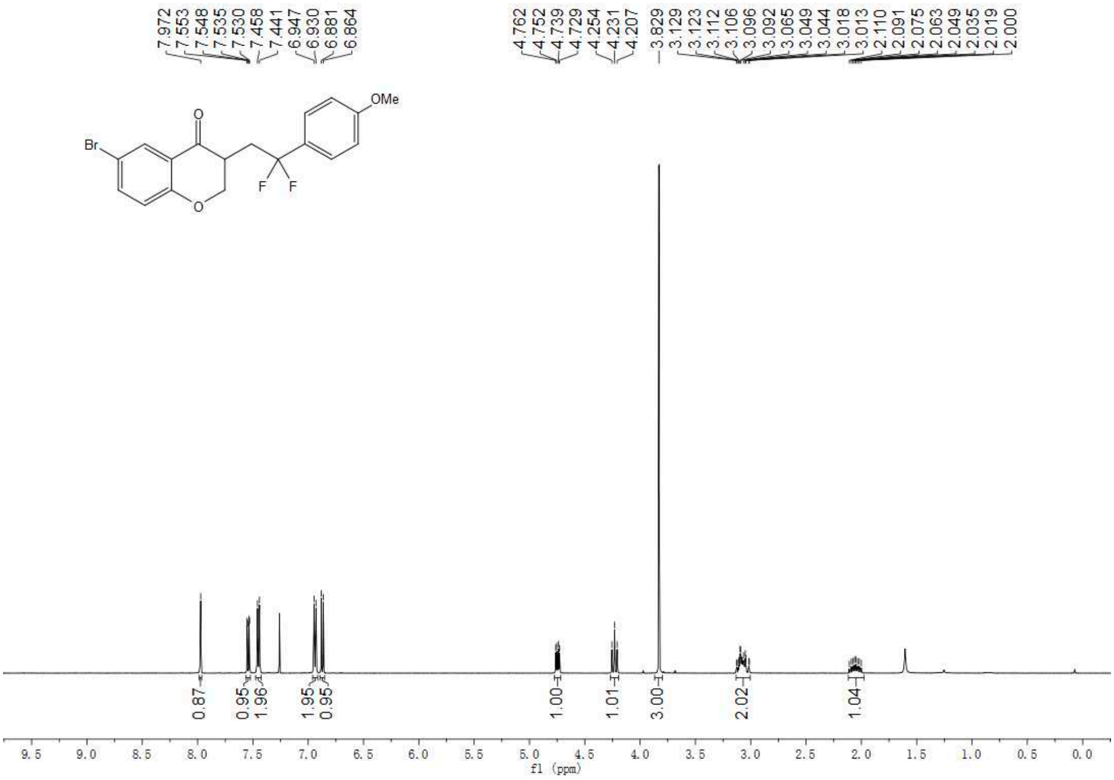

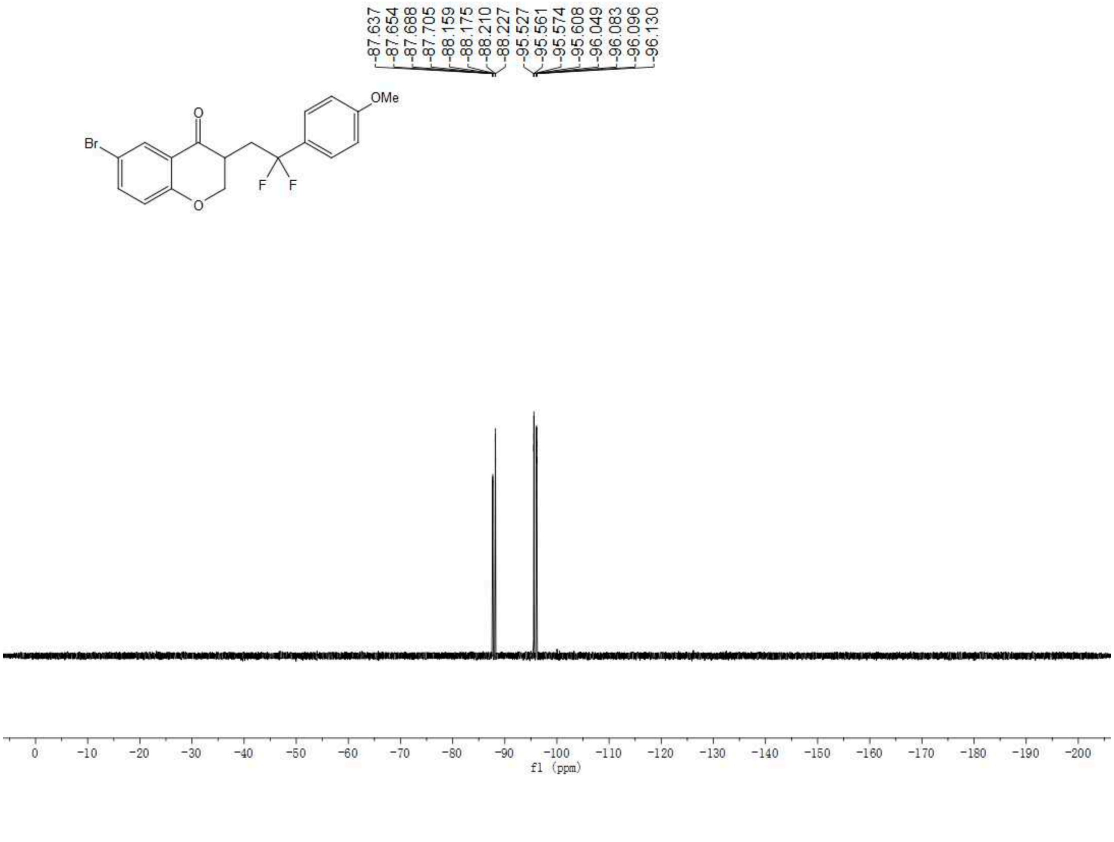

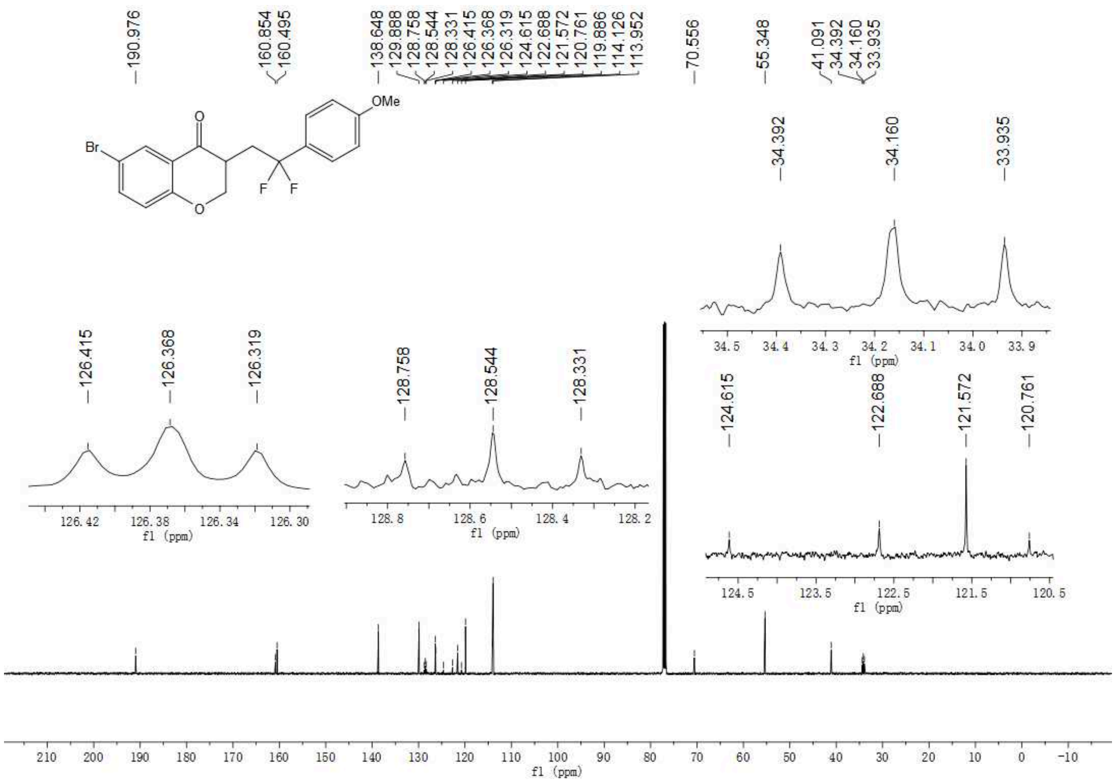

7-chloro-3-(2,2-difluoro-2-(4-methoxyphenyl)ethyl)chroman-4-one (3ib)

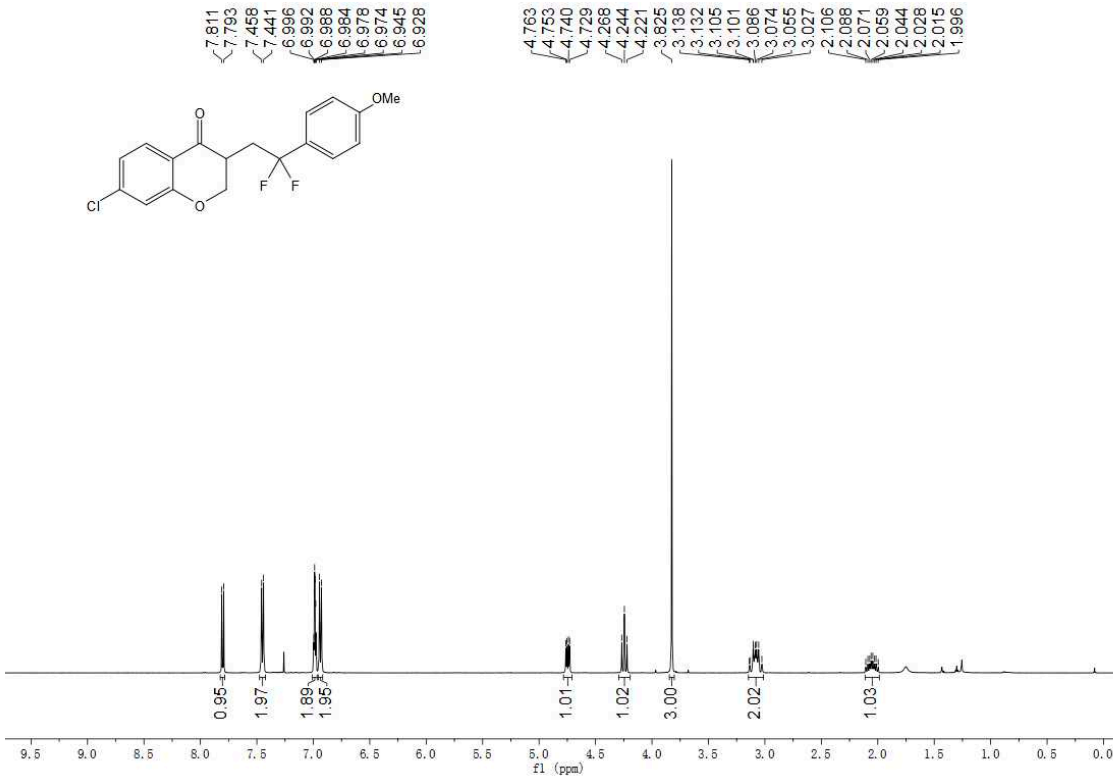

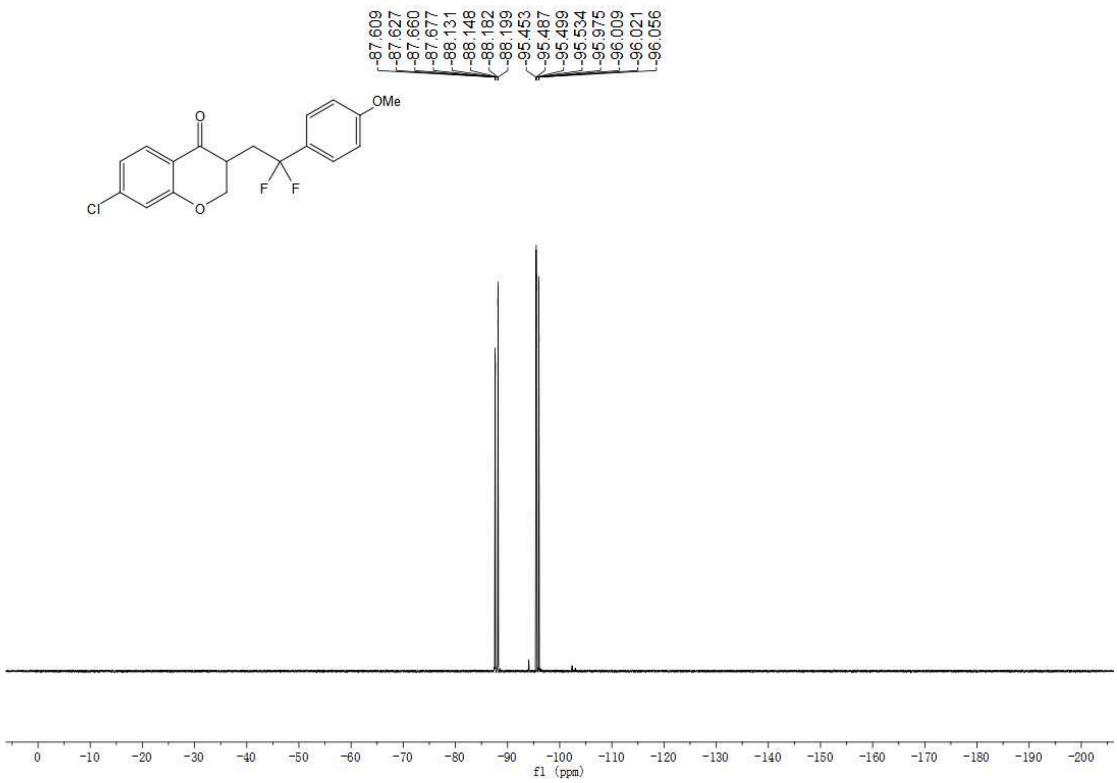
-87.609
-87.627
-87.660
-87.677
-88.131
-88.148
-88.182
-88.199
-95.453
-95.487
-95.499
-95.534
-95.975
-96.009
-96.021
-96.056
OMe
O
Cl
O
F F
0 -10 -20 -30 -40 -50 -60 -70 -80 -90 -100 -110 -120 -130 -140 -150 -160 -170 -180 -190 -200
f1 (ppm)

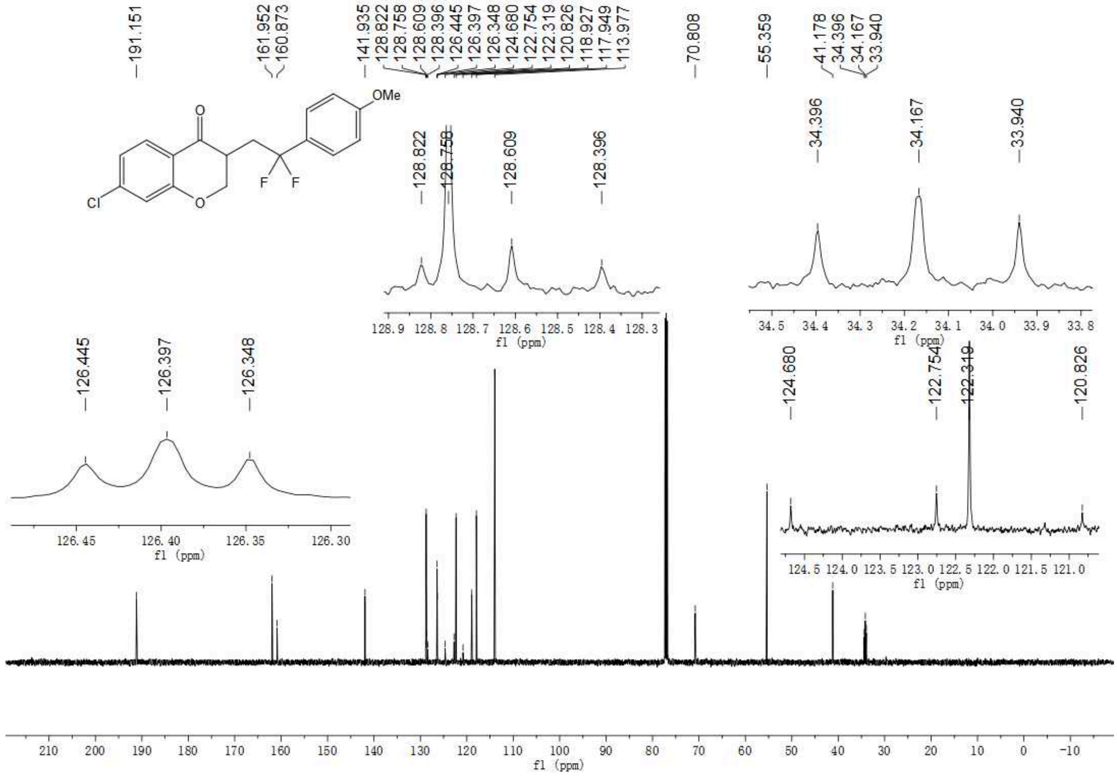
191.151
161.952
160.873
141.935
128.822
128.758
128.609
128.396
126.445
126.397
126.348
124.680
122.754
122.319
120.826
118.927
117.949
113.977
70.808
55.359
41.178
34.396
34.167
33.940
OMe
O
Cl
O
F F
128.822
128.758
128.609
128.396
128.9 128.8 128.7 128.6 128.5 128.4 128.3
f1 (ppm)
34.396
34.167
33.940
34.5 34.4 34.3 34.2 34.1 34.0 33.9 33.8
f1 (ppm)
126.445
126.397
126.348
126.45 126.40 126.35 126.30
f1 (ppm)
124.680
122.754
122.319
120.826
124.5 124.0 123.5 123.0 122.5 122.0 121.5 121.0
f1 (ppm)
210 200 190 180 170 160 150 140 130 120 110 100 90 80 70 60 50 40 30 20 10 0 -10
f1 (ppm)

8-chloro-3-(2,2-difluoro-2-(4-methoxyphenyl)ethyl)chroman-4-one (3jb)

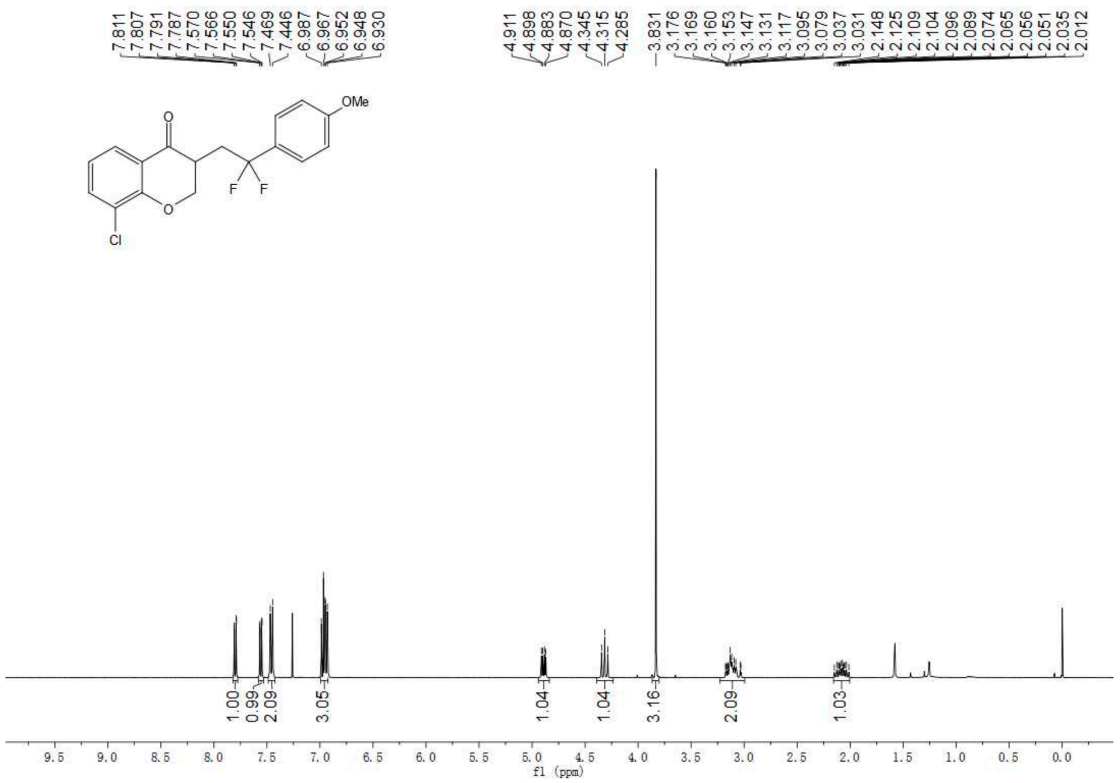

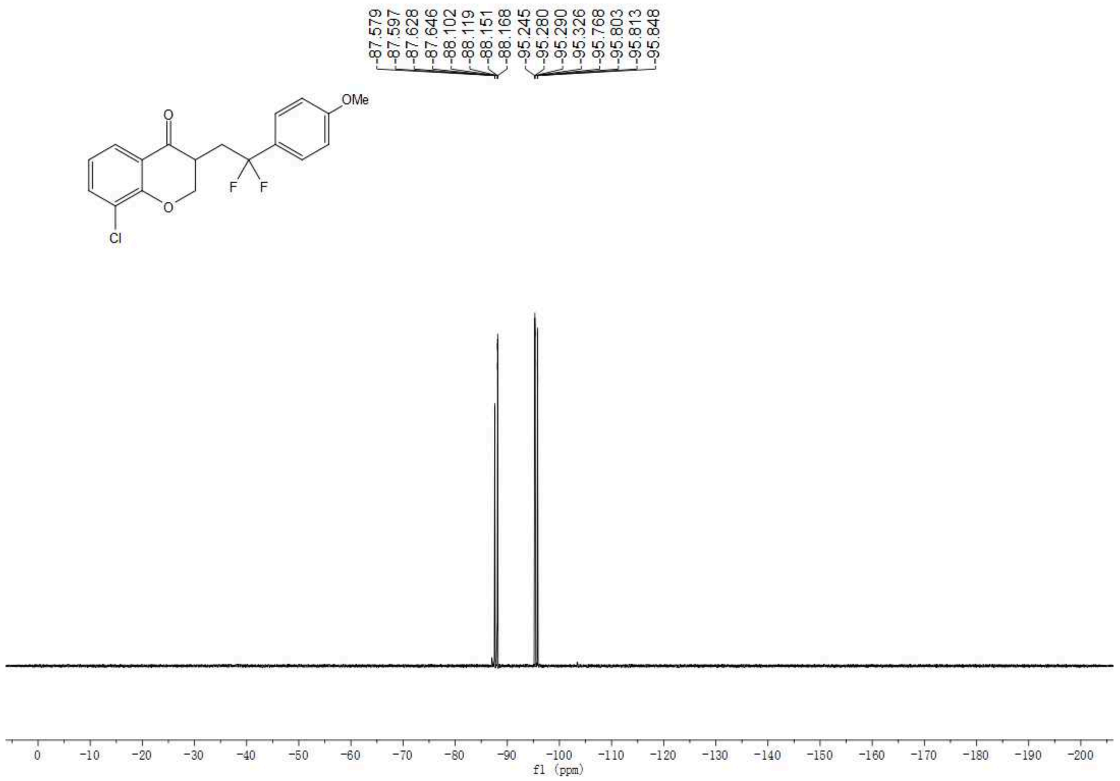

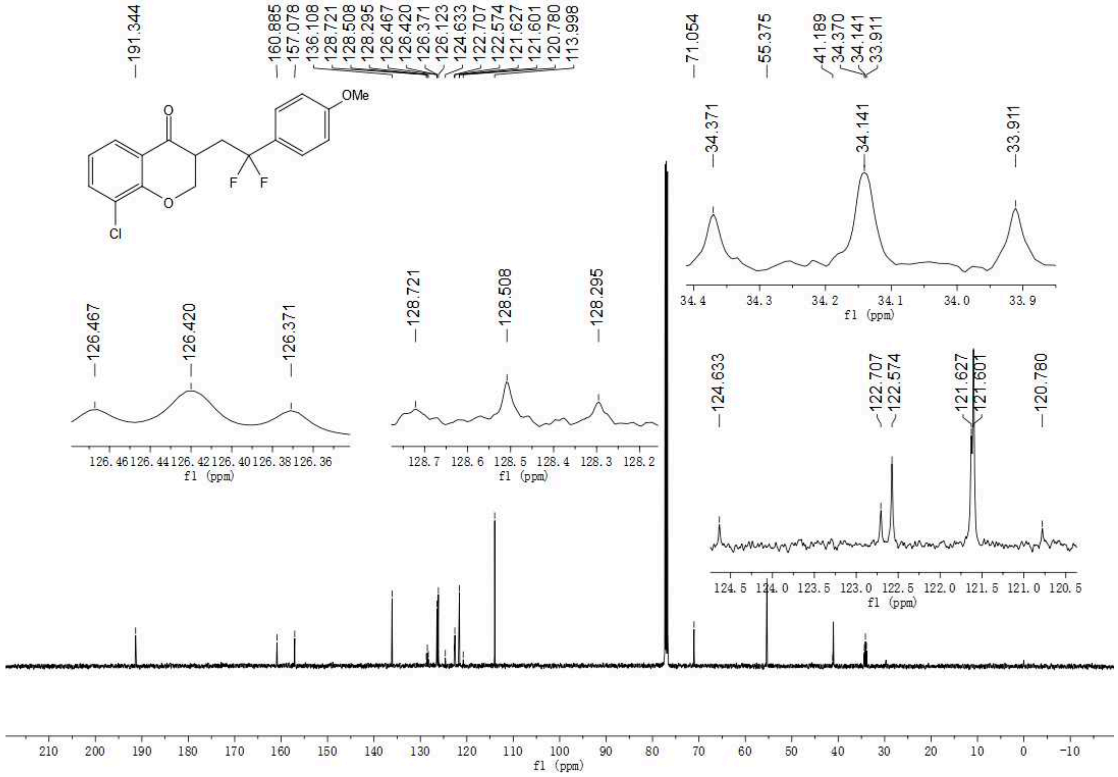

3-(2,2-difluoro-2-(4-methoxyphenyl)ethyl)-6-iodochroman-4-one(3kb)

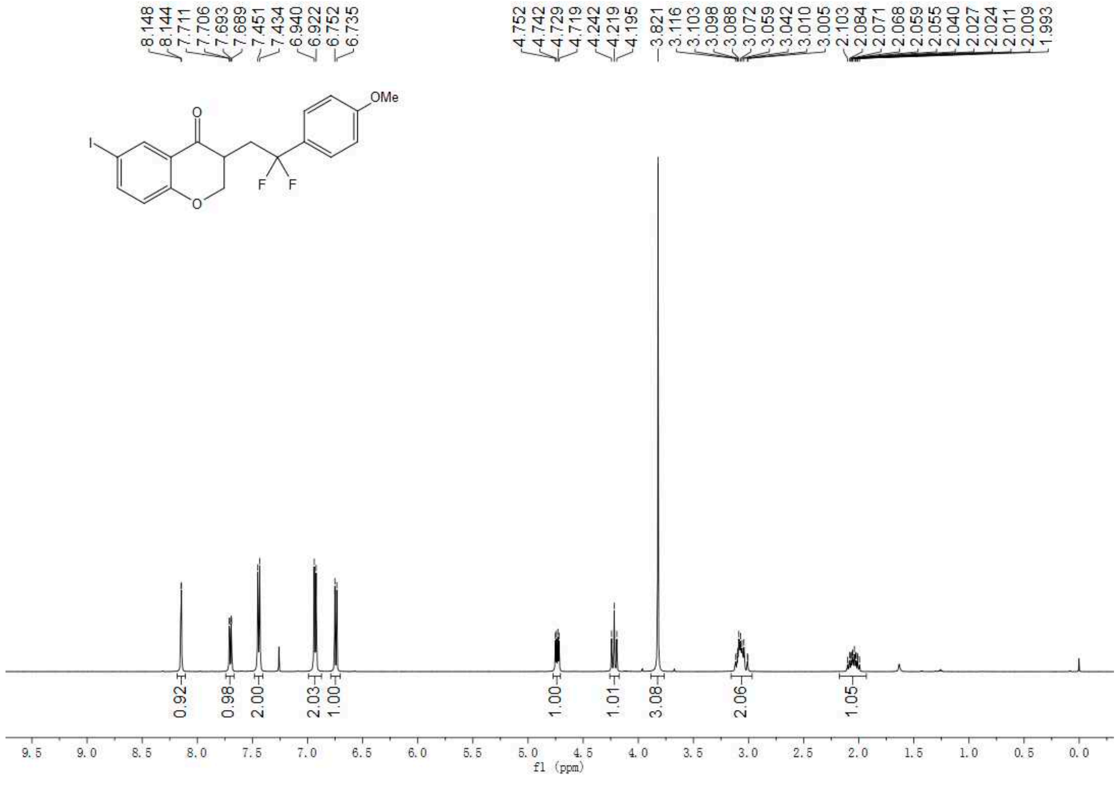

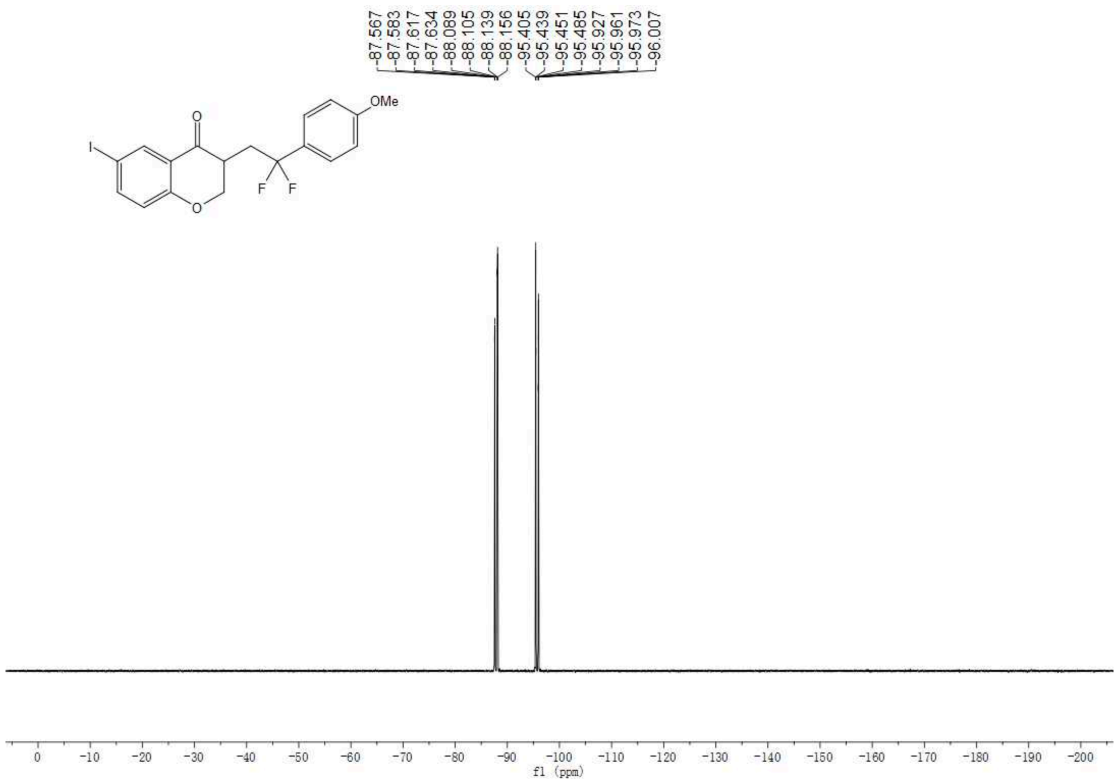

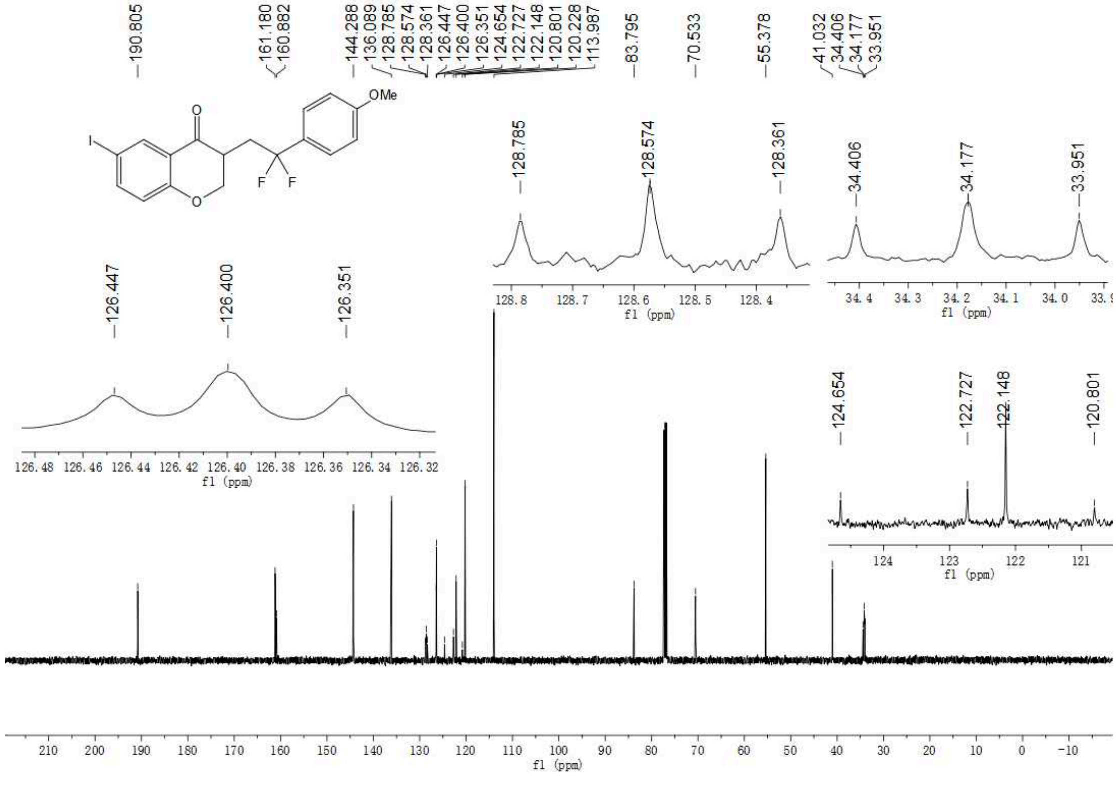

6,8-dibromo-3-(2,2-difluoro-2-(4-methoxyphenyl)ethyl)chroman-4-one (3lb)

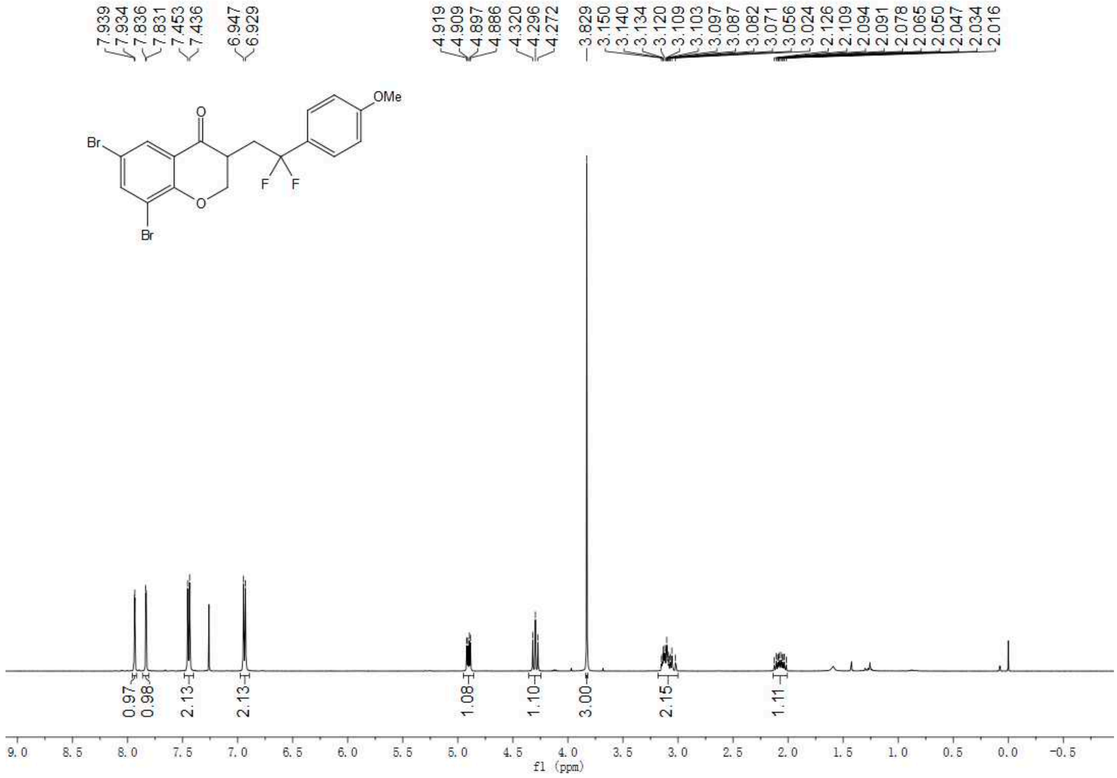
7.939
7.934
7.836
7.831
7.453
7.436
6.947
6.929
4.919
4.909
4.897
4.886
4.320
4.296
4.272
3.829
3.150
3.140
3.134
3.120
3.109
3.103
3.097
3.087
3.082
3.071
3.056
3.024
2.126
2.109
2.094
2.091
2.078
2.065
2.050
2.047
2.034
2.016
OMe
O
Br
F
F
O
Br
0.97
0.98
2.13
2.13
1.08
1.10
3.00
2.15
1.11
f1 (ppm)

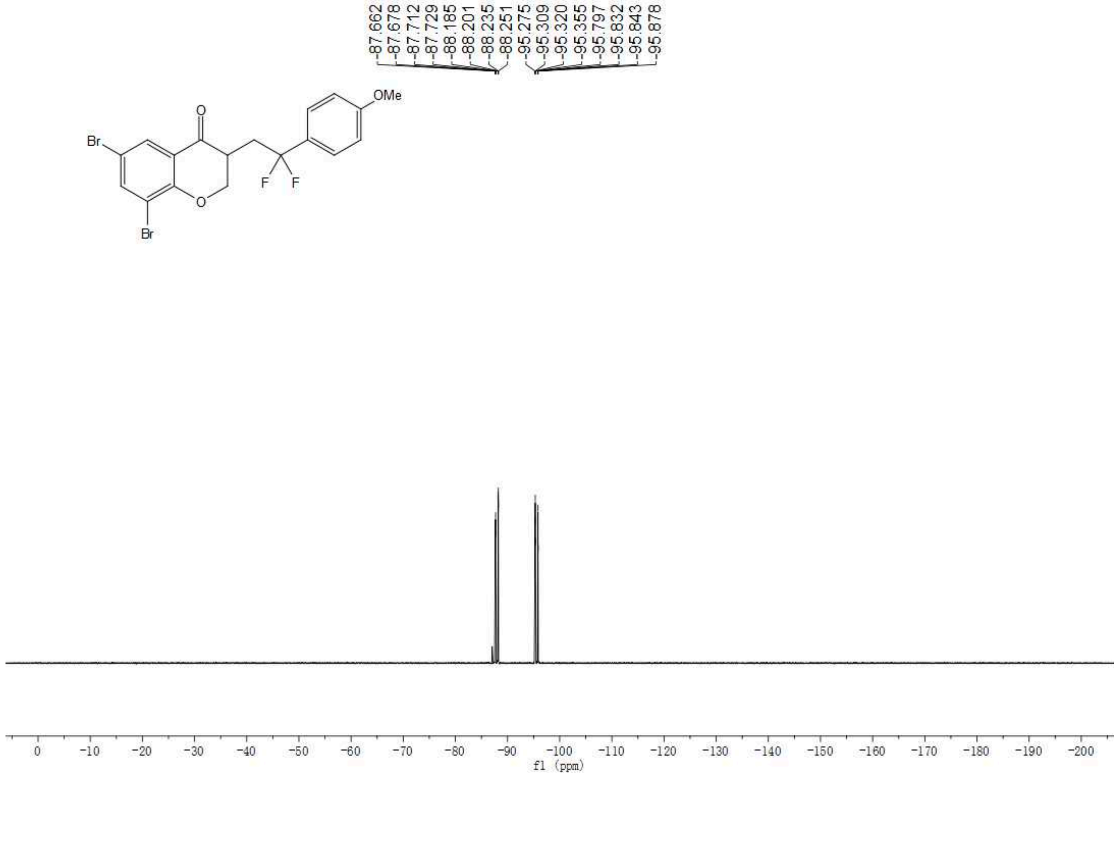
-87.662
-87.678
-87.712
-87.729
-88.185
-88.201
-88.235
-88.251
-95.275
-95.309
-95.320
-95.355
-95.797
-95.832
-95.843
-95.878
OMe
O
Br
F
F
O
Br
f1 (ppm)

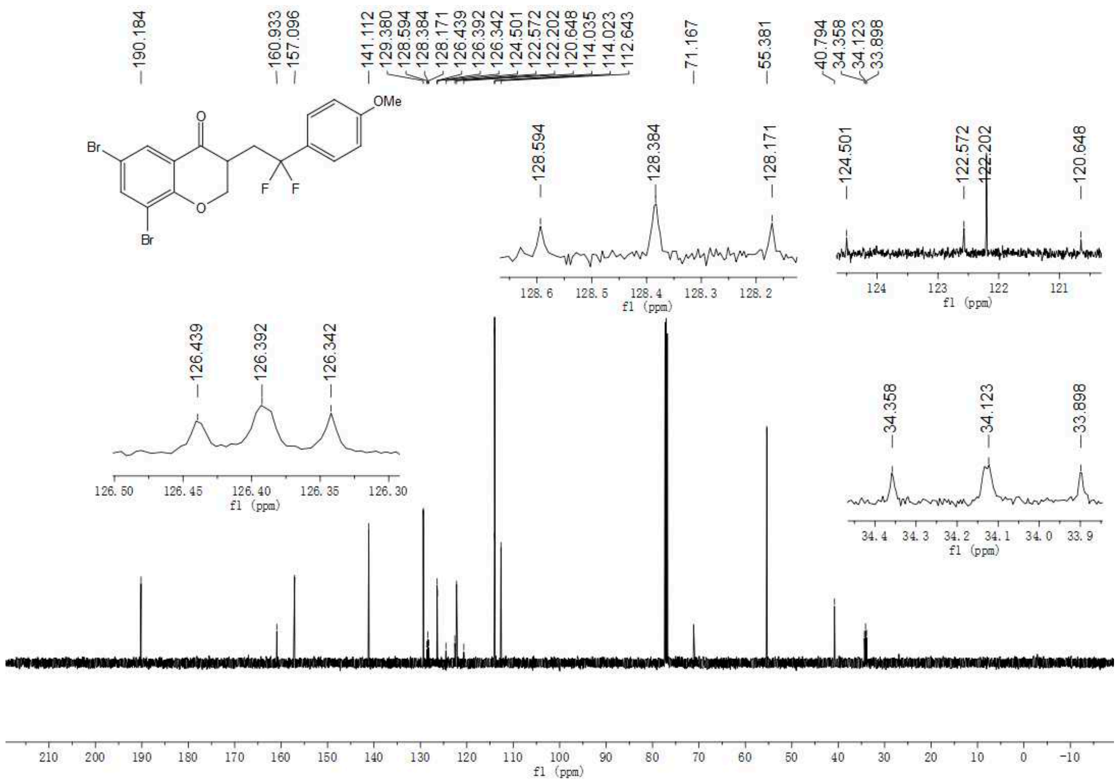

3-(2,2-difluoro-2-(4-methoxyphenyl)ethyl)-3-methylchroman-4-one (3mb)

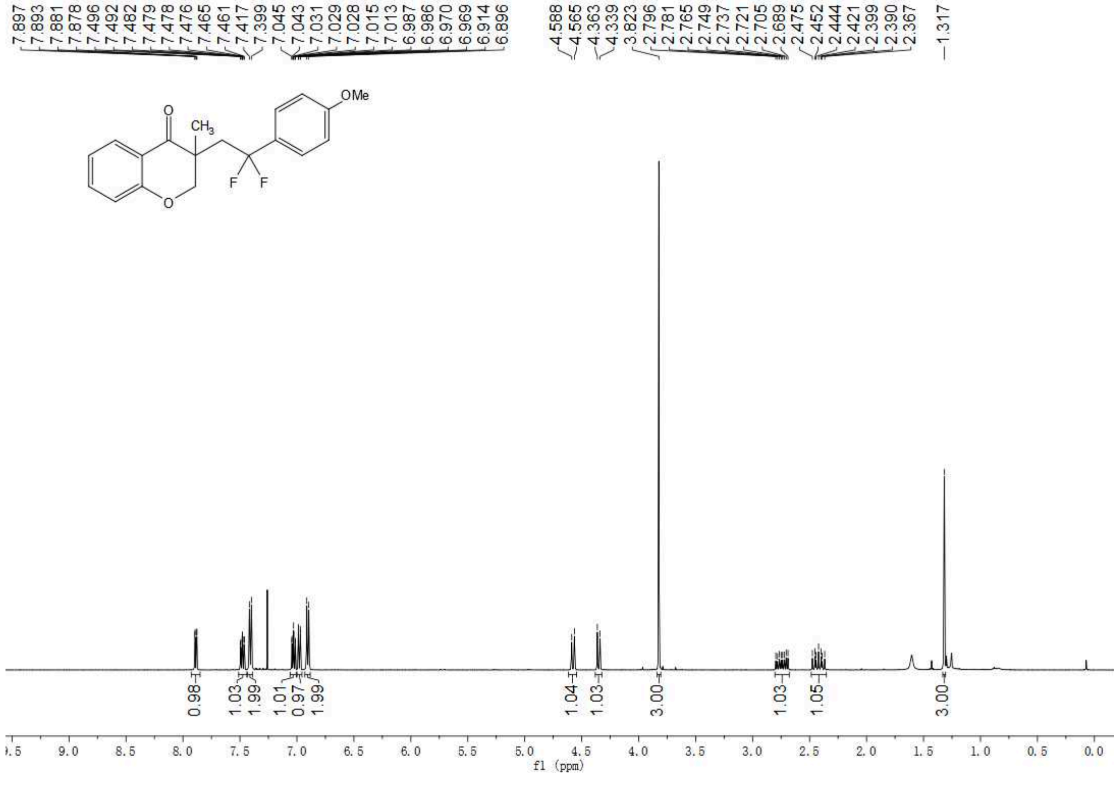

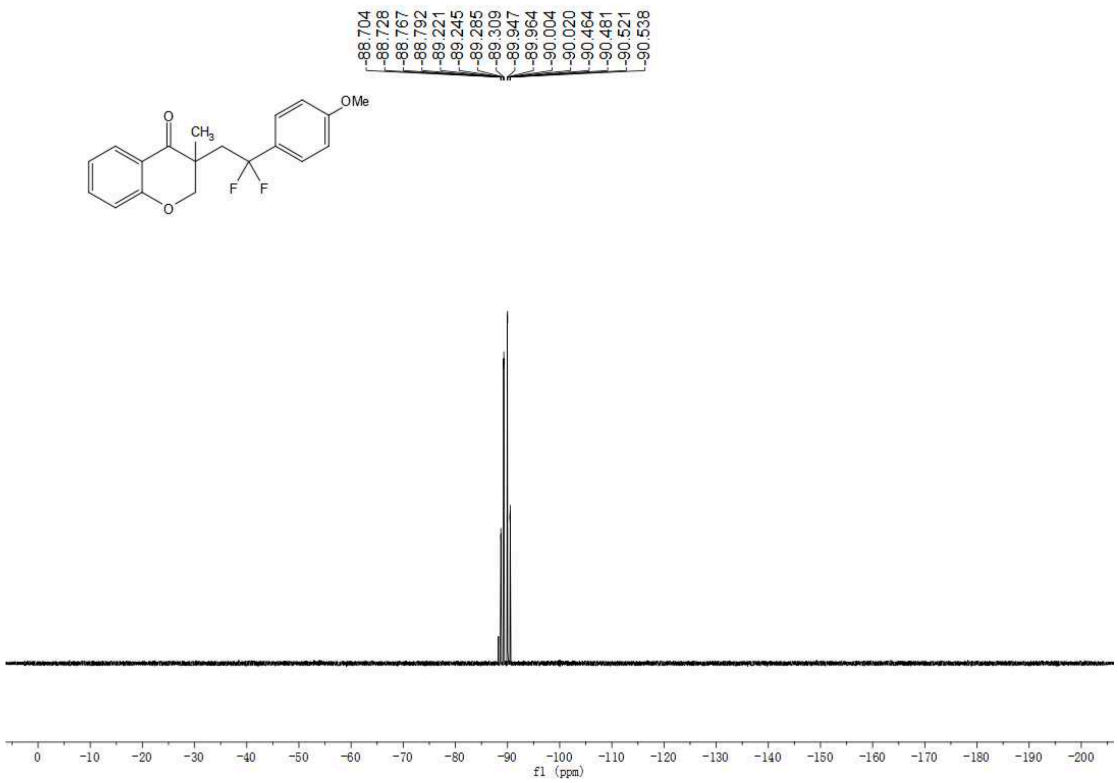

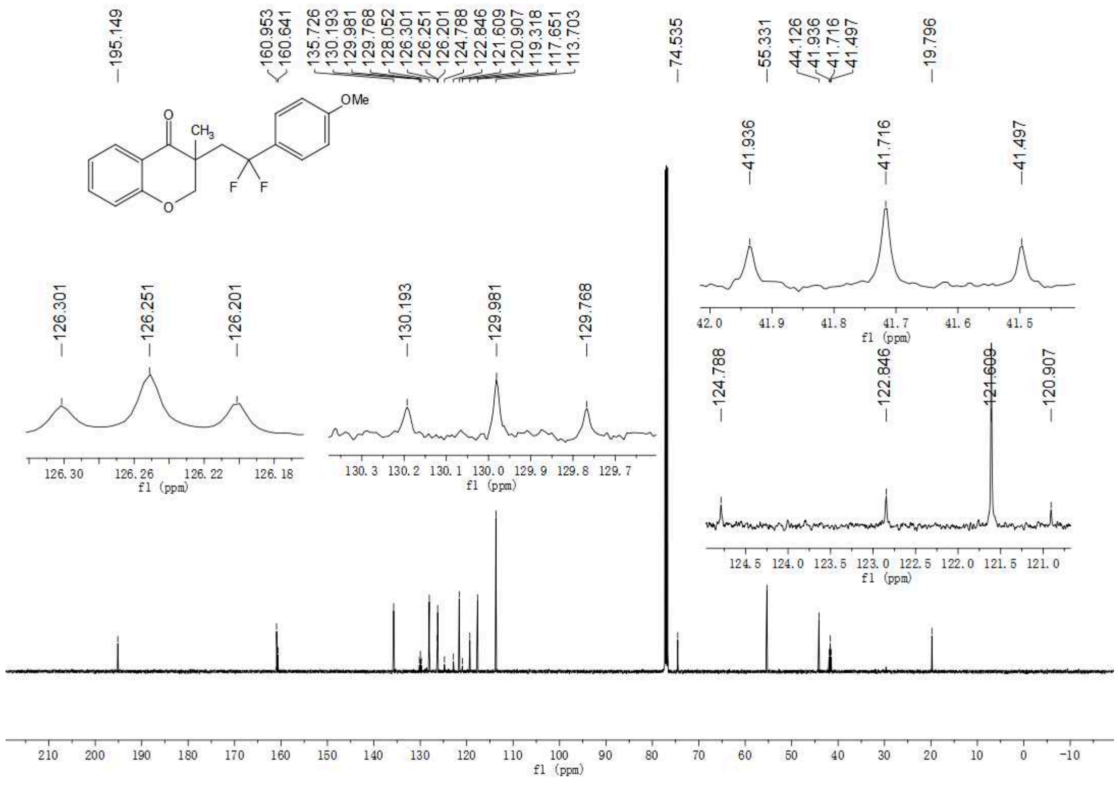

2-(2,2-difluoro-2-(p-tolyl)ethyl)-2,3-dihydro-1H-inden-1-one (3na)

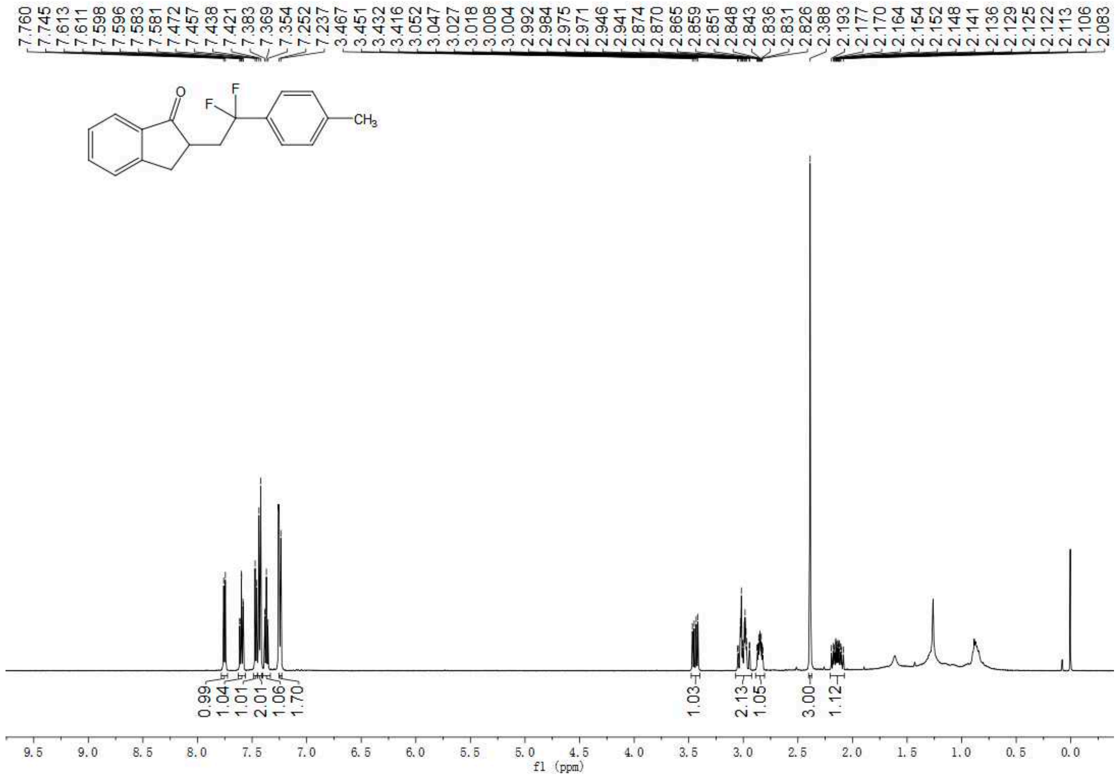

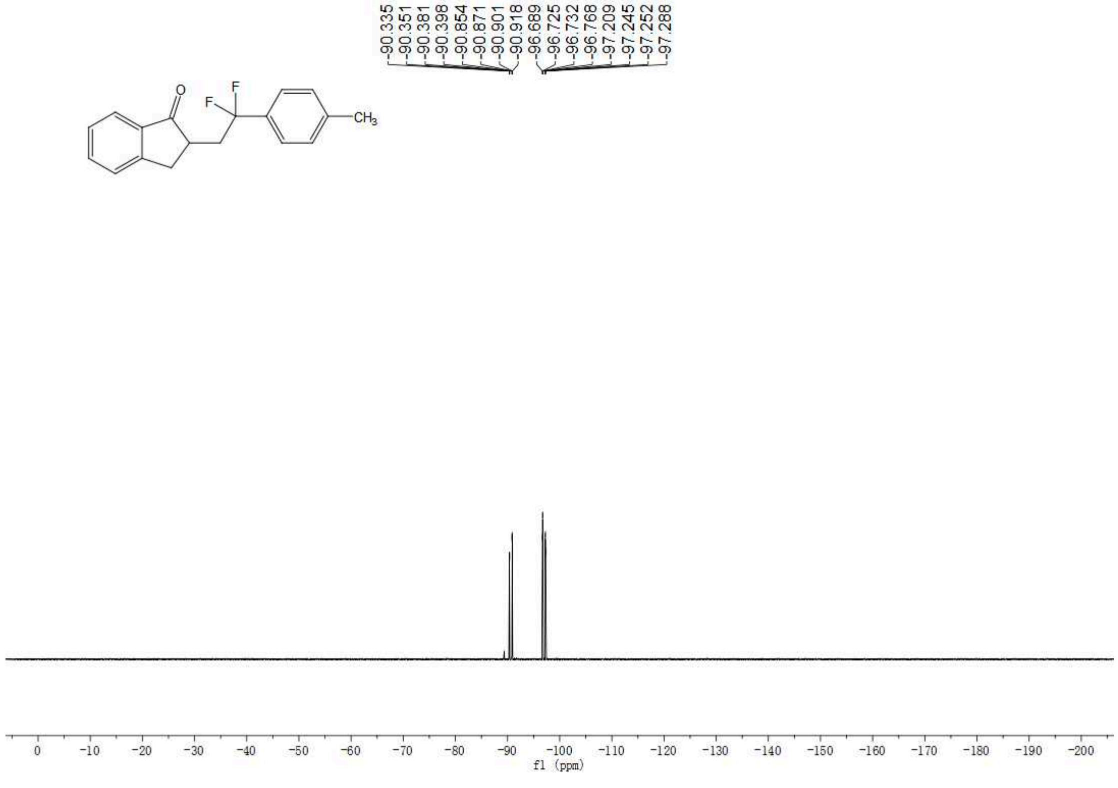

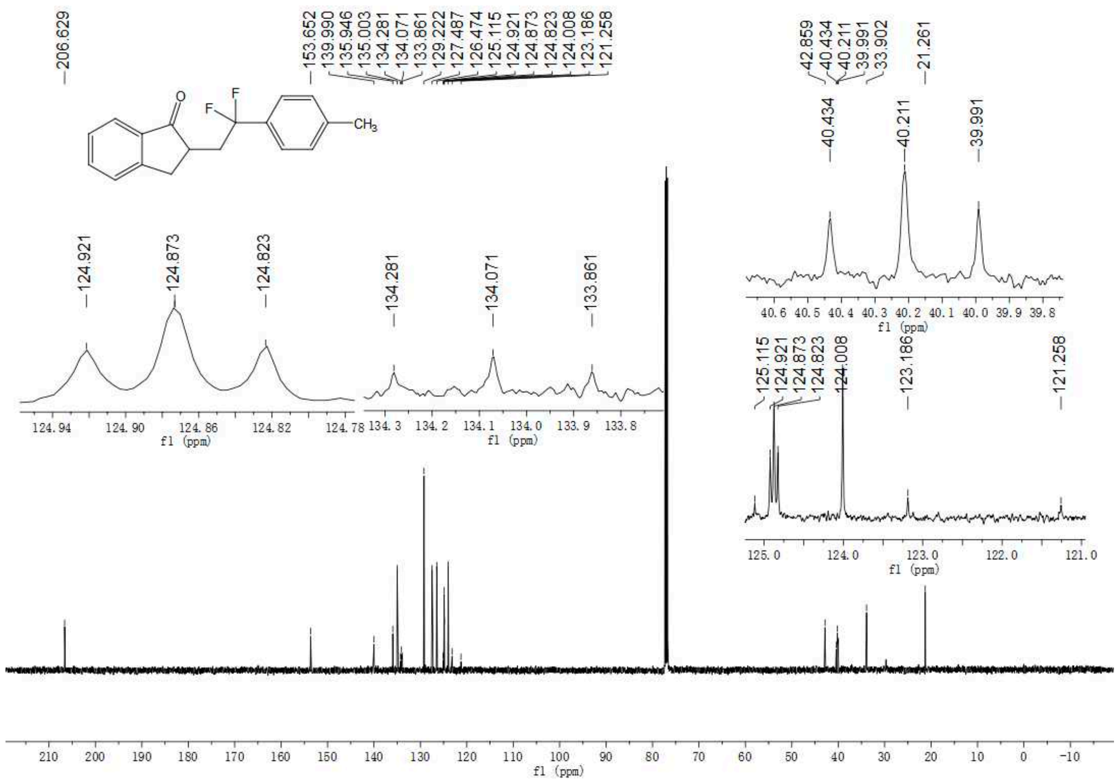

1,1,2,2-tetrafluoro-1,2-di-p-tolylethane (4)

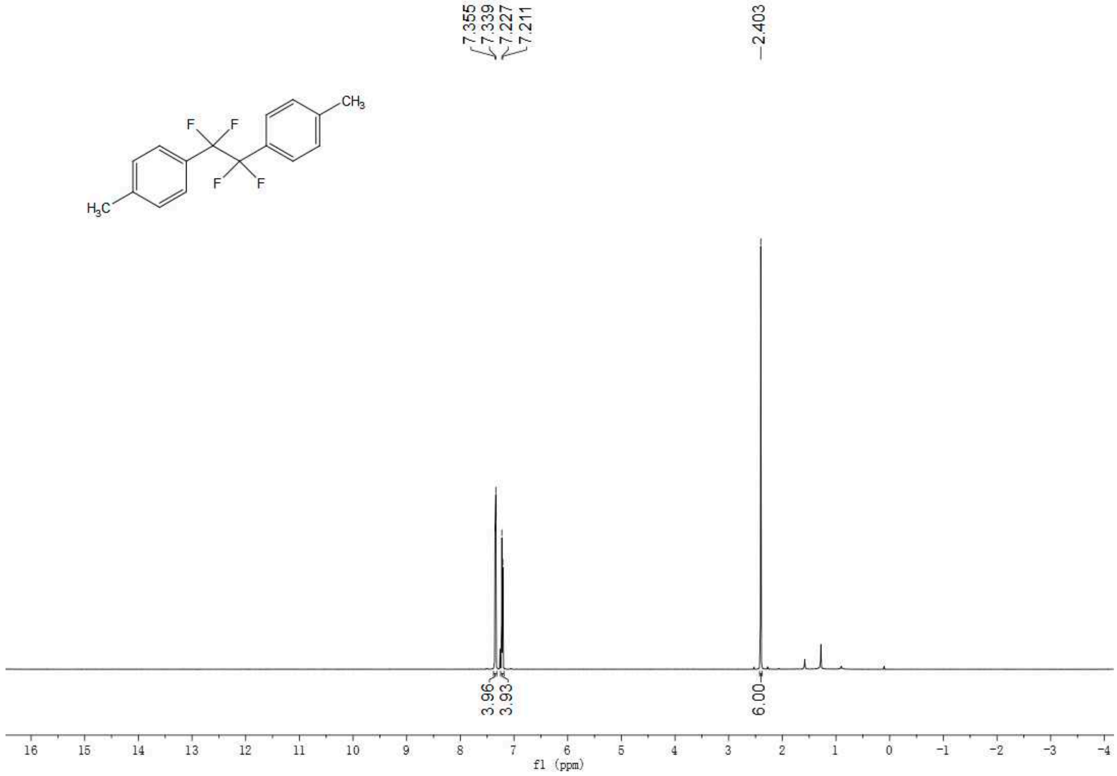

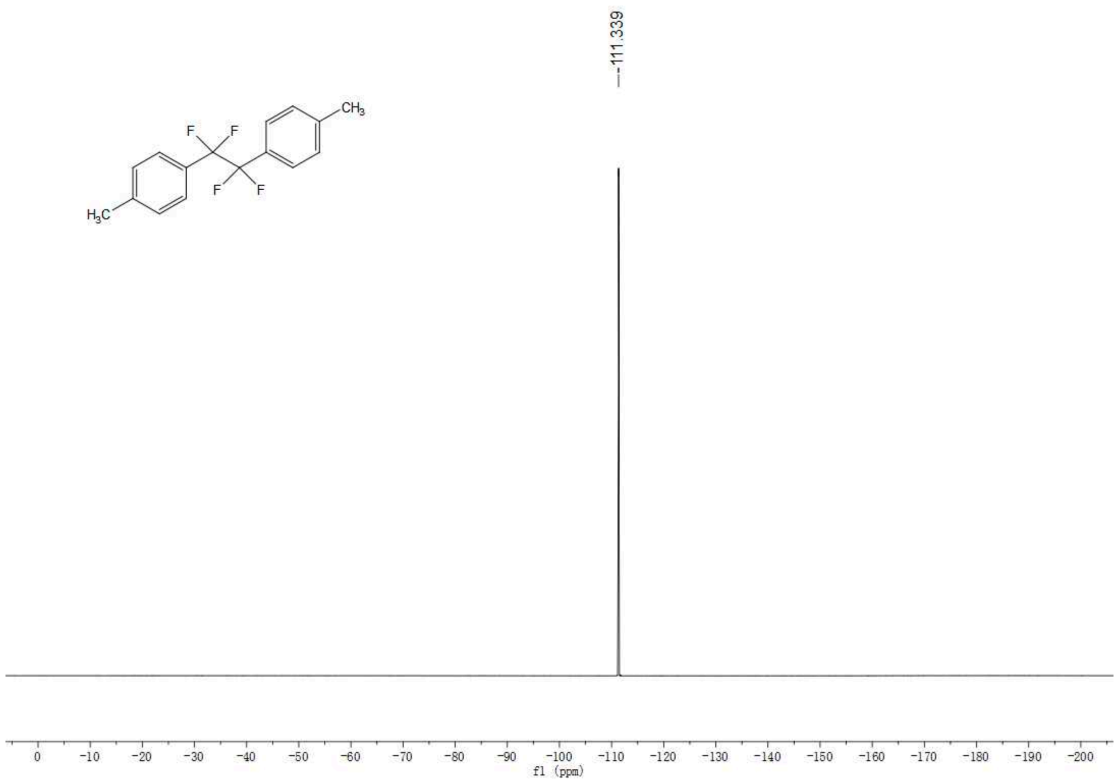

-111.339
F
F
F
F
CH3
H3C
0
-10
-20
-30
-40
-50
-60
-70
-80
-90
-100
-110
-120
-130
-140
-150
-160
-170
-180
-190
-200
f1 (ppm)
